# Mapping structure–property relationships in a 6-oxo-verdazyl radical by variable pressure crystallography and density functional theory

**DOI:** 10.1107/S2052520626006220

**Published:** 2026-06-26

**Authors:** James R. Brookes, Varshini J. Kumar, Isabelle M. Jones, Aston M. Summers, Stephanie A. Bird, Paul J. Low, Stephen A. Moggach, Dino Spagnoli

**Affiliations:** a School of Molecular Sciences University of Western Australia Perth 6009 Australia; b School of Earth & Planetary Sciences Curtin University Bentley 6102 Australia; c Australian Synchrotron 800 Blackburn Road Clayton 3168 Australia; Politecnico di Milano, Italy

**Keywords:** verdazyl radicals, high pressure, crystallography, density functional theory

## Abstract

The response of the 1,5-tolyl-3-phenyl-6-oxo-verdazyl radical (pTolOV) to pressure in the crystalline state has been investigated using both high-pressure X-ray diffraction techniques and density functional theory (DFT) calculations. Changes in structure, electronic properties such as band gap and density of states, and magnetic exchange interactions have been explored. Structural analysis reveals a phase transition between the *P*2_1_/*n* and *C*2/*c* space groups between 2.30 and 2.73 GPa, and an accompanying increase in molecular point group symmetry in the solid state structure at higher pressures. Modelling using periodic DFT-based methods reveals no significant energy penalty to this phase transition with a small reduction in the band gap upon increasing pressure due to changes in the intermolecular distances, before a sharp increase in band gap with the changes in intermolecular alignment in the *C*2/*c* phase. These variations fall in the range 2.02–1.94 eV, and the radical remains a wide-gap semiconductor in the solid state across the pressure range examined. Further DFT calculations with gas-phase models reveal weak antiferromagnetic exchange interactions occurring in antiparallel π-stacked chains in both phases.

## Abstract 2

For the first time, the structural response to pressure of a verdazyl radical crystal has been studied. Computational modelling revealed changes in electronic and magnetic properties as a result of a structural phase transition.

## 1. Introduction

Studies of the effects of external stimuli (temperature, light, pressure, *etc*.) on the properties of bistable organic radicals in the solid state have been driven by the desire to design and create cheap, lightweight, and solution processable alternatives to common electronic components such as transistors and switches (Ratera & Veciana, 2012 ▸; Chen *et al.*, 2021 ▸). To this end, candidate molecular materials with properties such as a narrow-to-medium band-gap, high electron transport properties, and/or stimuli-responsive magnetic behaviour are a source of considerable interest. Molecular solids of organic radical molecules fit this purpose, as their open-shell nature often produces enhanced electronic and magnetic properties compared to their closed-shell counterparts. These properties may be further tuned or optimized through subtle changes to the chemical structure by substitution at the molecular periphery (Eusterwiemann *et al.*, 2017 ▸). Consequently, organic radicals have been explored for potential applications in areas such as sensors (Zheng *et al.*, 2014 ▸), OLEDs (Luo *et al.*, 2022 ▸), solar cells (Zhang *et al.*, 2008 ▸) and molecular magnets (Kobayashi *et al.*, 2020 ▸). The ability for a compound to exist in two (or more) stable states that differ in magnetic, electronic or optical properties is known as bistability. Bistability in organic radicals is often prompted by a phase transition, whereby the changes to internal packing and intermolecular interactions result in a change in crystal symmetry and space group (Itkis *et al.*, 2002 ▸; Fujita *et al.*, 2002 ▸). These changes in the molecular ensemble are often accompanied by changes in molecular geometry or conformation leading to further evolution in properties. In molecular solids of organic radicals, the formation of dimers and subsequent quenching of the open-shell character within the crystal can be key to understanding this switching of properties and driving of phase transitions. Dimer formation can result from the formation of new intermolecular chemical bonds (Tse *et al.*, 2010 ▸), or more commonly in molecular solids, close intermolecular contacts between radical centres supported by weaker noncovalent interactions (Fujita *et al.*, 2002 ▸; Brookes *et al.*, 2025 ▸).

Whilst the field of practically applying periodic organic radical materials in functional devices is still in its infancy, understanding of the interactions between molecules in the solid state is a crucial component of facilitating useful properties *in situ*. A class of radicals analogous to verdazyl radicals, known as Blatter radicals, have been shown to be useful spin sources in a metal-free spintronic device (Nicolaides *et al.*, 2023 ▸). In such devices, magnetic interactions facilitated through intermolecular contacts are key to prosperous application (Nicolaides *et al.*, 2023 ▸), revealing the need to understand avenues by which these interactions can be targeted. Contacts between radical sites are also important for implementation in radical bearing polymers, which have been viewed as replacements for battery components, providing low-cost, sustainable alternatives for the next generation of electronic materials (Janoschka *et al.*, 2015 ▸). For this purpose, verdazyl radicals have been successful (Price *et al.*, 2014 ▸; Tahir *et al.*, 2025 ▸), highlighting the importance of exploring the solid state properties of these radicals for materials discovery.

**Figure 1:**
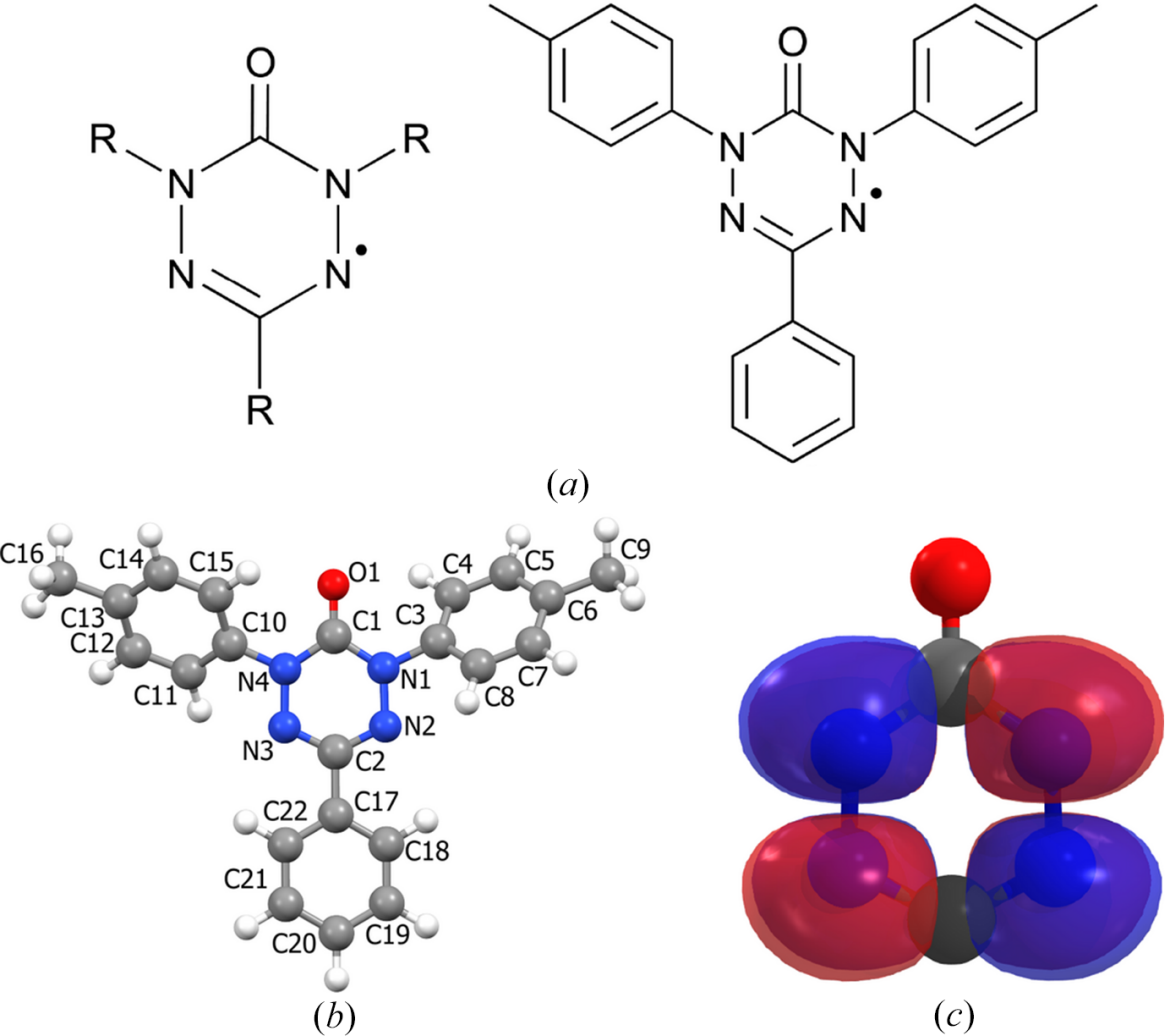
(*a*) Schematic representations of the generic 6-oxo-verdazyl core (left), the 1,5-tolyl-3-phenyl-6-oxo-verdazyl radical, pTolOV (right). (*b*) The atomic numbering used in this work. Note: hydrogen atoms are numbered according to their bonded carbon atom. (*c*) The π* SOMO of the verdazyl core.

Since their discovery by Kuhn and Trischmann (1963 ▸), verdazyl radicals (Kuhn & Trischmann, 1963 ▸; Kuhn & Trischmann, 1964 ▸), and their now common 6-oxo derivatives first reported by Neugebauer & Umminger (1980 ▸) [Fig. 1 ▸(*a*)], have been viewed as exciting examples of bench-stable organic radicals and candidates for organic magnets. Verdazyl radicals are stabilized by the delocalization of the unpaired electron across the four nitrogen atoms giving a singly occupied molecular orbital (SOMO) with π* (antibonding) character [Fig. 1 ▸(*c*)]. Spin density is predominantly located on the four nitrogen atoms, however, there is residual negative spin density located at the C3 and C6 positions of the verdazyl core (Hicks *et al.*, 2001 ▸). The synthetic pathways to both Kuhn verdazyls (Jobelius *et al.*, 2018 ▸) and 6-oxo-verdazyl radicals have been well studied (Kumar *et al.*, 2022 ▸; Naghibi *et al.*, 2022 ▸), with a wide range of derivatives synthesized for applications across chemistry and materials science.

The addition of bulky groups at the 1 and 5 positions of the 6-oxo-verdazyl centre such as thioanisole allows for binding to gold electrodes to form single-molecule junctions, in which case the open-shell nature was shown advantageous for conductance in comparison to the closed-shell configuration (Naghibi *et al.*, 2022 ▸). A popular avenue of verdazyl chemistry sees them used as ligands in coordination complexes for potential molecular magnets. In this environment they have been shown to exhibit strong ferromagnetic and antiferromagnetic intramolecular interactions (Hicks *et al.*, 2001 ▸; Gilroy *et al.*, 2006 ▸; Brook, 2015 ▸; Lipunova *et al.*, 2022 ▸). Understanding the interactions between verdazyl radicals in the solid state can be crucial to gaining insights of their behaviour in coordination complexes, as the correlation between structure and magnetic or electrical properties becomes important.

Antiferromagnetic interactions typically dominate within verdazyl radical crystals, however ferromagnetic interactions have been observed, particularly at low temperatures (Plater *et al.*, 2006 ▸; Eusterwiemann *et al.*, 2018 ▸; Tomiyoshi *et al.*, 1994 ▸; Merhi *et al.*, 2014 ▸). Although magnetic interactions between verdazyl radicals in the solid state often occur without direct SOMO–SOMO (π*–π*) overlap, a factor that would usually indicate ferromagnetic interactions according to traditional models, examples of direct SOMO–SOMO overlap between verdazyls has been observed in minimally substituted compounds such as 1,3,5-trimethyl-6-oxo-verdazyl and 1,5-dimethyl-6-oxo-verdazyl radicals (Rosokha *et al.*, 2010 ▸). These interactions are far less common in verdazyl radicals than in other examples of organic radicals, such as dithiadiazolyl radicals that commonly exhibit strong intermolecular interactions and ‘pancake’ bonding (Strydom & Haynes, 2024 ▸). Magnetic bistability in verdazyl radicals is rare but has been observed in a charge transfer salt containing the triphenyl verdazyl radical and 2,3,5,6-tetrafluoro-7,7,8,8-tetracyano quinomethane (Lai *et al.*, 2023 ▸).

With a view to exercising control over the electrical, optical or magnetic properties of molecular materials linked to a change in molecular or solid state structure arising from an applied external stimulus, the temperature response of the properties of crystalline solids including radicals is often explored. For example, the radical TTTA (1,3,5-trithia-2,4,6-triazapentalenyl) was shown to undergo a phase transition under increasing temperature with associated magnetic bi­stability, from a diamagnetic low-temperature (space group *P*1) phase to a high-temperature (*P*2_1_/*c*) paramagnetic phase with hysteresis occurring between 230 and 305 K (Fujita *et al.*, 2002 ▸). Another example of radical polymorphism and subsequent changes in property induced by variable temperatures is the boron containing phenalenyl radical of Itkis and coworkers, that exhibits magnetic, electrical and optical bistability due to reversible dimer formation between 320 and 350 K (Itkis *et al.*, 2002 ▸).

The effect of pressure on radical molecular solids is a topic of growing interest with a view to both mapping structure–property relationships and developing pressure-sensitive and pressure-responsive molecular materials. TTTA has been studied under external pressures, where the application of pressure was shown to increase the hysteresis range from 230 to 305 K at 0 GPa to ∼280–330 K at 1.5 GPa and a reduction in magnetic susceptibility of the paramagnetic phase (Tanaka *et al.*, 2004 ▸). The structural response to pressure of TTTA has also been studied, with no phase transition found for the high-temperature phase up to 4.65 GPa, illustrating that the transition from paramagnetic to diamagnetic can be attributed to a decrease in radical separation (Richardson *et al.*, 2021 ▸). Metallization of organic radicals has been observed under applied pressure, through a series of thiazolyl and thiaselenazolyl radicals undergoing phase transitions and band-gap closures between 3 and 13 GPa, prompted by buckling of radical dimers (Tse *et al.*, 2010 ▸; Lekin *et al.*, 2012 ▸; Wong *et al.*, 2014 ▸). However, these examples are notable exceptions as few of the organic radical structures deposited in the Cambridge Structural Database (CSD; Groom *et al.*, 2016 ▸) have been determined above ambient pressures.

In many organic radical species including verdazyls, stability is improved by the addition of bulky steric groups, which provide spatial separation between reactive radical sites. However, separation of radical centres can hinder important interactions that control magnetic and electrical properties (Plater *et al.*, 2006 ▸). By applying external hydrostatic pressure to organic radical solids, magnetic interactions may be prompted by reducing separation between radical centres and overcoming the steric bulk that provides ambient stability. There are approximately 70 verdazyl radical entries on the CSD (Groom *et al.*, 2016 ▸) (not including salts, cocrystals and coordination complexes), and there is yet to be an investigation of their structure under an applied external pressure. Therefore, this area of solid state molecular electronic research is ripe for exploration.

In this work, we set out to explore the response of the molecular and crystal structures of the 1,5-tolyl-3-phenyl-6-oxo-verdazyl radical (pTolOV; Fig. 1 ▸) to external pressure and the subsequent changes in electronic structure. The pTolOV radical was initially synthesized, in part, for applications in single molecule electronics, and was shown to undergo characteristic single electron reduction and oxidation, as well as having EPR spectra consistent with other oxo-verdazyl radicals (Kumar *et al.*, 2022 ▸). EPR spectra of oxo-verdazyl radicals have confirmed the π delocalization of the unpaired electron across the four nitrogen centres, with coupling to the *R* substituents.

We present here the crystal structure of the pTolOV radical at pressures between ambient pressure and 3.31 GPa and its resulting phase transition and investigate changes in molecular structure and crystal packing. We subsequently present theoretical band structures and density of states (DOS), using periodic density functional theory (DFT). These results were supplemented further by gas-phase DFT calculations to explore changes in magnetic exchange interactions in response to the observed structural variation. This is the first high-pressure (HP) study of a verdazyl radical solid, unlocking a new exciting avenue for discovery of molecular electronic materials.

## 2. Experimental

pTolOV was synthesized as previously reported by Kumar *et al.* (2022 ▸) and crystallized through slow evaporation of a solution in toluene.

### 2.1. X-ray crystallography

An ambient-pressure X-ray diffraction (XRD) experiment was performed on pTolOV at 295 K using a Rigaku XtaLAB Synergy-S diffractometer, Cu *K*α radiation (1.54056 Å) and a Hypix-6000HE detector. To obtain HP crystal structures, a single crystal of pTolOV was placed in a micro-modified Merrill–Basset diamond anvil cell (DAC) (Moggach *et al.*, 2008 ▸), with a half opening angle of 40°. Within the cell the crystal was housed in a tungsten gasket with a 300 µm-diameter sample chamber, along with a small ruby chip. The ruby chip was placed in the DAC to measure the pressure using the ruby fluorescence method (Piermarini *et al.*, 1975 ▸). Fluorinert FC-70 oil was also placed in the sample chamber as a pressure-transmitting medium to ensure hydrostatic compression. The pressure was increased incrementally and XRD experiments were performed at 0.15, 0.29, 0.65, 1.22, 1.58, 2.30, 2.73 and 3.31 GPa using the HP facilities on the MX1 beamline at the Australian Synchrotron (Boer *et al.*, 2023 ▸; Cowieson *et al.*, 2015 ▸). *CrysAlisPro* was used for data integration and reduction (Rigaku Oxford Diffraction, 2014 ▸). *SADABS* (Krause *et al.*, 2015*a* ▸) was used to apply a multi-scan absorption correction (Krause *et al.*, 2015*b* ▸). Structures were solved with *SHELXT* (Sheldrick, 2015*b* ▸) and refined with *SHELXL* (Sheldrick, 2015*a* ▸) using the *OLEX2* software package (Dolomanov *et al.*, 2009 ▸). The DAC opening angle was restricted to 35° during data integration to compensate for shading caused by the gasket.

During refinement of the HP data, 1–2 and 1–3 atom distances were restrained to ambient values, allowing for torsion angle rotation and flexing within the molecules whilst compensating for low completeness caused by shading from the DAC. The oxo-verdazyl core, tolyl moieties and the phenyl ring were constrained as rigid bodies. Due to low completeness, only the oxygen atom was refined with anisotropic displacement parameters for HP data. An extinction correction was applied during refinement of the ambient and HP data. All HP data was cut to 1 Å resolution to improve refinement models due to weak data quality at high angles. Limitations in available high-angle data caused by physical shading from the DAC gasket necessitates the use of a 1 Å resolution limit. Such a resolution limit retains integrity in the structural model while including as much data as possible. The last pressure point, 3.31 GPa, required the use of *XPREP* (Bruker, 2014 ▸) to obtain the correct space group. At the highest pressure point (3.31 GPa), sample degradation resulted in poor diffraction data, making automated space group determination difficult; consequently, *XPREP* was used to verify and assign the correct *C*2/*c* space group. See Section S1 of the supporting information for the full experimental and refinement details.

### 2.2. Computational details

#### 2.2.1. Periodic-DFT

The geometry of the finalized crystal structures were optimized and electronic structure determined through band structure and DOS calculations using the *VASP* software package (versions 6.3.0 and 6.3.2) (Kresse & Hafner, 1993 ▸; Kresse & Furthmüller, 1996*b* ▸; Kresse & Furthmüller, 1996*a* ▸). To model core electrons, the projector augmented wave (PAW) method (Kresse & Joubert, 1999 ▸) was utilized with hard pseudopotentials (Kresse & Hafner, 1994 ▸) and an energy cutoff of 1000 eV. To save computational cost where possible, the HP phase structures were transformed to their primitive cell, the unit-cell parameters for which can be found in Section 3.2. *k*-point determination was achieved through the inbuilt automatic *k*-point mesh method, creating a Γ-centred mesh with a length of 40 (*R*_*k*_) defining the subdivisions of the reciprocal space. The individual *k*-point meshes for each pressure point is supplied in the supporting information (Section S3). Geometry optimizations were completed using the RPBE (Hammer *et al.*, 1999 ▸) functional with the D3BJ dispersion correction (Grimme *et al.*, 2010 ▸; Grimme *et al.*, 2011 ▸). RPBE was chosen due to its general applicability and reliability for a variety of purposes (Hafner, 2008 ▸). Dispersion forces were accounted for with the D3BJ dispersion correction, the D3 correction of Grimme and coworkers with Becke–Johnson damping (Grimme *et al.*, 2010 ▸; Grimme *et al.*, 2011 ▸). This correction improves the accuracy of calculations involving molecular solids with large dispersion and long-range interactions (Grimme *et al.*, 2010 ▸; Grimme *et al.*, 2011 ▸). Experimental structures of pTolOV were used as the starting structure for a three step geometry optimization procedure. The first step involves optimization with a fixed unit cell, followed by an optimization of the cell contents and unit cell (starting from the previous steps coordinates). This method, from van de Streek and Neumann (2010 ▸), has been shown to increase accuracy and the ability to optimize to the global minimum. The final step takes the geometry of the previous step and runs a single-point energy calculation without optimization, as recommended by the *VASP* developers for accurate total energies (*VASP*wiki: Volume relaxation. https://vasp.at/wiki/Volume_relaxation). Experimental pressures were applied at each optimization step with the inbuilt PSTRESS tag.

All calculations were completed with spin-polarization assuming an antiferromagnetic configuration, an assumption made based on the magnetic properties of the majority of verdazyl radicals studied to date. The configuration was implemented using the inbuilt MAGMOM tag, applying either a magnetic moment of +1 or −1 on one nitrogen per molecule, alternating to give a total magnetic moment of 0. Such a configuration was shown to match the experimental magnetic data of the 2,4,6-triphenyl-verdazyl radical (Tomiyoshi *et al.*, 1994 ▸). To avoid inaccurate spin density, the magnetic moment of each atom was allowed to optimize, resulting in a net unit-cell magnetic moment of 0 in all cases.

Electronic structure calculations (band structure and DOS), were completed using the hybrid HSE06 functional (Krukau *et al.*, 2006 ▸). HSE06 is often considered the gold standard for electronic structure calculations, and is shown to perform well in the prediction of band gaps in crystalline solids (Garza & Scuseria, 2016 ▸). Due to its large computational cost, some alterations to the computational method were required compared to the geometry optimization process. The energy cutoff was reduced to 700 eV and regular pseudopotentials were used, compared to a 1000 eV cutoff and hard pseudopotentials of the optimization method. Calculation of a full band structure also bears a significant computational cost to the extent that it is not feasible to calculate for a system of this size with the computational resources afforded to us. To serve as a proxy for the band gap, the band gap at the Γ-point was used instead, determined from a single-point energy calculation with only the Γ *k*-point. DOS were calculated with a single-point energy calculation utilizing the *VASPk*-point mesh generation method with length *R*_*k*_ = 20. To improve accuracy of the calculated DOS, the number of grid points was increased to 800 from the default 301 with the NEDOS tag in *VASP*. The *sumo* command-line tools were used to plot the results of DOS calculations (Ganose *et al.*, 2018 ▸).

#### 2.2.2. Gas-phase DFT

A rigid torsion angle rotation scan was completed at the ROHF DSD-PBEP86-D3BJ/def2-SVP (Kozuch & Martin, 2011 ▸; Kozuch & Martin, 2013 ▸; Grimme *et al.*, 2010 ▸; Grimme *et al.*, 2011 ▸; Weigend & Ahlrichs, 2005 ▸) level of theory in *Gaussian16* (revision A.02) (Frisch *et al.*, 2016 ▸). Refer to Section S4 of the supporting information for full computational details. This was completed with an earlier structural refinement of pTolOV, although the structure does not differ significantly. Cartesian coordinates of the input structure and torsion angles are provided in the supporting information. Magnetic calculations were completed with UB3LYP/6-31+G(d) (Vosko *et al.*, 1980 ▸; Lee *et al.*, 1988 ▸; Becke, 1993 ▸; Stephens *et al.*, 1994 ▸; Ditchfield *et al.*, 1971 ▸; Hehre *et al.*, 1972 ▸; Hariharan & Pople, 1973 ▸; Clark *et al.*, 1983 ▸) utilizing the broken-symmetry approach (Noodleman, 1981 ▸; Noodleman & Davidson, 1986 ▸) in *ORCA* (version 5) (Neese, 2022 ▸). Further details will be provided as the results are presented.

## 3. Results

### 3.1. Crystallographic results

#### 3.1.1. Ambient-pressure structure and response to pressure

**Figure 2:**
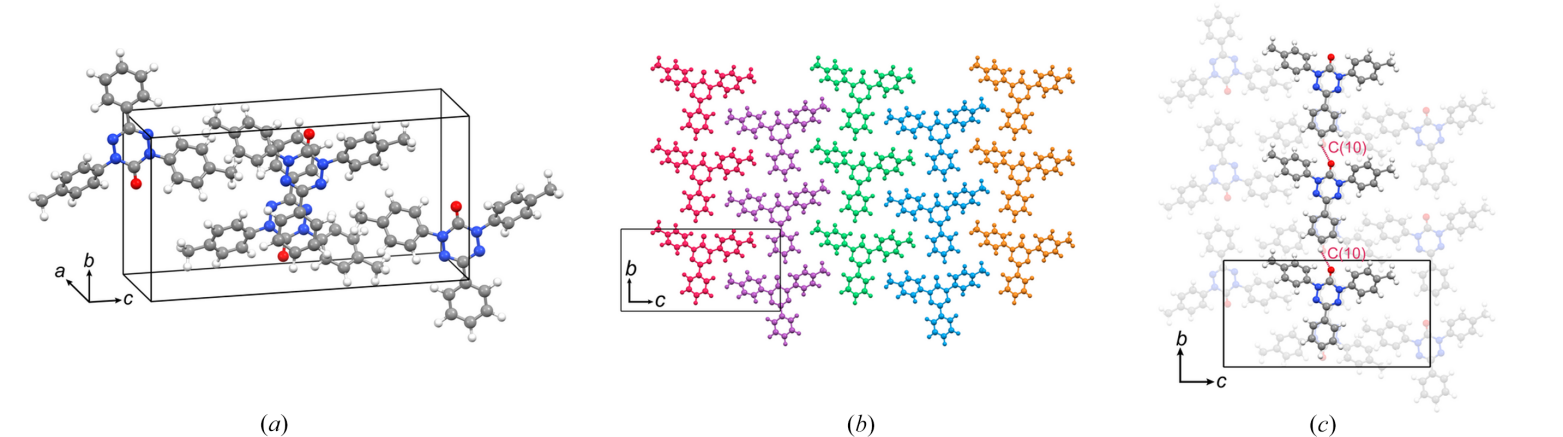
(*a*) The unit cell of pTolOV under ambient conditions (0 GPa and 295 K). (*b*) The herringbone-like configuration in the *bc* plane. (*c*) The *C*(10) hydrogen-bond chain running in the *b* direction.

At ambient pressure and temperature, pTolOV crystallizes in the monoclinic space group *P*2_1_/*n* (Table 1 ▸). The molecular bond lengths have been described previously in the literature, and a structure deposited to the CSD (Groom *et al.*, 2016 ▸) (code MASFAR) (Kumar *et al.*, 2022 ▸). There are four molecules of pTolOV in the unit cell, with an asymmetric unit of one molecule. The unit cell of the ambient phase is shown in Fig. 2 ▸(*a*). A herringbone-like configuration arises in the *bc* plane, as displayed in Fig. 2 ▸(*b*), with alternating layers of this configuration in the *a*-axis direction. Within these layers pTolOV packs in a head-to-tail configuration guided by a *C*(10) chain of O⋯H interactions between molecules in the same *bc* plane [see Fig. 2 ▸(*c*)]. Molecules in adjacent chains (*i.e.* different coloured chains in Fig. 2 ▸(*b*) interact through short H⋯H interactions. The unit cell contains a pair of molecules lying in an alternating stacked planar configuration parallel to (002), interacting through π⋯π interactions between the verdazyl radical centre of one molecule and the phenyl moiety of the adjacent molecule. Changes in the *C*(10) hydrogen bond chain and planar stacking due to applied pressure will be discussed further below.

**Figure 3:**
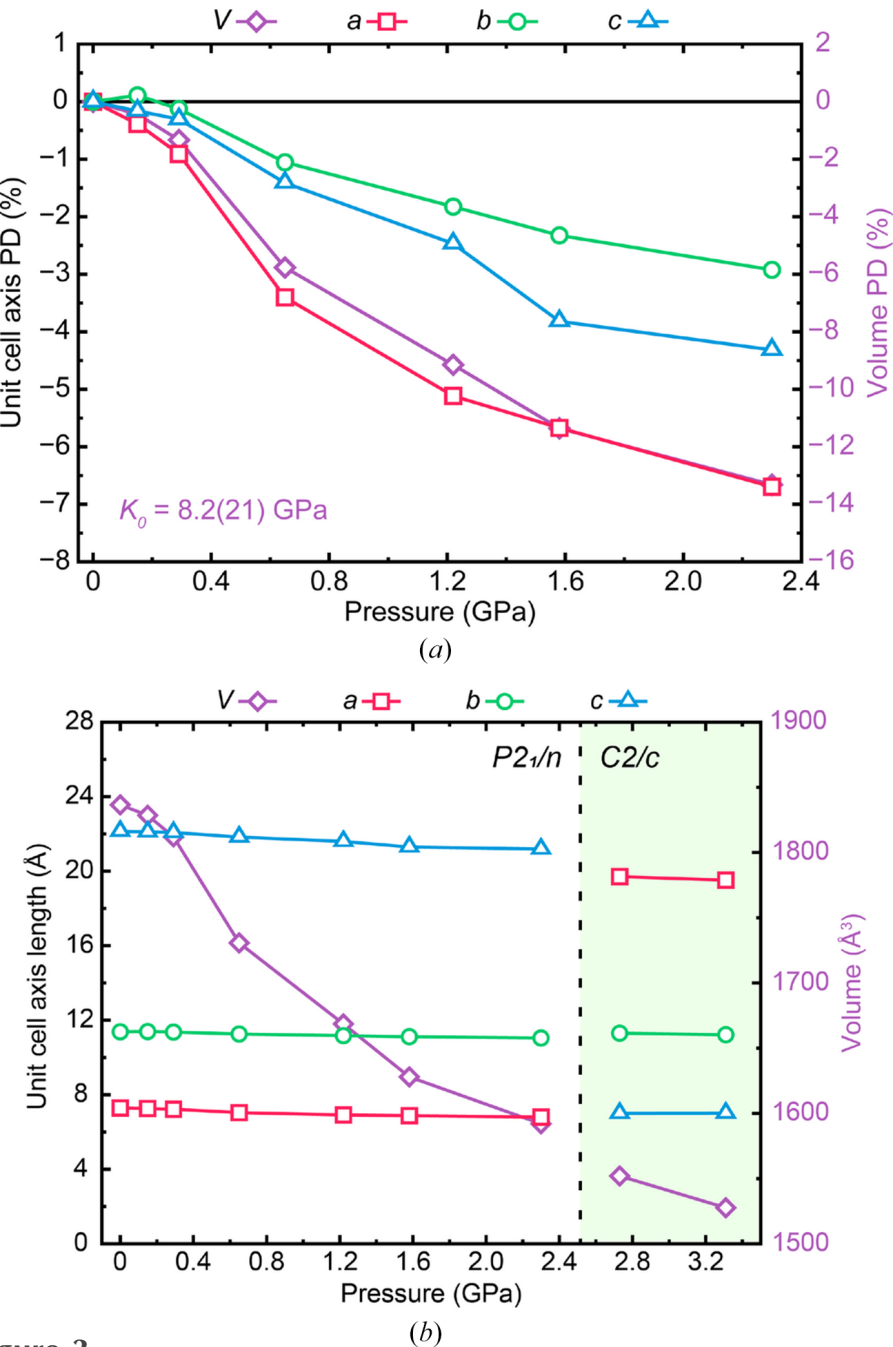
Changes in unit-cell parameters of pTolOV up to (*a*) 2.30 GPa and (*b*) 2.73 GPa (PD refers to percentage deviation).

Upon an applied hydrostatic pressure up to 2.30 GPa, the unit cell of pTolOV undergoes anisotropic distortion. The *a*, *b* and *c* axes decrease by 6.7%, 2.9% and 4.3%, respectively [Fig. 3 ▸(*a*)]. The unit-cell parameters show a consistent, even compression across the measured pressure range, following an initial period (ambient to 0.29 GPa) during which the *a*, *b* and *c* axes (and volume), decreased by less than 1.3%. An initial small increase in the *b* axis is observed at 0.14 GPa. Consistency in the standard deviation from ambient pressure to 0.14 GPa and beyond indicates that this increase is not an anomaly in data quality, but is instead a real effect. Nevertheless, such a small difference in unit-cell length is difficult to assign causation with HP data. By 2.30 GPa, the unit-cell volume undergoes a 13.3% decrease to 1592 (9) Å^3^. The β angle decreases from 90.222 (2)° at ambient conditions to 89.19 (13)° at 2.30 GPa. The crystals of pTolOV are moderately compressible, with a *K*_0_ of 8.2 (21) GPa, determined through *EosFit7* (Gonzalez-Platas *et al.*, 2016 ▸) fitting to a third-order Birch–Murnaghan equation of state (Birch, 1947 ▸) (see Section S5 of the supporting information for fitting details), consistent with the bulk modulus expected for a soft molecular solid.

Anisotropic distortion of the unit cell can be rationalized though analysis of the intermolecular contacts in each direction. The most compressible *a* axis is due to the presence of head-to-tail antiparallel planar π⋯π interactions between the herringbone-like layers in this direction. Although such interactions involving radical centres are often important for the magnetic and electrical properties of open-shell solids, it is possible that this interaction is weakened due to a lack of SOMO overlap between stacked pTolOV molecules, that would occur if the molecules were stacked in a parallel fashion. The far less compressible *b* and *c* axes contain stronger noncovalent interactions parallel to the plane of compression leading to a resistivity to pressure. The *C*(10) hydrogen bond chain is parallel to the least compressible *b* axis [Fig. 2 ▸(*c*)], highlighting the importance and structure directing nature of hydrogen-bond interactions. The *c* axis contains both H⋯H interactions, and steric repulsion arising from short tolyl–phenyl and tolyl–tolyl contacts. Changes in the tolyl–tolyl interaction will be discussed in detail further in later sections. Differences in compressibility is reflected in the linear modulus of each axis, 9.4 (39) GPa, 49.9 (130) GPa and 43.1 (101) GPa for the *a*, *b* and *c* axes, respectively (third-order Birch–Murnaghan equation of state (Birch, 1947 ▸), using *EosFit7* (Gonzalez-Platas *et al.*, 2016 ▸).

**Figure 4:**
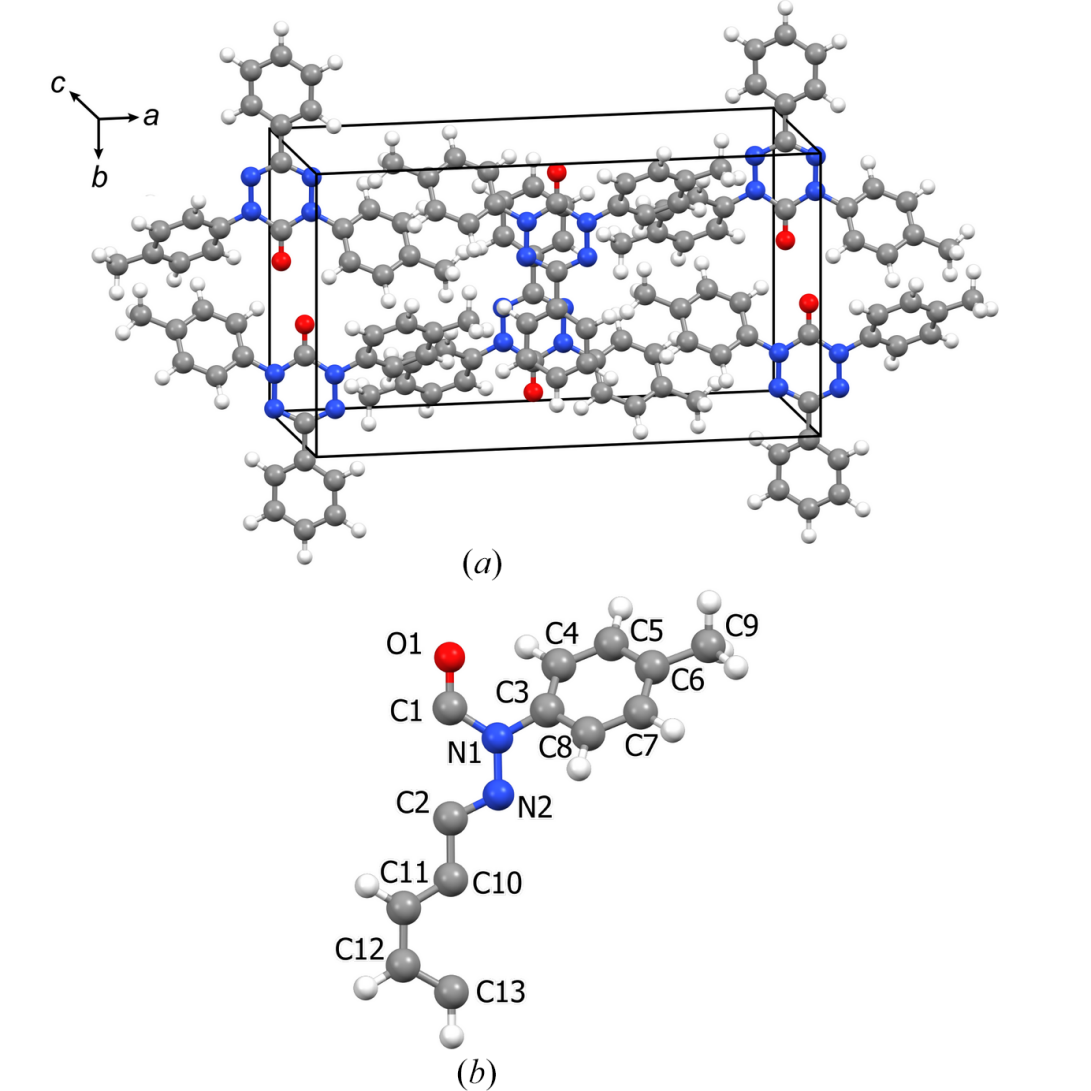
(*a*) The unit cell of the HP phase of pTolOV. (*b*) The asymmetric unit of HP pTolOV.

Between 2.30 and 2.73 GPa, pTolOV undergoes a phase transition from the primitive monoclinic *P*2_1_/*n* space group in the low-pressure (LP) phase to the centred monoclinic space group *C*2/*c* in the HP phase, an uncommon subgroup-to-supergroup transition. At 2.73 GPa, the unit cell of pTolOV has dimensions *a* = 19.71 (7) Å, *b* = 11.302 (3) Å, *c* = 7.002 (4) Å with β = 95.88 (14)° and a volume of 1552 (6) Å^3^ [Fig. 4 ▸(*a*)]. Four molecules in the unit cell are maintained following the phase transition, whilst the asymmetric unit reduces to 0.5 pTolOV molecules [Fig. 4 ▸(*b*)]. The transformation from the primitive *P*2_1_/*n* cell to the conventional *C*2/*c* setting involves the relationship *a*′ = *c*, *b*′ = *b*, and *c*′ = −*a*; full details of this transformation are provided in Section S6 of the supporting information The changes in unit-cell dimensions are summarized in Fig. 3 ▸(*b*), tabulated in Table 1 ▸, and discussed further below. Differences between the two crystal structures will be discussed henceforth. As only two pressure points of the HP phase were obtained, equations-of-state are unable to be fitted reliably. Nonetheless, compressibility appears to remain consistent beyond the point of the structural phase transition.

#### 3.1.2. Increase in symmetry: a subgroup-to-supergroup transition

**Figure 5:**
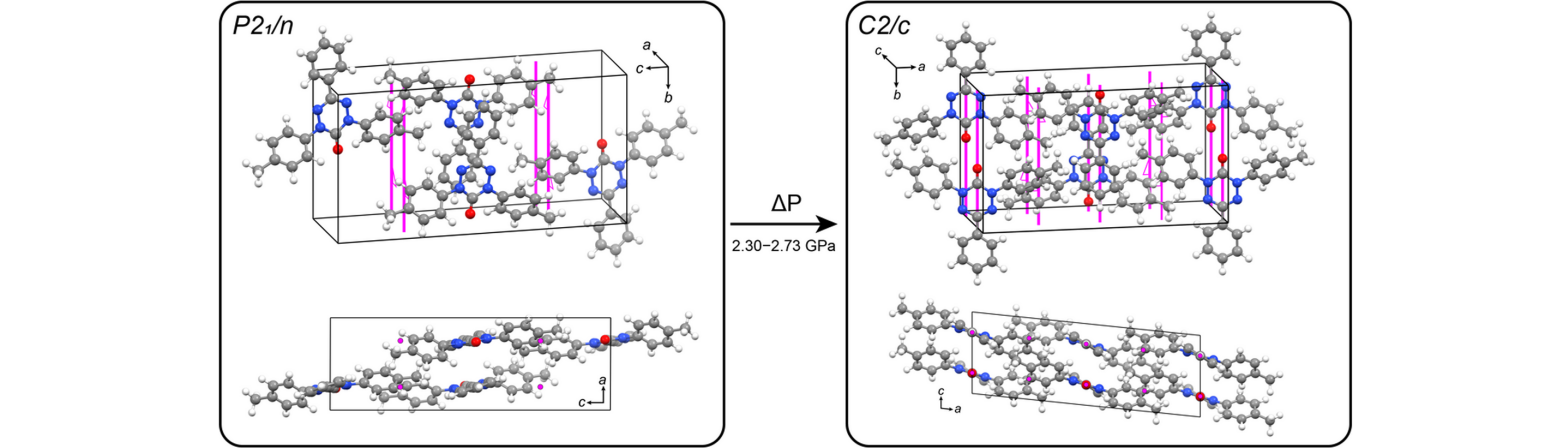
Changes in symmetry between LP and HP phases of pTolOV. Twofold screw axes are shown in pink.

The increase in symmetry from *P*2_1_/*n* to *C*2/*c* in pTolOV is visualized in Fig. 5 ▸. In the crystallographic bulk structure, it is clear that there are no significant changes in the packing and intramolecular conformation of pTolOV. The change occurs through subtle rearranging of the molecules within the unit cell, as the ‘centre’ of the pTolOV molecules (O1, C1, C2, C17, C20 using LP atom numbering) no longer lies in the general position in the LP phase (all atoms in the LP phase are in general positions), and instead lie on the special position of a proper twofold axis. These atoms in the asymmetric unit that lie on these special positions in HP pTolOV have coordinates (0, *y*, 0.75), and intersect the *ac* and *bc* planes. In the *P*2_1_/*n* LP phase, the twofold screw axes do not intersect pTolOV, providing the four molecules in the unit cell while maintaining the single pTolOV molecule as the asymmetric unit. In contrast, for the *C*2/*c* HP phase, all four molecules within the unit cell lie on a twofold screw axis. As a result, there is no change in crystallographic point group symmetry of the average crystal structure, both phases being 2/*m* monoclinic, but with an increase in molecular point group symmetry, arising from the more constrained molecular geometry caused by the increase in pressure.

Such subgroup-to-supergroup phase transitions and an increase in symmetry in response to pressure are uncommon, but not unknown. Hexaphenylbenzene for instance, undergoes a second-order non-centrosymmetric to centrosymmetric (symmetry forbidden) phase transition at 1.05 GPa, from orthorhombic *Pna*2_1_ to monoclinic *P*2_1_*/c* via an unobserved intermediate phase (Turner *et al.*, 2024 ▸). In contrast, pTolOV involves the direct transition between a subgroup (*P*2_1_/*n*) to one if its supergroups (*C*2/*c*) due to an increase in molecular symmetry. Such a phase transition is rare amongst molecular solids, however oxo-verdazyl radicals in the *C*2/*c* space group are not unprecedented, the structurally similar 1,3,5-triphenyl-6-oxo-verdazyl radical (Neugebauer *et al.*, 1993 ▸) and 2,4-bis(4-methoxyphenyl)-6-phenyl-3-oxo-verdazyl radical (Eusterwiemann *et al.*, 2016 ▸) exhibit isomorphic crystal packing with *Z*′ = 0.5.

#### 3.1.3. Hydrogen-bonded chain configuration

**Figure 6:**
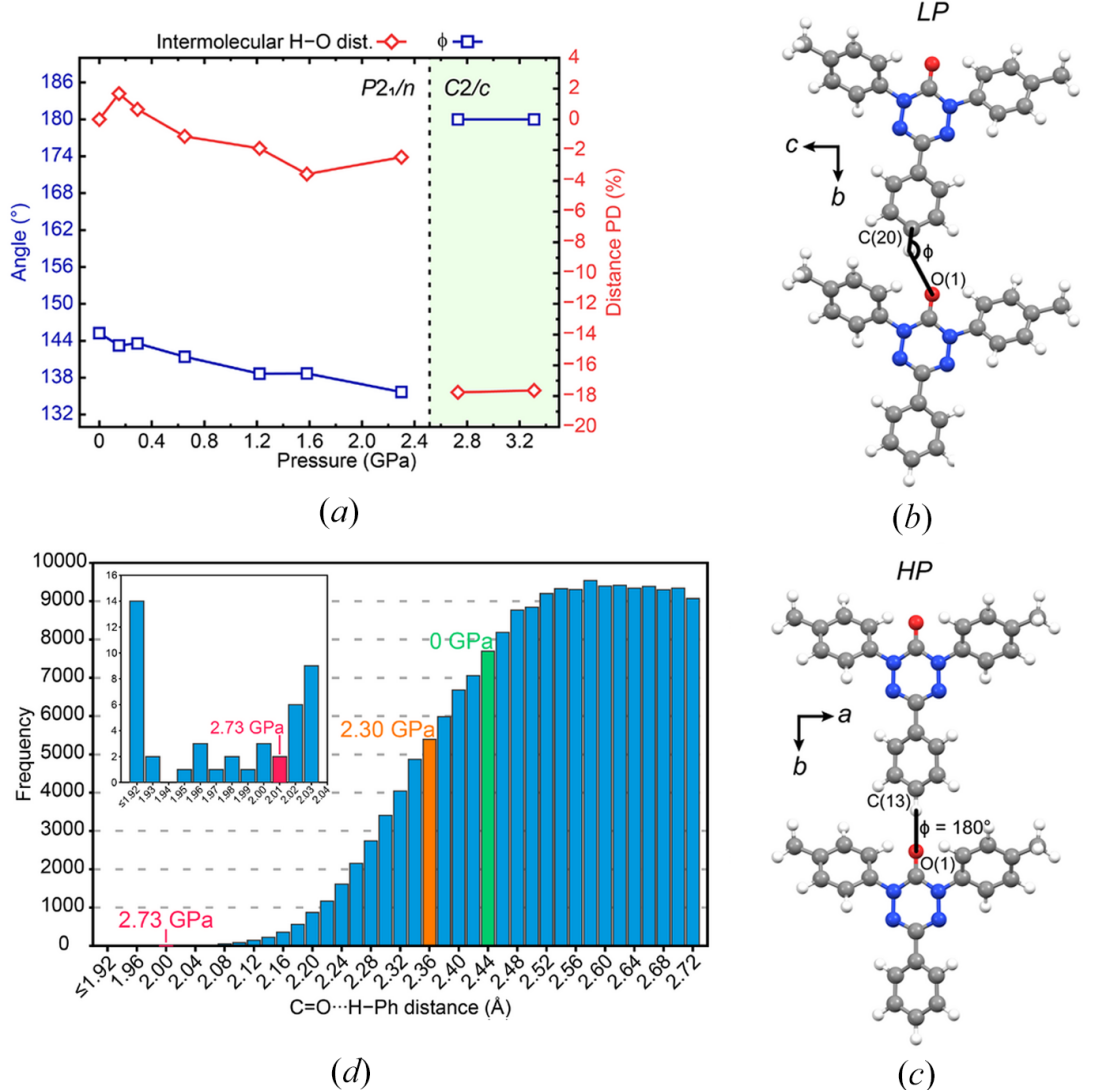
(*a*) Changes in the slip angle ϕ of the *C*(10) hydrogen bond change and percentage deviation (PD) of *d*_*C*(10)_, the intermolecular hydrogen bond distance with respect to applied hydrostatic pressure. A schematic representation of the angle and distance in the (*b*) LP (left) and HP (right) phases. (*c*) A histogram of C=O⋯H—Ph distance on the CSD (Groom *et al.*, 2016 ▸). The O⋯H distance of LP pTolOV at ambient pressure and 2.30 GPa, and HP pTolOV at 2.73 GPa are highlighted in green, orange and red, respectively.

The reduction in molecular volume and subsequent increase in molecular symmetry provides the main driving force behind the structural changes in pTolOV observed between the LP and HP phases, which can be explored through the features of the *C*(10) hydrogen bond chain in the *b*-axis direction. The angle ϕ is defined [Figs. 6 ▸(*a*) and 6 ▸(*b*), and summarized in Table S1 of the supporting information] as the angle through the C—H of the phenyl group (*para* to the verdazyl core) of one pTolOV molecule to the oxygen of the adjacent molecule below. This angle can be thought of as the slip angle between adjacent molecules in the *C*(10) hydrogen bond chain. During compression, the intermolecular hydrogen bond distance decreases minimally prior to the phase transition, whilst ϕ undergoes a steady reduction, of up to 8.5% up in the (LP) 2.30 GPa phase. There are more significant changes in both ϕ and the intermolecular hydrogen bond distance between the LP and HP phases. The intermolecular distance (*d*_*C*(10)_) decreases from 2.36 Å to 2.02 Å (a 14.5% decrease) between 2.30 and 2.73 GPa, whilst ϕ increases from 132.96° to 180° between 2.30 and 2.73 GPa. This increase in ϕ to 180° is reflective of the increase in molecular point group symmetry.

Fig. 6 ▸(*c*) displays the frequency of C=O⋯H—Ph intermolecular contacts in the Cambridge Structural Database (CSD) (Groom *et al.*, 2016 ▸), up to the sum of the oxygen and hydrogen van der Waals radii (2.72 Å). To visualize the changes in pTolOV O⋯H distances with respect to the data in the CSD (Groom *et al.*, 2016 ▸), the hydrogen bond distances in the LP phase of pTolOV at ambient pressure and 2.30 GPa are highlighted in green and orange, respectively, and in red for the HP phase at 2.73 GPa. The O⋯H distances in LP pTolOV lie below of the modal average of 2.56–2.58 Å. At 2.73 GPa, there is a dramatic drop in contact distance, with only 29 structures containing hydrogen-bond distances less than or equal to 2.01 Å (*i.e.* 0.016% of the 183604 contacts below 2.72 Å) [Fig. 6 ▸(*c*) inset]. The shorter O⋯H interaction distance likely acts as a stabilizing interaction in the HP phase.

The presence of hydrogen bonds in the solid state, due to their relative strength compared to other intermolecular interactions, are important and can be structure directing interactions in organic molecular crystals. Despite the classical definition of hydrogen bond donors involving more electronegative elements such as nitrogen, oxygen and fluorine, carbon can act as a weak source of electron density (Desiraju, 1991 ▸). While the C—H⋯*X* angle can range between 90° and 180°, angles approaching 180° are considered stronger (Steiner, 2002 ▸). The combination of an increased angle of ϕ to 180° and a decreased *d*_*C*(10)_ distance to 2.02 Å indicates that the strength and attractive nature of this interaction plays a role in the phase transition and stabilizing of the HP phase.

#### 3.1.4. Changes in inter/intramolecular conformation

**Figure 7:**
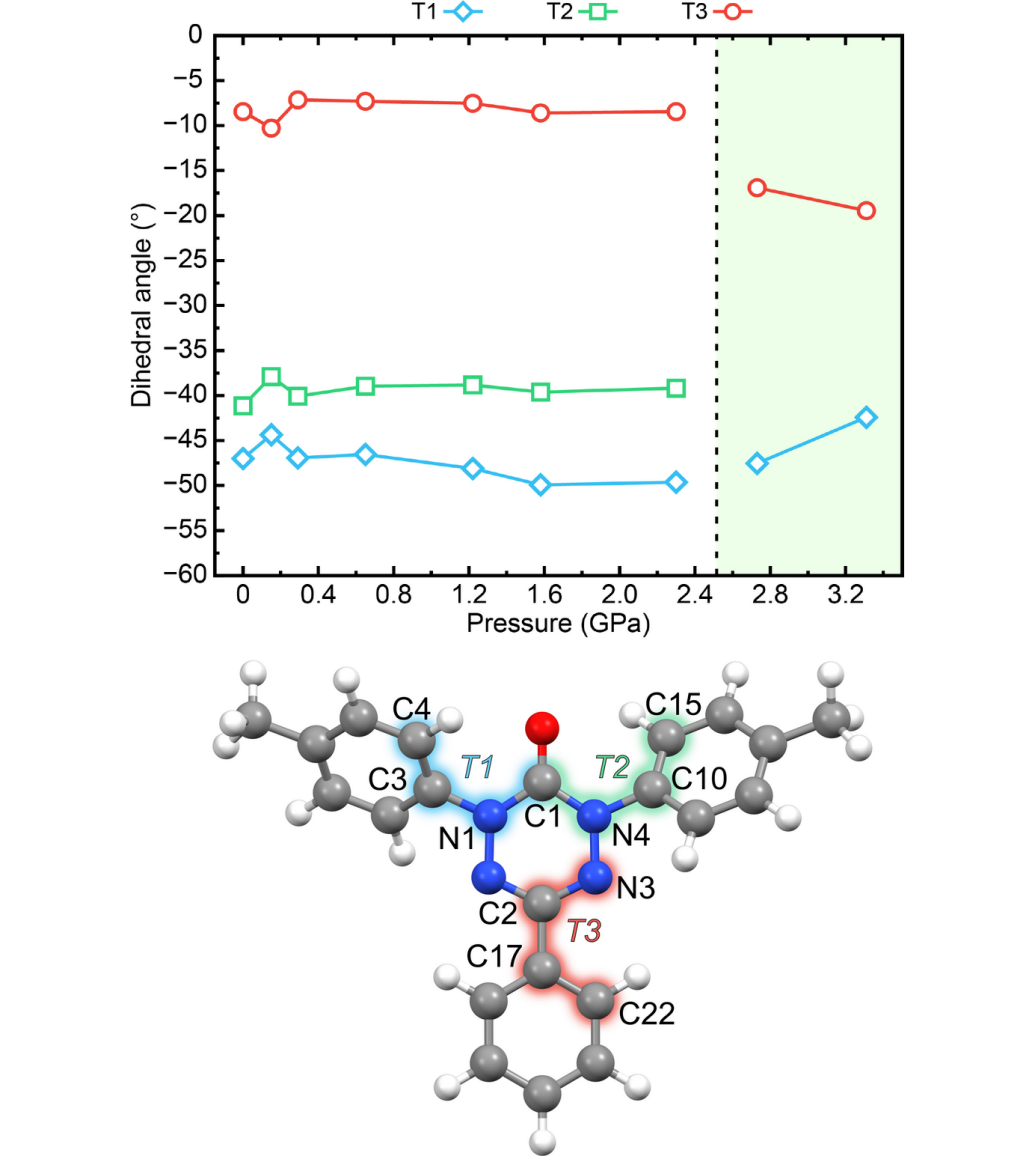
Changes in torsion angles T1, T2 and T3 with respect to an applied hydrostatic pressure (top), the definition of T1, T2 and T3 in pTolOV, atom numbering is consistent with the LP phase (bottom). Note: T1 and T2 are equal in the HP phase, hence the removal of T2 from the above plot from 2.73 to 3.31 GPa

Three key intramolecular torsion angles in pTolOV allow mapping of changes in the inter- and intramolecular conformations in the two phases (Fig. 7 ▸, Table S1 of the supporting information). Using atom numbering from the LP *P*2_1_/*n* phase, T1 is defined as the torsion angle through atoms C4,C3,N1,C1, T2 through C15,C10,N4,N3, and T3 through N3,C2,C17,C22. Due to the increased symmetry in the HP *C*2/*c* phase, T1 and T2 become equivalent. Torsion angles are measured using the *PLATON* software package. (Spek, 2003 ▸) At ambient pressure, T1, T2 and T3 are −47.00 (18)°, −41.15 (19)° and −8.45 (19)°, respectively. An increase in torsion angle towards zero is indicative of the tolyl moieties rotating towards the plane of the verdazyl centre, whilst a decrease towards −90° indicates a perpendicular configuration with respect to the verdazyl centre. Upon compression to 2.30 GPa, T1 decreases to −49.6 (12)°, whilst T2 increases to −39.1 (11)°, indicating each tolyl ring is rotating independently, with the C3–C8 becoming more perpendicular, whilst the C10–C15 phenyl ring becomes more planar to the verdazyl ring. T3 decreases marginally from −8.45 (19)° to −8.5 (12)° at 2.30 GPa, the phenyl ring changing very little due to compression. Upon the transition to the HP phase, T1 becomes −47.5 (16)°, and angle much closer to that of the ambient structure and a decrease from that at 2.30 GPa. As mentioned, due to symmetry T2 becomes equivalent to T1, and therefore decreases from −39.1 (11)° at 2.30 GPa to −47.5 (16)° at 2.73 GPa. T3 undergoes a significant decrease from −8.5 (12)° to −16.9 (10)°, indicating the C17–C22 ring re-orients to a conformation somewhat more out of the plane of the verdazyl ring.

A gas-phase DFT (ROHF DSD-PBEP86-D3BJ/def2-SVP) torsional rotation of the tolyl moiety from the ambient geometry to the 3.31 GPa angles (see Section S4 of the supporting information for expanded computational details), reveals a barrier to rotation of less than 1 kJ mol^−1^, well within chemical accuracy. This indicates that the change in torsion angle is not a significant driving force in this phase transition, rather it is a means to reduce the steric hindrance as pressure increases. Rotation of the tolyl moieties brings about a steady increase in the C3–C8⋯C10–C15 centroid–centroid distance (phenyl rings from adjacent tolyl groups) from 4.93 Å at ambient pressure to 5.95 Å at 2.73 GPa. Rotation of the T3 angle to −17° brings the shortest C–N distance between verdazyl and phenyl groups to within the sum of van der Waals radii. At 0 GPa, the shortest C–N distance within the planar unit-cell dimer is 3.477 (2) Å (N3–C18), at 2.73 GPa this distance is reduced to 3.19 (2) Å (N2–C11), below the combined nitrogen and carbon van der Waals radii of ∼3.25 Å. Indicating that the phase transition may have been driven by strong π*–π interactions between the SOMO of the verdazyl group and π orbitals from the above phenyl ring.

**Figure 8:**
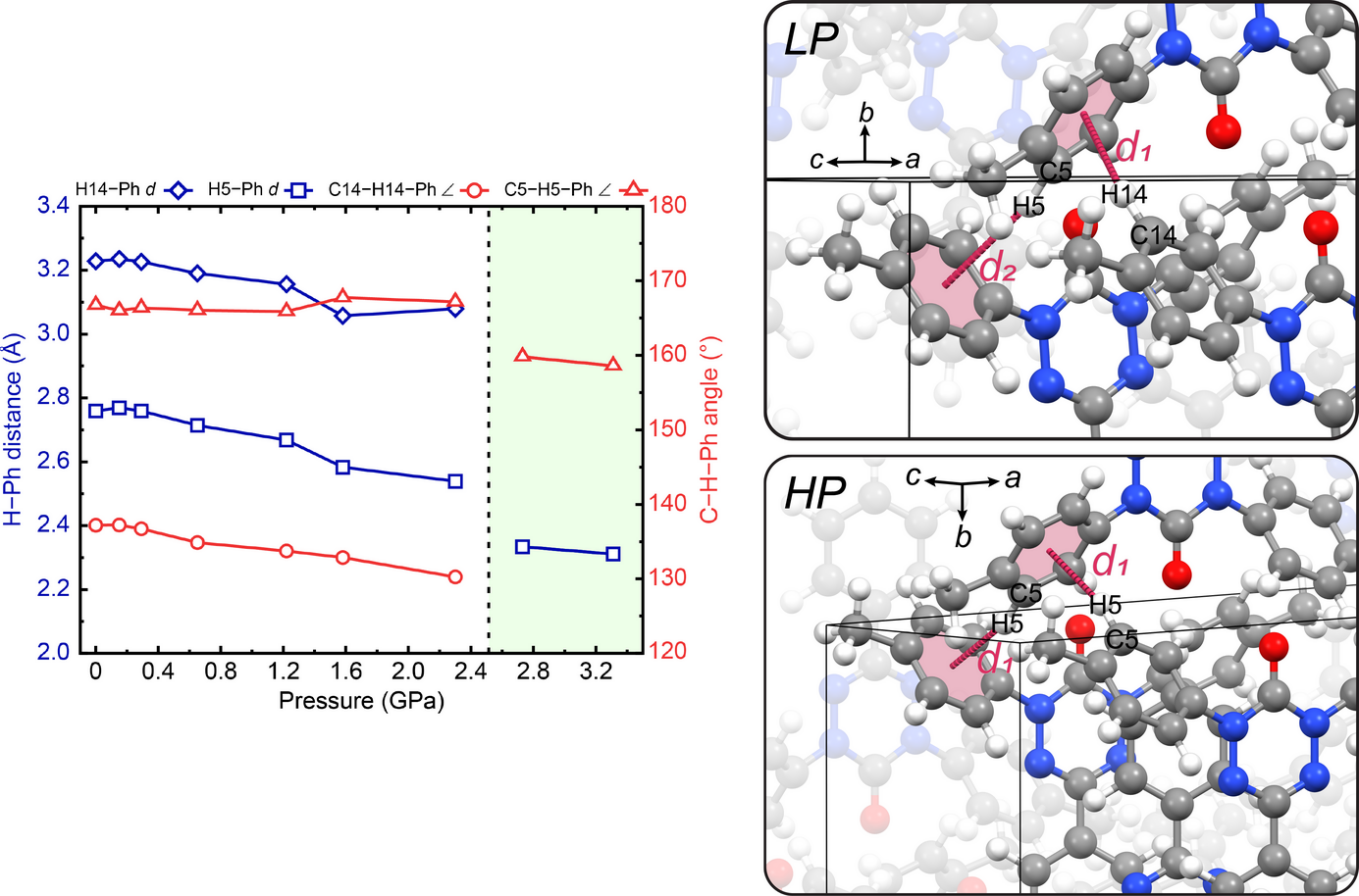
Changes in intermolecular CH⋯π interaction distances, *d*_CHπ1_ and *d*_CHπ2_ and angles ∠_1_ and ∠_2_ (left), and their respective positions within the LP (top right) and HP (bottom right) phases of pTolOV.

Neighbouring molecules in the unit cell of pTolOV exhibit C—H⋯π interactions. In the LP phase of pTolOV, *d*_CHπ1_ is defined as the C—H⋯π distance of the interaction between C14—H14 and the centroid of the C3–C8 ring of the neighbouring molecule at position (−*x* + 1, −*y* − 1, −*z*), with *d*_CHπ2_ defined as the distance of the interaction between C5—H5 and the centroid of the C10–C15 ring of the neighbouring molecule at position (−*x*, −*y* − 1, −*z*). Due to the increased symmetry in HP pTolOV, *d*_CHπ1_ and *d*_CHπ2_ become equivalent, and represent the distance of the interaction between C5—H5 and the centroid of ring C3–C8 of the molecule at position (*x* + 1/2, −*y* + 1, *z* + 1/2). The location and geometry of these C—H⋯π interactions within the unit cell, changes in the H⋯centroid distance and C—H⋯centroid angle with applied pressure are shown in Fig. 8 ▸. Here, ∠_1_ and ∠_2_ are defined as the C—H⋯centroid angles associated with *d*_CHπ1_ and *d*_CHπ2_, respectively. At ambient pressure, *d*_CHπ1_ and *d*_CHπ2_ are 3.23 Å and 2.76 Å, and ∠_1_ and ∠_2_ angles 137.19° and 166.74°, respectively. Based on the angle and distance and the increase in linearity of hydrogen bonded interactions, it can be assumed that *d*_CHπ2_ is the stronger of the two interactions under ambient conditions.

Upon compression to 2.30 GPa, both *d*_CHπ1_ and *d*_CHπ2_ decrease in LP pTolOV, to 3.06 Å and 2.55 Å respectively (Δ*d*_CHπ1_ = −5.36%, Δ*d*_CHπ2_ = −7.65%). The angle ∠_1_ sees a much larger change than ∠_2_ up to this pressure point in the data set, with ∠_1_ decreasing to 131.45°, whilst ∠_2_ decreases by only 0.07°to 166.67°. The decrease in ∠_1_ is a product of the C10–C15 phenyl ring becoming more planar (with respect to the verdazyl core) through compression, serving to decrease the strength and directionality of the hydrogen bonded interaction. Through the transition from the LP to HP phases and the merging of *d*_CHπ1_ and *d*_CHπ2_, the critical C—H⋯π distance *d*_CHπ1_ in the HP phase becomes 2.34 Å, representing a decrease from the 2.30 GPa structure of −23.40% for *d*_CHπ1_ and −8.16% for *d*_CHπ2_. Commensurate changes in ∠_1_ and ∠_2_ are apparent, with these angles becoming a common value of 160.92° at 2.73 GPa (merging similarly to *d*_CHπ1_ and *d*_CHπ2_ in response to the change in molecular point group symmetry). This increase in ∠_1_ of 29.47° strengthens the C—H⋯π interaction. Changes in *d*_CHπ1_, *d*_CHπ2_, ∠_1_ and ∠_2_ with respect to pressure are summarized in Table S1 of the supporting information.

#### 3.1.5. Planar π–π interactions

**Figure 9:**
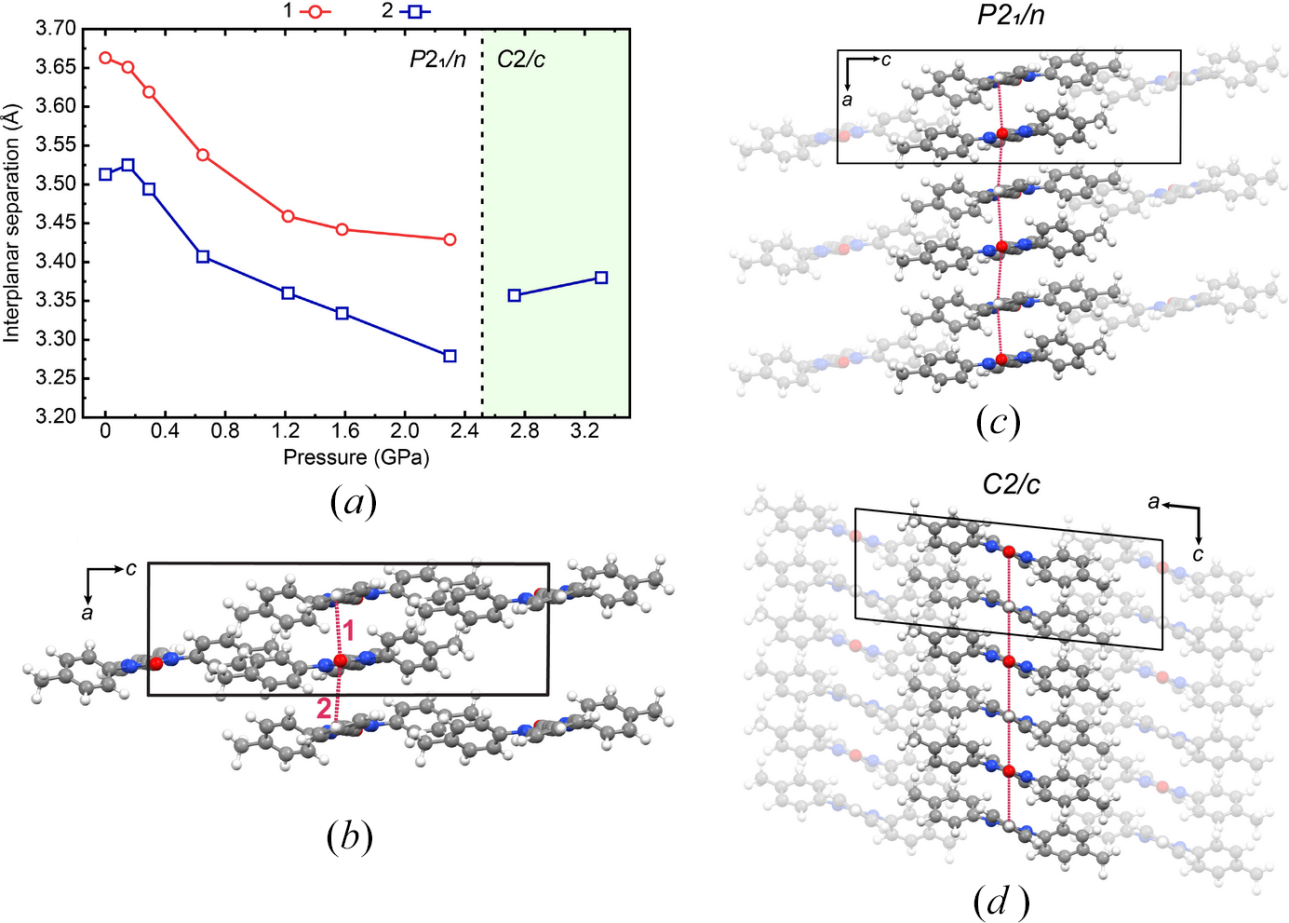
(*a*) Change in distance between the mean planes of verdazyl rings of adjacent planar stacked molecules, *d*_p1_ and *d*_p2_ and (*b*) their positions within the pTolOV structure. (*c*) Zigzag alternating π⋯π chains in LP pTolOV. (*d*) Slanted regular π⋯π chains in HP pTolOV.

In the crystalline phase, pTolOV molecules pack through head-over-tail (antiparallel) planar π⋯π interactions in the centre of the unit cell, forming chains along the *a* axis in the LP phase, and the *c* axis in the HP phase, with the verdazyl radical centre lying above the phenyl ring of the adjacent molecule. In the LP phase, there are alternating distances between molecules which are offset forming a zigzag pattern [Fig. 9 ▸(*c*)]. In the HP phase, the alternating distances merge, and the molecules stack at regular intervals, indicative of the increase in molecular point group symmetry. Distances between these dimers [Figs. 9 ▸(*a*), 9 ▸(*b*)], *d*_p1_ and *d*_p2_, are defined as the distance between the mean planes of verdazyl rings of adjacent planar stacked molecules, and are summarized in Table S1 of the supporting information. The angle between the mean planes of adjacent verdazyl and phenyl rings is also discussed below, and do not alternate in the same manner as *d*_p1_ and *d*_p2_.

Under ambient conditions, *d*_p1_ and *d*_p2_ are 3.66 and 3.51 Å, respectively, with an angle of 8.61°. These distances are reduced through compression at 2.30 GPa [Fig. 9 ▸(*a*)] to 3.43 and 3.28 Å (Δ = −6.39/−6.66%) for *d*_p1_ and *d*_p2_, respectively. The verdazyl–phenyl plane angle increases to 9.66°, consistent with the reduction in the T3 torsion angle. The separation distance in the HP phase at 2.73 GPa changes to 3.36 Å, a 2.10% decrease for *d*_p1_ and a 2.38% increase for *d*_p2_. This change is consistent with the changes in tolyl torsion angles (T1/2), whilst changes in the phenyl torsion angle (T3) is reflected in changes to the distance between centroids of adjacent verdazyl and phenyl rings. The distance increases for both dimers between 2.30 and 2.73 GPa from 3.46 to 3.51 Å for the first dimer and from 3.39 to 3.51 Å for the second dimer. The decrease in *d*_p1_ is associated with an increase in the offset distance perpendicular to the direction of the π-stacks, which will be discussed further in relation to magnetic calculations. Planar π⋯π chains in the ambient structure of the LP phase zigzag at an angle of 170.42° [Fig. 9 ▸(*b*)], that become regular (not alternating) and more linear and slanted (due to the offset distance) in HP pTolOV with an angle of 173.15°, due to the repeating π⋯π dimers lying along (200) [Fig. 9 ▸(*d*)].

Similar antiparallel planar stacking is relatively uncommon within oxo-verdazyl radicals, featuring in 12 of the 77 oxo-verdazyl radicals on the CSD (Groom *et al.*, 2016 ▸) (16%)^1^. One such example is the 1,5-dimethyl-3-(2-pyridyl)-6-oxo-verdazyl radical, which forms head-to-tail dimers separated by 3.37 Å and 3.44 Å in its cocrystal with hydroquinone (Hicks *et al.*, 2001 ▸). As mentioned, planar interactions are often the mode through which magnetic interactions can occur in verdazyl radicals. Of the 12 oxo-verdazyl radical compounds on the CSD (Groom *et al.*, 2016 ▸) with antiparallel π-stacking, six have associated magnetic susceptibility data (Hicks *et al.*, 2001 ▸; Plater *et al.*, 2006 ▸; Norel *et al.*, 2008 ▸; Norel *et al.*, 2010 ▸; Kumar *et al.*, 2018 ▸). The strength of magnetic exchange coupling of these compounds ranges in from −1.07 (1) cm^−1^ for the 1,5-dimethyl-3-(4-acetomidophenyl)-6-oxo-verdazyl radical (Plater *et al.*, 2006 ▸), to −113 cm^−1^ for the 1,5-dimethyl-3-(2′-hydroxyphenyl)-6-oxo-verdazyl radical (Norel *et al.*, 2010 ▸). There is minimal correlation between the interplanar separation or verdazyl–verdazyl centroid distances and the magnetic coupling, although it is influential to an extent, as the largest radical centre separation is associated with the weakest magnetic interaction [the 1,5-dimethyl-3-(4-acetomidophenyl)-6-oxo-verdazyl]. Magnetic coupling in organic radical crystals that lack direct SOMO–SOMO overlap is an atypical concept (Hicks *et al.*, 2001 ▸), highlighting the sensitivity of magnetic interactions to subtle effects influenced by solid state packing (Norel *et al.*, 2010 ▸; Norel *et al.*, 2011 ▸).

If the goal of synthesizing new organic radical compounds is to produce magnetic interactions, consideration of the distance between radical centres in the crystal structure is crucial. It is difficult to produce stable radical crystals with the desired magnetic topology, such is its sensitivity to packing (Luzón *et al.*, 2003 ▸), Careful balance must be struck between the stability of the resulting crystal structure and the close radical centre contacts, as they can be considered somewhat mutually exclusive. In verdazyl radicals, crystal structures can be weighted towards certain packing motifs by altering the atomic weight of the 1-, 3- and 5-position substituents, although it is still difficult to predict with certainty. We discuss potential magnetic interactions in pTolOV in a later section, as such interactions are a key facet of producing cutting-edge organic electronic components.

### 3.2. Periodic DFT

**Figure 10:**
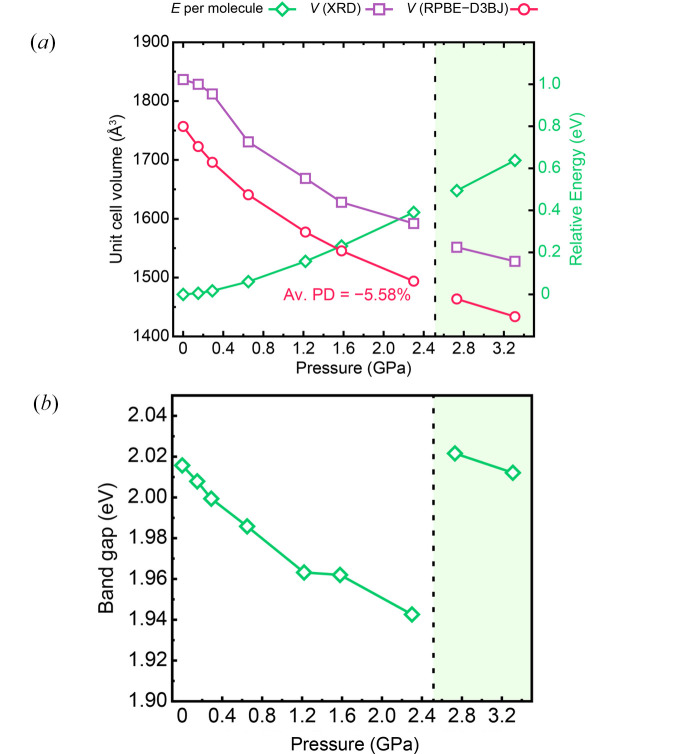
(*a*) Potential energy per pTolOV molecule, experimental XRD and RPBE-D3BJ optimized unit-cell volumes of pTolOV, with varying pressure. Note: the volume of the two HP phase points (in the green shaded area) have been doubled to match the experimental *C*2/*c* unit cell, instead of the primitive cell that was used during the optimization process. (*b*) The Γ-centred band gap of pTolOV with varying pressure.

To investigate changes in electronic structure of pTolOV with pressure, the experimental structures obtained via XRD must first be optimized. The difference between experimental structures and RPBE-D3BJ optimized structures and potential energy per molecule are displayed in Fig. 10 ▸(*a*). To increase the efficiency of calculations where possible, the HP phase of pTolOV was optimized as its primitive cell, halving the unit-cell volume. The unit-cell parameters of the primitive cell of the HP pressure points are displayed in Table 2 ▸. At ambient pressure, the RPBE-D3BJ optimized structure has a percentage deviation of −4.34% compared to experiment. At 2.30 GPa and 2.73 GPa, the volume percentage deviation is −6.16% and −5.66%, respectively. The average volume percentage deviation is −5.58% across the pressure series. This relatively small and consistent deviation from experimental unit-cell volumes highlights the efficacy of the *VASP* PSTRESS tag, used to apply an external pressure during calculations, and gives confidence in the use of these optimized structures for further electronic structure calculations. Optimized unit-cell parameters and volume are summarized in Table 2 ▸.

The energy per molecule decreases by less than 1 eV during compression to 3.31 GPa. This result indicates that the subtle rearrangement of the molecules from the LP to HP phases does not require a large energy penalty, and the *C*2/*c* structures are not under a significant amount of strain, highlighting the subtlety of this phase transition and relative stability of the *C*2/*c* structures compared to the *P*2_1_/*n* structures. The magnetic configuration was assumed to be antiferromagnetic (AFM) across each pressure point, based on precedence from previous literature studies on verdazyl radicals. To investigate the differences in magnetic configuration, a single-point energy calculation was completed with a ferromagnetic (FM) configuration (any −1 magnetic moments in MAGMOM command in *VASP* were changed to + 1, giving a net magnetic moment of 4 across the unit cell). As expected, the FM configuration was less stable than the AFM state, the difference in stability increased with increasing pressure in the LP phase, increasing from 10.8 meV per unit cell at ambient pressure to 28.2 meV at 2.30 GPa; however both energies are below the assumed accuracy of computational methods. In the HP phase, the energy difference was lower than the LP phase, at 3.0–3.2 meV. This change is reflective of a change in the bulk electronic properties, likely prompted by changes in radical SOMO proximity.

Due to the large number of atoms per unit cell, calculating the band structure of pTolOV with an appropriate hybrid functional such as HSE06 carries a significant computational cost. For the following discussion on the relationship between the band gap of pTolOV and applied pressure, data from a single-point energy calculation sampling only the Γ-point of each pressure point was employed. These calculations act as a proxy for the band gap, with an associated assumption that the valence band maximum and conduction band minimum both occur at the Γ-point. For the DOS calculations following, the *k*-point mesh is extended to a larger grid, calculated using the automatic *k*-point mesh generation method in *VASP*, with a *R*_*k*_ value of 20.

The HSE06 calculated band gap at the Γ-point of the Brillouin zone is plotted against applied hydrostatic pressure in Fig. 10 ▸(*b*), and summarized in Table 3 ▸, illustrating a reduction in band gap from 2.016 eV at ambient pressure to 1.943 eV at 2.30 GPa. Upon the transition to the HP phase, the band gap of pTolOV increases marginally to 2.022 eV, above the ambient value. The decrease in band gap for the LP phase is consistent with a reduction in the antiparallel planar dimer distance (and therefore SOMO distance), as illustrated in Fig. 9 ▸. The increase in band gap post-transition is seemingly at odds with the continued and discontinuous planar dimer distance decrease, but arises from a shift in the relative positions of adjacent molecular parallel to the verdazyl ring. This shift in relative position has repercussions for magnetic interactions, and will be discussed in a later section. The changes in band gap are minimal in both phases, and pTolOV remains on the cusp between a wide-gap semiconductor and insulator. For reference, a band gap of 3.92 eV was measured for the Kuhn radical 3-(4-iodophenyl)-1,5-diphenyl-verdazyl radical, with verdazyl–verdazyl centroid distances of 5.02 Å (Jobelius *et al.*, 2018 ▸).

**Figure 11:**
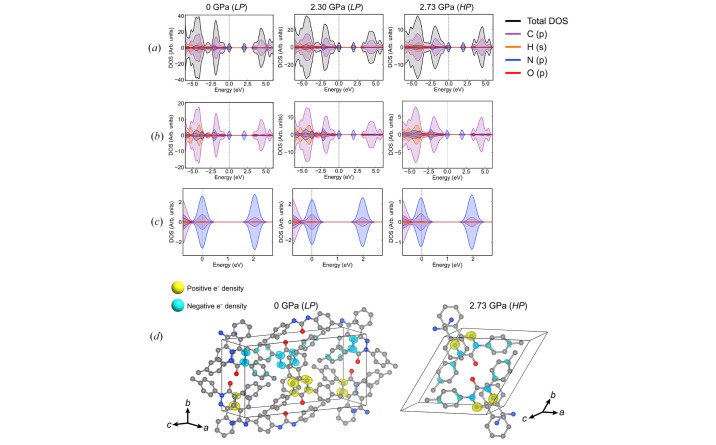
Density of states of pTolOV at 0, 2.30 and 2.73 GPa calculated at the Γ-point. Energy here has been scaled such that the valence band maximum (VBM) is at 0 eV. Only contributors to the DOS over 3% are shown in legend. Negative DOS refers to the spin-down channel, positive refers to spin-up. (*a*) Total DOS, (*b*) DOS with total excluded, (*c*) DOS around the band gap. (*d*) HSE06 calculated charge density difference between conductance band and valence band in pTolOV at 0 and 2.73 GPa. Isosurface value = 0.002.

The DOS plots (Fig. 11 ▸) assist in the elucidation of information about the orbital contributions to the electronic structure, which is important in the engineering of new organic radical solids for molecular electronic applications. There is a reduction in the total number of states with the application of pressure in the valence and conductance orbitals outside of the SOMO [Fig. 11 ▸(*a*)]. It should be noted that as an antiferromagnetic configuration (no net magnetic moment) was assumed for these calculations, there is no difference between the spin-up (positive DOS) and spin-down (negative DOS) channels. The states around the band gap are primarily nitrogen *p* orbitals of the verdazyl SOMO [Fig. 11 ▸(*c*)], and at both pressures, there is less electron density on the tolyl groups in the conductance band than the valence band. Charge density differences between the conductance band and valence band were calculated using the *VASPKIT* (Wang *et al.*, 2021 ▸) software package, and plotted with *VESTA* (Momma & Izumi, 2011 ▸). The resulting density plots are shown in Fig. 11 ▸(*d*), and highlight that the process associated with the band gap of pTolOV may involve excitations between the SOMO of adjacent molecules.

It may therefore be deduced that, perhaps unsurprisingly, designs for verdazyl radical-based molecular solids with increased conductivity and decreased band gaps should involve close contact between the radical centres, enhancing their properties. High-level multireference calculations have shown that decreasing the distance between two verdazyl centres, particularly in a parallel face-to-face configuration (with the verdazyl centres sitting parallel above each other), can increase the bandwidth and decrease the Coulomb repulsion between adjacent molecules, increasing the likelihood of a metallic ground state (Rota *et al.*, 2010 ▸). Decreasing the distance between verdazyl radicals in the solid state is a balancing act, in which maintaining crystal stability is equally as important, as stability of the open-shell nature is vulnerable, instead making way for diamagnetic dimers within the crystal structure (Fujita *et al.*, 2002 ▸).

### 3.3. Magnetic interaction calculations

Magnetic susceptibility data was not obtained for this sample of pTolOV, so to supplement discussions around the viability of pTolOV for molecular electronics applications, the response to pressure of the magnetic topology is calculated. Further supplementation is achieved through comparison to structures in the CSD (Groom *et al.*, 2016 ▸) with associated magnetic susceptibility data. Magnetic susceptibility measurements of the 1,5-dimethyl-3-(2-pyridyl)-6-oxo-verdazyl radical co-crystal with hydroquinone by Hicks *et al.*, with antiparallel head-to-tail π-stacking similar to pTolOV (Hicks *et al.*, 2001 ▸), revealed a magnetic exchange coupling parameter *J*, of −58 cm^−1^, and calculations at the BP86/DZVP level of theory found *J* = −120.5 cm^−1^. *J* was calculated as the difference between a low spin doublet and high spin quartet (2*J* = *E*^doublet^ − *E*^quartet^) of three planar stacked molecules. The trimer configuration was chosen over the more conventional dimer approach for organic radicals as there are no clear contacts between verdazyl radicals, and thus magnetic interactions were assumed to involve a through space exchange interaction between two verdazyl radical centres, mediated by a central phenyl ring.

Jornet *et al.* (2006 ▸) determined that for recreating experimental magnetic data of the same 1,5-dimethyl-3-(2-pyridyl)-6-oxo-verdazyl radical cocrystal, the gain in accuracy for the triplet model over the dimer model did not outweigh the increased computational cost associated with calculating the energy of three large molecules. Thus the more conventional dimer approach, in which the magnetic coupling is assessed as the difference between the broken-symmetry singlet and the triplet states (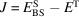) was utilized (Deumal *et al.*, 2002 ▸), allowing dimers within the triplet model to be assessed separately. This method applied using UB3LYP/6-31+G(d) calculations gave reasonable agreement with experimental data and CASSCF calculations (Jornet *et al.*, 2006 ▸). Due to alternating distances between verdazyl radicals, care must be taken to ensure that both magnetic interactions are calculated separately when using a dimer method such as this.

Using the UB3LYP/6-31+G(*d*) in the *ORCA5* software package, we are able to recreate the magnetic coupling parameter for the 1,5-dimethyl-3-(2-pyridyl)-6-oxo-verdazyl radical, *J* = −55.73 cm^−1^ and *J* = −54.08 cm^−1^ for verdazyl–pyridine distances of −3.54 Å and −3.41 Å, respectively [similar to *J* = −55.97 cm^−1^ and *J* = −54.43 cm^−1^ for the same interactions and level of theory as Jornet *et al.* (2006 ▸)]. The strength of the magnetic exchange of a further two verdazyl radicals from the CSD (Groom *et al.*, 2016 ▸) were calculated using this method and compared to experiment. The 3-(2′-imidazolyl)-1,5-dimethyl-6-oxo-verdazyl radical of Norel *et al.* (CSD code QIZBAD) has an average interplanar separation of 3.42 Å between antiparallel π-stacked molecules (Norel *et al.*, 2008 ▸), and an antiferromagnetic interaction of *J* = −100 cm^−1^. This separation measurement is the average centroid–centroid distance between the verdazyl ring and imidazole of the adjacent molecule. At the UB3LYP/6-31+G(d) level of theory, we calculate that the magnetic exchange interaction has a strength of *J* = −31.50 cm^−1^ (this value was averaged across two dimers with the same separation, but differing interactions of −31.55 cm^−1^ and −31.45 cm^−1^).

Kumar and coworker’s 1,5-dimethyl-3-(4′-carboxyphenyl)-6-oxo-verdazyl radical (CSD code YEQPIW) exhibits an antiferromagnetic interaction of *J* = −90 cm^−1^ from antiparallel π-stacking of 3.57 Å and 3.52 Å (Kumar *et al.*, 2018 ▸). The calculated magnetic exchange interactions are calculated as *J* = −49.51 cm^−1^ and *J* = −46.73 cm^−1^ for the dimers separated by 3.57 Å and 3.52 Å, respectively. Whilst these values of *J* do not replicate experimental data as well as the 1,5-dimethyl-3-(2-pyridyl)-6-oxo-verdazyl radical, we still see agreement in the sign and magnitude of the interaction, and can thus apply this method with reasonable confidence of qualitative results of the strength and magnitude of magnetic exchange interactions in pTolOV.

Alternating antiparallel dimers of the LP phase of pTolOV under ambient conditions are separated by 3.66 Å and 3.51 Å. Using the 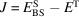 method with UB3LYP/6-31+G(d), we obtain interaction strengths of *J* = −5.16 cm^−1^ and *J* = −3.41 cm^−1^ (average *J* = −4.29 cm^−1^). At 2.73 GPa in the HP phase of pTolOV, dimers are separated evenly by a shorter distance of 3.36 Å, and a calculated magnetic interaction of *J* = −4.95/−4.75 cm^−1^. These results indicate weak antiferromagnetic interactions between dimers in both phases of pTolOV, and a possible alternating 1D antiferromagnetic chain in the *a*-axis direction in LP pTolOV and the *c*-axis direction in HP pTolOV.

Based on the difference in calculated and experimental *J* values for the 3-(2′-imidazolyl)-1,5-dimethyl-6-oxo-verdazyl radical and 1,5-dimethyl-3-(4′-carboxyphenyl)-6-oxo-verdazyl radical, it is possible that the pTolOV experimental *J* will be greater than calculated, but we are unable to confirm this without magnetic susceptibility data. The calculated pTolOV interactions are comparable to the 1,3,5-triphenyl-6-oxo-verdazyl radical, with analogous head-to-tail π-stacking and verdazyl–-phenyl interactions, for which magnetic susceptibility measurements and modelling determined *J* = −6 cm^−1^ with interplanar separation of 3.65 Å (Kremer *et al.*, 1994 ▸; Hicks *et al.*, 2001 ▸).

**Figure 12:**
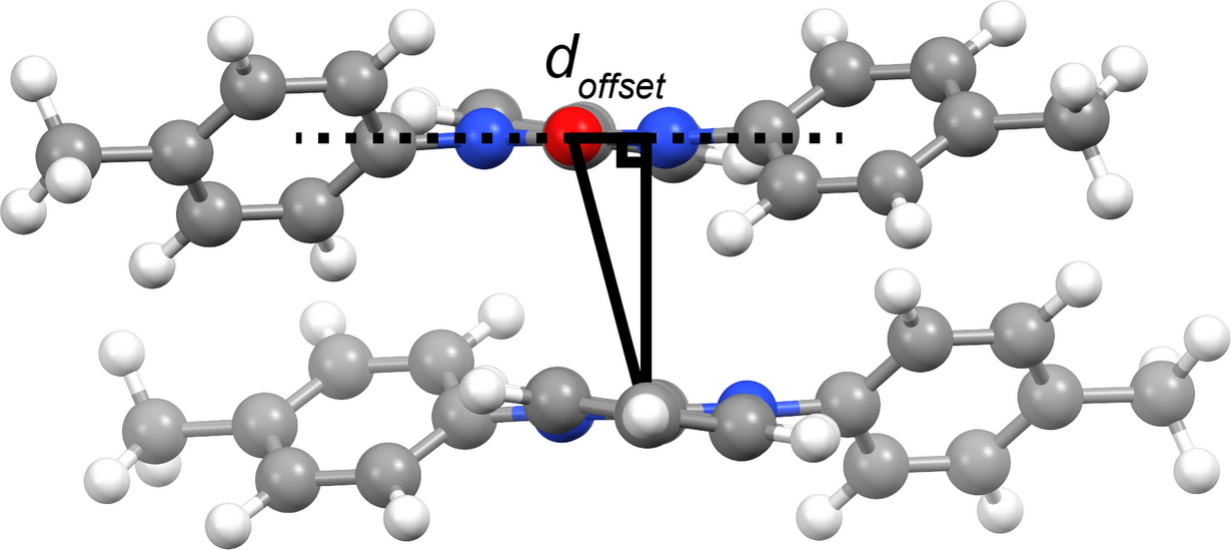
Representation of the horizontal offset distance in pTolOV dimers.

The offset distance, *d*_off_, is defined as the edge of the triangle formed by the distance between the mean plane through the verdazyl ring and centroid of the phenyl ring below, and the distance between the centroids of the verdazyl and phenyl rings (Fig. 12 ▸). This offset distance is a measure of the degree to which the verdazyl and phenyl rings are offset (slipped) from an eclipsed configuration. An increase in *d*_off_ is related to an increase in the distance between centroids of adjacent verdazyl rings. Under ambient conditions, the verdazyl and phenyl rings are offset by 0.27 and 0.93 Å with verdazyl–verdazyl centroid distances of 5.48 and 5.54 Å for the first and second dimer, respectively (using the dimer labels from previous sections). With the decrease in interplanar separation between the LP (at 0 GPa) and HP (at 2.73 GPa) phases of pTolOV, we would expect an increase in the strength of magnetic exchange interactions. The first dimer (*d*_p1_ = 3.66 Å) in the ambient structure has a magnetic exchange coupling parameter of *J* = −5.16 cm^−1^, while for the second dimer (*d*_p1_ = 3.51 Å) we see a value of *J* = −3.41 cm^−1^. The weaker magnetic interaction in the second dimer is a direct result of the increase in *d*_off_ and verdazyl–verdazyl distances, and occurs despite the shorter interplanar separation in the direction of the π-stacked chains.

Influence of the offset and verdazyl–verdazyl distances on the strength of magnetic interactions is observed in the dimers of the HP phase at 2.73 GPa. The *d*_off_ increases to 1.01 Å, and the verdazyl–verdazyl distance increases to 5.62 Å. Despite the distance between verdazyl ring planes decreasing, the strength of the magnetic interaction remains steady at *J* = −4.95/−4.75 cm^−1^. The role of verdazyl–verdazyl distance is consistent with the findings of Jornet *et al.*, that magnetic interactions within antiparallel chains of verdazyl radicals are a result of direct through space interactions between adjacent SOMOs (Jornet *et al.*, 2006 ▸). When we compare the strength of the magnetic interactions in both phases of pTolOV to those in the literature, the overlapping group (*i.e.* the phenyl group in pTolOV) has an influence on the magnetic interactions, as the 1,3,5-triphenyl-6-oxo-verdazyl radical exhibits a similar magnitude of *J* with larger interplanar separation (Kremer *et al.*, 1994 ▸). We can therefore deduce that magnetic interactions in verdazyl radicals crystals are a circular interplay of the distance between radical centres, and the composition of the overlapping moiety, both influencing the crystal structure, further affecting the magnetic topology.

**Figure 13:**
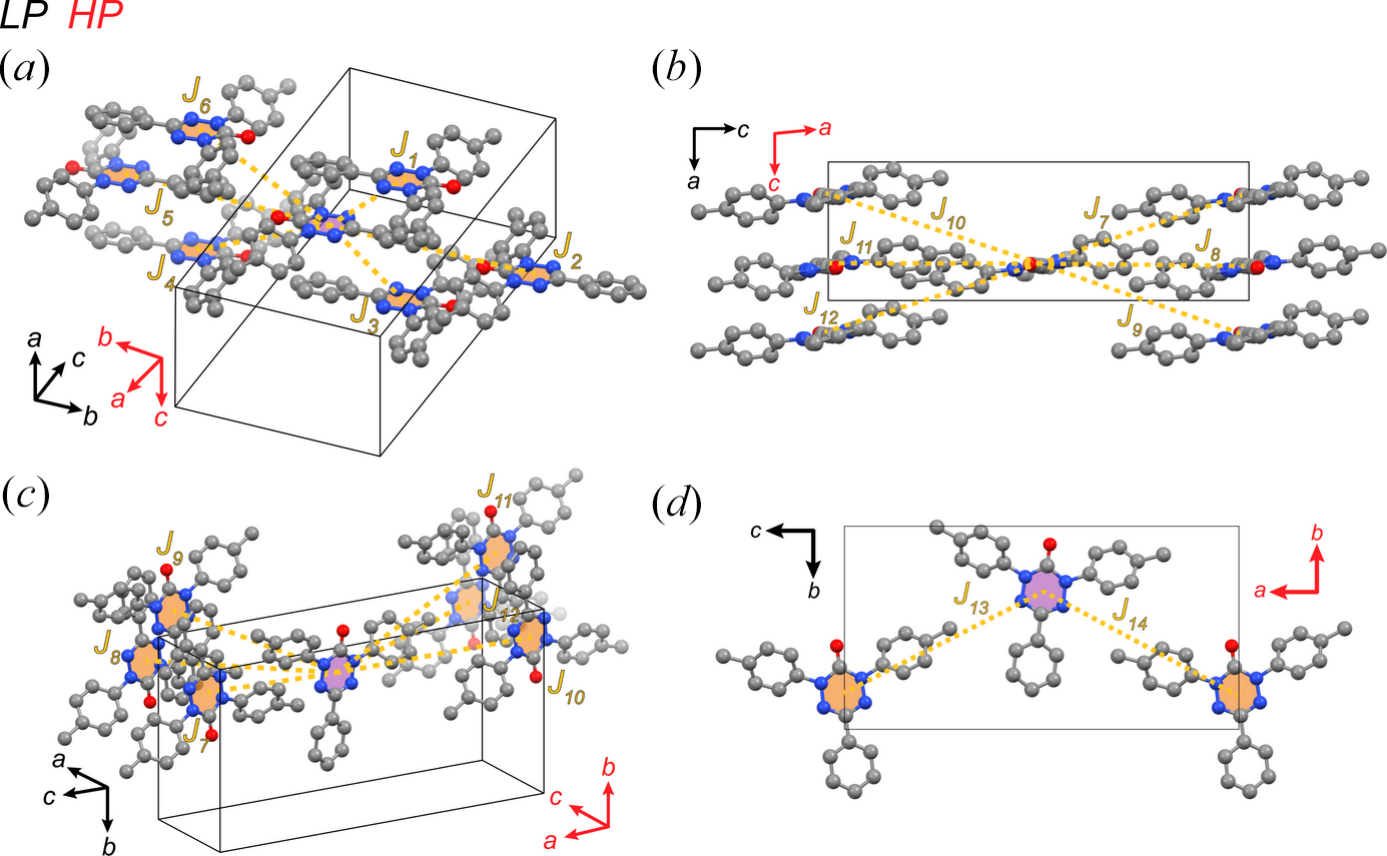
Potential magnetic interactions of the pTolOV asymmetric unit within 3.80 Å. Note: LP unit-cell axes are drawn, with the HP axis directions shown in red, and are only representative of the crystal orientation, due to subtle differences in crystal packing.

In order to gain a more complete picture of the magnetic topology of pTolOV in the solid state, an expanded view encompassing the full set of interactions that occur within the crystal surrounding the unit cell is required. Using the *CrystalExplorer* software package (Spackman *et al.*, 2021 ▸), the set of pTolOV molecules with closest next-neighbour distances within 3.8 Å of the asymmetric unit was obtained, producing 14 interactions total, for each of which *J* can be determined in the 0 and 2.73 GPa structures using the previous method. These magnetic interactions and their respective verdazyl–verdazyl distances are summarized in Table 4 ▸. Due to the similarities in packing, interactions *J*_1_ − *J*_6_, *J*_13_ and *J*_14_ are directly comparable. The configuration of these interactions in the pTolOV structure are shown in Fig. 13 ▸.

Whilst the magnetic exchange interactions are relatively weak in the planar *J*_1_ and *J*_3_ interactions, they are the predominate interactions in both phases of pTolOV. There are other interactions of interest that differ between phases, *J*_7_ and *J*_12_ are −1.01 and −1.16 cm^−1^, respectively, at ambient pressure, reducing to −0.74 and −0.72 cm^−1^, respectively, at 2.73 GPa. The *J*_7_ side-on interaction features adjacent tolyl groups. The phenyl-phenyl distance between these tolyl groups decreases from 4.93 Å at ambient pressure to 4.37 Å at 2.73 GPa. This reduction in contact distance may indicate that rather than the magnetic exchange interaction becoming negligible, it is becoming more ferromagnetic, and at higher pressures this may increase to beyond 0.1 cm^−1^. Another interaction that differs between phases is the head-on, oxygen-oxygen *J*_4_ and *J*_6_ interactions, which increase in magnitude to −0.61 and −1.18 cm^−1^, respectively, at 2.73 GPa, from −0.08 and 0.09 cm^−1^ at 0 GPa. This change in interaction strength corresponds to a decrease in distance between head-on oxygen atoms of adjacent molecules, from 4.12 Å at 0 GPa to 3.86 Å at 2.73 GPa. The repulsive nature of this interaction increasing as the distance between oxygen atoms decreases in space is the likely contributor to this increase in magnitude.

In the absence of experimental magnetic susceptibility data, these calculations provide an overview of the potential magnetic topology in pTolOV. There is a clear 1D antiferromagnetic chain that occurs between stacked antiparallel molecules, in the *a*-axis direction in the LP phase and the *c*-axis direction in the HP phase. There are weaker interactions that may also influence the overall topology, and provide 2D spin-ladder type behaviour. These interactions occur through side-on interactions at ambient pressure, and head-on interactions at 2.73 GPa. These results show that there is promise in verdazyl radicals as magnetic materials, and further design of verdazyl radicals with tuned crystal packing to optimize the distance between radical centres and relative orientation of molecules is a worthwhile prospect.

## 4. Conclusions

The effects of pressure on the structure, electronic and magnetic properties of the 1,5-tolyl-3-phenyl-6-oxo-verdazyl radical (pTolOV) has been studied, revealing a rare structural subgroup-to-supergroup, *P*2_1_/*n* to *C*2/*c* phase transition between 2.30 and 2.73 GPa. Prior to this transition, the unit cell of pTolOV undergoes anisotropic distortion, compression in the *a*-axis direction results in decreasing interplanar separation between antiparallel head-to-tail π-stacked molecules. The HP *C*2/*c* phase has increased symmetry, with *Z*′ = 0.5 and twofold screw axis intersecting the C—C bond of the verdazyl centre. This uncommon phase transition causes significant shortening of O—H contact lengths in the *C*(10) hydrogen-bond chain, falling in the top 0.02% of Ph—H⋯O=C distances of the Cambridge Structural Database (Groom *et al.*, 2016 ▸). There are also significant changes in intramolecular torsion angles, and intermolecular C—H⋯π interactions, driven by the reorientation of one tolyl moiety as they become symmetrically equivalent.

Periodic density functional theory reveals that the band gap for pTolOV (calculated at the Γ-point) remains in the range of a wide-gap semiconductor and an insulator, despite a reduction in energy between 0 and 2.30 GPa in the LP phase. DOS calculations reveal that the band gap is consistent with charge-transfer between the magnetic SOMOs of adjacent pTolOV molecules.

Density functional theory calculations reveal antiferromagnetic interactions between π-stacked antiparallel molecules, indicative of an alternating 1D antiferromagnetic chain. The decrease in interplanar separation between phases is counteracted by an increase in offset distance, causing the strength of magnetic interactions to not change significantly. Magnetic interactions of this kind are precedented and the strength lies within common literature experimental values, indicating reasonable accuracy in theoretical results. These calculations have added insights into the magnetism of verdazyl radicals, that the magnitude of magnetic interactions is driven by the distance between radicals.

This work provides valuable insights into the structure–property relationship of verdazyl radicals, and serves as the first study on the structural response to pressure of an organic verdazyl radical solid. Theoretical calculations on the magnetic and electrical properties provides direction and perspectives on the design of new materials for molecular electronics. This work serves as another spotlight on this class of organic radicals, and their prospects as materials for the electronics of the future. With a large pool from which to draw candidates for investigation into HP structure–property relationships, the promise of verdazyl radicals for future functional materials warrants further investigation.

## Supplementary Material

Crystal structure: contains datablock(s) pTolOV_LP_0MPa, pTolOV_LP_150MPa, pTolOV_LP_291MPa, pTolOV_LP_650MPa, pTolOV_LP_1220MPa, pTolOV_LP_1580MPa, pTolOV_LP_2300MPa, pTolOV_HP_2730MPa, pTolOV_HP_3310MPa. DOI: 10.1107/S2052520626006220/px5076sup1.cif

Structure factors: contains datablock(s) pTolOV_LP_0MPa Structure factors: contains datablock(s) pTolOV_LP_0MPa. DOI: 10.1107/S2052520626006220/px5076pTolOV_LP_0MPasup2.hkl

Structure factors: contains datablock(s) pTolOV_LP_0MPa Structure factors: contains datablock(s) pTolOV_LP_0MPa. DOI: 10.1107/S2052520626006220/px5076pTolOV_LP_0MPasup2.hkl

Structure factors: contains datablock(s) pTolOV_LP_150MPa. DOI: 10.1107/S2052520626006220/px5076pTolOV_LP_150MPasup3.hkl

Structure factors: contains datablock(s) pTolOV_LP_291MPa. DOI: 10.1107/S2052520626006220/px5076pTolOV_LP_291MPasup4.hkl

Structure factors: contains datablock(s) pTolOV_LP_650MPa. DOI: 10.1107/S2052520626006220/px5076pTolOV_LP_650MPasup5.hkl

Structure factors: contains datablock(s) pTolOV_LP_1220MPa. DOI: 10.1107/S2052520626006220/px5076pTolOV_LP_1220MPasup6.hkl

Structure factors: contains datablock(s) pTolOV_LP_1580MPa. DOI: 10.1107/S2052520626006220/px5076pTolOV_LP_1580MPasup7.hkl

Structure factors: contains datablock(s) pTolOV_LP_2300MPa. DOI: 10.1107/S2052520626006220/px5076pTolOV_LP_2300MPasup8.hkl

Structure factors: contains datablock(s) pTolOV_HP_2730MPa. DOI: 10.1107/S2052520626006220/px5076pTolOV_HP_2730MPasup9.hkl

Structure factors: contains datablock(s) pTolOV_HP_3310MPa. DOI: 10.1107/S2052520626006220/px5076pTolOV_HP_3310MPasup10.hkl

Revised Supporting Information. DOI: 10.1107/S2052520626006220/px5076sup11.pdf

CCDC references: 2505771, 2505772, 2505773, 2505774, 2505775, 2505776, 2505777, 2505778, 2505779

## Figures and Tables

**Table 1:** Space group, unit-cell dimensions, volume (*V*) and β angle during compression of pTolOV from ambient pressure to 3.31 GPa from single-crystal XRD

*P* (GPa)	Space group	*a* (Å)	*b* (Å)	*c* (Å)	β (°)	*V* (Å^3^)
0	*P*2_1_/*n*	7.2879 (1)	11.3796 (2)	22.1456 (4)	90.222 (2)	1836.60 (5)
0.15	*P*2_1_/*n*	7.2598 (6)	11.3921 (8)	22.11 (2)	90.15 (2)	1828.6 (19)
0.29	*P*2_1_/*n*	7.2217 (7)	11.3653 (6)	22.078 (18)	90.12 (2)	1812.1 (15)
0.65	*P*2_1_/*n*	7.0400 (7)	11.2598 (6)	21.833 (16)	89.95 (2)	1730.7 (13)
1.22	*P*2_1_/*n*	6.9151 (5)	11.1720 (6)	21.599 (14)	89.764 (18)	1668.6 (11)
1.58	*P*2_1_/*n*	6.8746 (8)	11.115 (2)	21.30 (3)	89.59 (3)	1628 (3)
2.30	*P*2_1_/*n*	6.800 (4)	11.047 (5)	21.19 (12)	89.19 (13)	1592 (9)
2.73	*C*2/*c*	19.71 (7)	11.302 (3)	7.002 (4)	95.88 (14)	1552 (6)
3.31	*C*2/*c*	19.520 (8)	11.222 (3)	7.013 (2)	96.05 (5)	1527.6 (9)

**Table 2:** Unit-cell parameters, volume and β angle of RPBE-D3BJ optimized pTolOV structures at varied pressures The last two rows display the experimental XRD primitive cell which was used in the optimization process.

*P* (GPa)	*a* (Å)	*b* (Å)	*c* (Å)	β (°)	*V* (Å^3^)
0	7.07	11.32	21.96	90.77	1756.89
0.15	7.00	11.28	21.82	90.72	1722.79
0.29	6.94	11.25	21.71	90.58	1695.97
0.65	6.83	11.17	21.52	90.19	1640.81
1.22	6.67	11.10	21.30	89.85	1577.39
1.58	6.61	11.05	21.15	89.63	1545.47
2.30	6.50	10.96	20.98	89.45	1493.94
2.73	11.24	11.24	6.73	95.46	731.85
3.31	11.19	11.19	6.64	95.60	716.84
2.73 (XRD)	11.36	11.36	7.00	95.10	775.79
3.31 (XRD)	11.26	11.26	7.01	95.24	763.83

**Table 3:** Calculated Γ-centred band gap at various pressures (*P*) Values calculated with HSE06 using RPBE-D3BJ optimized structures

*P* (GPa)	Band gap (eV)
0	2.0157
0.15	2.0079
0.29	1.9994
0.65	1.9858
1.22	1.9632
1.58	1.9620
2.30	1.9426
2.73	2.0216
3.31	2.0121

**Table 4:** Potential magnetic interactions of the asymmetric unit in pTolOV, their verdazyl–verdazyl centroid distances (*d*_verdazyl_), shortest non-hydrogen atom–atom distances (*d*_min_) and UB3LYP/6-31+G(d) calculated magnetic exchange coupling parameter values

	0 GPa			2.73 GPa		
Interaction	*d*_verdazyl_	*d*_min_	*J* (cm^−1^)	*d*_verdazyl_	*d*_min_	*J* (cm^−1^)
*J*_1_	5.54	3.42	−5.16	5.61	3.06	−4.95
*J*_2_	11.38	3.40	0.12	11.30	3.11	0.11
*J*_3_	5.48	3.48	−3.41	5.61	3.06	−4.75
*J*_4_	8.10	3.53	−0.08	7.75	3.57	−0.61
*J*_5_	11.38	3.40	−0.04	11.30	3.11	0.22
*J*_6_	8.14	3.75	0.09	7.75	3.57	−1.18
*J*_7_	11.78	3.64	−1.10	10.87	3.34	−0.74
*J*_8_	12.27	3.66	−0.08	11.36	3.34	−0.04
*J*_9_	11.75	3.87	0.08	10.19	3.83	0.15
*J*_10_	11.75	3.87	0.09	10.19	3.83	0.16
*J*_11_	12.63	3.81	−0.12	11.36	3.24	0.00
*J*_12_	11.78	3.64	−1.16	10.87	3.34	−0.72
*J*_13_	12.63	3.81	−0.12	11.36	3.24	−0.10
*J*_14_	12.27	3.66	−0.20	11.36	3.24	−0.09

## Data Availability

Supporting information contains XRD experimental details, DFT results, and input Cartesian coordinates for magnetic calculations. Crystal structures for each pressure point are included in the CIF.

## Footnotes

1 Based on a CCDC Conquest (Groom *et al.*, 2016 ▸) search for organic, non-polymeric compounds containing the oxo-verdazyl core, discounting deposits without 3D coordinates and different temperatures of already counted compounds.
