# Supplementary material for: Mapping structure–property relationships in a 6-oxo-verdazyl radical by variable pressure crystallography and density functional theory
Source: Acta Crystallogr B Struct Sci Cryst Eng Mater. 2026 Jun 26;82(Pt 4):416–32. doi: 10.1107/S2052520626006220 (PMC13470795; doi:10.1107/S2052520626006220)
Supplement: Supplementary file 1 [file b-82-00416-sup11.pdf]

Supporting Information to

Mapping structure-property relationships in a 6-oxo-verdazyl radical by variable pressure crystallography and density functional theory

J.R. Brookes[1], V.J. Kumar[1], I.M. Jones[2], A.M. Summers[1], S.A. Bird[3],
P.J. Low[1], S.A. Moggach[1], D. Spagnoli[1]

1. School of Molecular Sciences, University of Western Australia, Perth, 6009, Australia
2. School of Earth & Planetary Sciences, Curtin University, Bentley, 6102, Australia
3. Australian Synchrotron, 800 Blackburn Road, Clayton, 3168, Australia

Contents

S1 Experimental Details **2**

S2 Changes in intermolecular interactions **3**

S3 PDFT optimisation *k*-point grids **3**

S4 Gas-phase torsion scan computational details **3**

S5 Equations-of-state fitting **5**

S6 Transformation from $P2_1/n$ to $C2/c$ **6**

S7 Magnetic interaction dimer coordinates - CSD structures **7**

S8 Magnetic interactions dimer coordinates - pTolOV **9**

S8.1 0 GPa 9

S8.2 2.73 GPa 23

S1 Experimental Details

	0 GPa	0.15 GPa	0.291 GPa	0.65 GPa	1.22 GPa	1.58 GPa	2.30 GPa	2.73 GPa	3.31 GPa
Crystal system, space group	Monoclinic, $P2_1/n$	Monoclinic,$P2_1/n$	Monoclinic, $P2_1/n$	Monoclinic,$P2_1/n$	Monoclinic,$P2_1/n$	Monoclinic,$P2_1/n$	Monoclinic,$P2_1/n$	Monoclinic,$C2/c$	Monoclinic,$C2/c$
Temperature (K)	295	295	295	295	295	295	295	295	295
a, b, c (Å)	7.2879(1), 11.3796(2), 22.1456(4)	7.2598 (6), 11.3921 (8), 22.11 (2)	7.2217 (7), 11.3653 (6), 22.078 (18)	7.0400 (7), 11.2598 (7), 21.833 (16)	6.9151 (5), 11.1720 (6), 21.599 (14)	6.8746 (8), 11.115 (2), 21.30 (3)	6.800 (4), 11.047 (5), 21.19 (12)	19.71 (7), 11.302 (3), 7.002 (4)	19.520 (8), 11.222 (3), 7.013 (2)
β (°)	90.222 (2)	90.15 (2)	90.12 (2)	89.95 (2)	89.764 (18)	89.59 (3)	89.19 (13)	95.88 (14)	96.05 (5)
V (Å^3)	1836.60 (5)	1828.6 (19)	1812.1 (15)	1730.7 (13)	1668.6 (11)	1628 (3)	1592 (9)	1552 (6)	1527.6 (9)
Z	4	4	4	4	4	4	4	4	4
Radiation type	Cu Kα	Synchrotron, λ = 0.71073 Å	Synchrotron, λ = 0.71073 Å	Synchrotron, λ = 0.71073 Å	Synchrotron, λ = 0.71073 Å	Synchrotron, λ = 0.71073 Å	Synchrotron, λ = 0.71073 Å	Synchrotron, λ = 0.71073 Å	Synchrotron, λ = 0.71073 Å
μ (mm^{-1})	0.65	0.08	0.08	0.09	0.09	0.09	0.09	0.1	0.1
Crystal size (mm)	0.24 × 0.15 × 0.09	0.2 × 0.2 × 0.1	0.2 × 0.2 × 0.1	0.2 × 0.2 × 0.1	0.2 × 0.2 × 0.1	0.2 × 0.2 × 0.1	0.2 × 0.2 × 0.1	0.2 × 0.2 × 0.1	0.2 × 0.2 × 0.1
Diffractometer	XtaLAB Synergy, Single source at home/near, HyPix	MX1	MX1	MX1	MX1	MX1	MX1	MX1	MX1
Absorption correction	Multi-scan	Multi-scan	Multi-scan	Multi-scan	Multi-scan	Multi-scan	Multi-scan	Multi-scan	Multi-scan
	SADABS[1] (Bruker,2016/2) was used for absorption correction. wR2(int) was 0.0496 before and 0.0426 after correction. The Ratio of minimum to maximum transmission is 0.9151. The $\lambda/2$ correction factor is not present.	SADABS[1] (Bruker,2016/2) was used for absorption correction. wR2(int) was 0.0845 before and 0.0565 after correction. The Ratio of minimum to maximum transmission is 0.8592. The $\lambda/2$ correction factor is not present.	SADABS[1] (Bruker,2016/2) was used for absorption correction. wR2(int) was 0.1225 before and 0.0796 after correction. The Ratio of minimum to maximum transmission is 0.5680. The $\lambda/2$ correction factor is not present.	SADABS[1] (Bruker,2016/2) was used for absorption correction. wR2(int) was 0.1271 before and 0.0743 after correction. The Ratio of minimum to maximum transmission is 0.5696. The $\lambda/2$ correction factor is not present.	SADABS[1] (Bruker,2016/2) was used for absorption correction. wR2(int) was 0.1031 before and 0.0570 after correction. The Ratio of minimum to maximum transmission is 0.5715. The $\lambda/2$ correction factor is not present.	SADABS[1] (Bruker,2016/2) was used for absorption correction. wR2(int) was 0.1869 before and 0.0908 after correction. The Ratio of minimum to maximum transmission is 0.3416. The $\lambda/2$ correction factor is not present.	SADABS[1] (Bruker,2016/2) was used for absorption correction. wR2(int) was 0.1589 before and 0.0950 after correction. The Ratio of minimum to maximum transmission is 0.3403. The $\lambda/2$ correction factor is not present.	SADABS[1] (Bruker,2016/2) was used for absorption correction. wR2(int) was 0.0594 before and 0.0269 after correction. The Ratio of minimum to maximum transmission is 0.8351. The $\lambda/2$ correction factor is not present.	CrysAlis PRO 1.171.42.49 (Rigaku Oxford Diffraction, 2022) Empirical absorption correction using spherical harmonics, implemented in SCALE3 ABSPACK scaling algorithm.
T_{min}, T_{max}	0.690, 0.754	0.6397, 0.7445	0.423, 0.745	0.424, 0.745	0.426, 0.745	0.254, 0.745	0.253, 0.745	0.622, 0.745	0.232, 1.000
No. of measured, independent and observed [I >2σ(I)] reflections	9983, 3469, 3003	2394, 587, 380	7588, 792, 572	7612, 757, 576	2602, 639, 454	2223, 522, 451	2333, 560, 371	1130, 263, 180	1156, 282, 131
R_{int}	0.02	0.042	0.058	0.057	0.04	0.093	0.087	0.033	0.133
$\theta_{\max}$ (°)	75.2	20.6	20.8	20.8	20.8	20.8	20.9	20.7	20.8
$(\sin\theta/\lambda)_{\max}$ (Å^{-1})	0.627	0.495	0.5	0.5	0.499	0.499	0.503	0.497	0.5
$R[F^2 > 2\sigma(F^2)]$, $wR(F^2)$, S	0.048, 0.145, 1.09	0.105, 0.309, 1.15	0.094, 0.306, 1.22	0.088, 0.336, 1.42	0.080, 0.239, 1.06	0.100, 0.296, 1.08	0.138, 0.387, 1.31	0.116, 0.372, 1.51	0.181, 0.499, 1.57
No. of reflections	3469	587	792	757	639	522	560	263	282
No. of parameters	212	60	60	60	60	60	60	45	45
No. of restraints	0	21	21	21	21	21	21	19	19
H-atom treatment	H atoms treated by a mixture of independent and constrained refinement	H-atom parameters constrained	H-atom parameters constrained	H-atom parameters constrained	H-atom parameters constrained	H-atom parameters constrained	H-atom parameters constrained	H-atom parameters constrained	H-atom parameters constrained
Weighting scheme	$w = 1/[\sigma^2(F_o^2) + (0.071P)^2 + 0.4725P]$ where $P = (F_o^2 + 2F_c{}^2)/3$	$w = 1/[\sigma^2(F_o^2) + (0.2P)^2]$ where $P = (F_o^2+2F_c^2)/3$	$w = 1/[\sigma^2(F_o^2) + (0.2P)^2]$ where $P = (F_o^2+2F_c^2)/3$	$w = 1/[\sigma^2(F_o^2) + (0.2P)^2]$ where $P = (F_o^2+2F_c^2)/3$	$w = 1/[\sigma^2(F_o^2) + (0.2P)^2]$ where $P = (F_o^2+2F_c^2)/3$	$w = 1/[\sigma^2(F_o^2) + (0.2P)^2]$ where $P = (F_o^2+2F_c^2)/3$	$w = 1/[\sigma^2(F_o^2) + (0.2P)^2]$ where $P = (F_o^2+2F_c^2)/3$	$w = 1/[\sigma^2(F_o^2) + (0.2P)^2]$ where $P = (F_o^2+2F_c^2)/3$	$w = 1/[\sigma^2(F_o^2) + (0.2P)^2]$ where $P = (F_o^2+2F_c^2)/3$
$\delta\rho_{max}$, $\delta\rho_{min}$ (e.Å^3)	0.25, −0.26	0.19, −0.17	0.23, −0.23	0.28, −0.22	0.18, −0.17	0.21, −0.21	0.26, −0.28	0.22, −0.28	0.27, −0.40

S2 Changes in intermolecular interactions

Table S1: Changes in $C(10)$ chain distance and angle ($d_{C(10)}$ and ϕ), torsion angles T1, T2 and T3, C−H$\cdots\pi$ distances and angles ($d_{CH\pi 1}$, $d_{CH\pi 2}$, $\angle_{CH\pi 1}$, $\angle_{CH\pi 2}$) and interplanar separation (d_{p1} and d_{p2}) with pressure, P.

P (GPa)	$d_{C(10)}$ (Å)	ϕ (°)	T1 (°)	T2 (°)	T3 (°)	$d_{CH\pi 1}$ (Å)	$d_{CH\pi 2}$ (Å)	$\angle_{CH\pi 1}$ (°)	$\angle_{CH\pi 2}$ (°)	d_{p1} (Å)	d_{p2} (Å)
0	2.44	145.24	-47.00(18)	-41.15(19)	-8.45(19)	3.23	2.76	137.19	166.74	3.65	3.66
0.15	2.49	143.24	-44.4(8)	-37.9(8)	-10.3(8)	3.24	2.77	137.25	166.02	3.64	3.64
0.29	2.45	143.65	-46.9(5)	-40.1(5)	-7.1(5)	3.22	2.75	136.68	166.54	3.63	3.62
0.65	2.38	140.95	-46.6(6)	-39.0(6)	-7.3(6)	3.16	2.69	134.47	166.67	3.55	3.53
1.22	2.37	138.01	-48.1(6)	-38.8(6)	-7.5(6)	3.12	2.63	133.20	166.45	3.49	3.47
1.58	2.33	136.87	-49.9(7)	-39.6(7)	-8.6(7)	3.06	2.59	133.46	167.25	3.48	3.44
2.3	2.36	132.96	-49.6(12)	-39.1(11)	-8.5(12)	3.06	2.55	131.45	166.67	3.46	3.39
2.73	2.02	180.00	-47.5(16)	-47.5(16)	-16.9(10)	2.34	2.34	160.92	160.92	3.51	3.51
3.31	2.05	180.00	-42(2)	-42(2)	-19.5(14)	2.31	2.31	159.20	159.20	3.51	3.51

S3 PDFT optimisation *k*-point grids

Table S2: *k*-point grids generated with the automatic mesh generation feature of VASP, with density $R_k = 40$, both before geometry optimisation (initial) and post-optimisation (final). Differences between initial and final can be attributed to changes in the unit cell parameters during optimisation.

Pressure (GPa)	*k*-point grid	
	Initial	Final
0	5 × 4 × 2	6 × 4 × 2
0.15	6 × 4 × 2	6 × 4 × 2
0.291	6 × 4 × 2	6 × 4 × 2
0.65	6 × 4 × 2	6 × 4 × 2
1.22	6 × 4 × 2	6 × 4 × 2
1.58	6 × 4 × 2	6 × 4 × 2
2.3	6 × 4 × 2	6 × 4 × 2
2.73	4 × 4 × 6	4 × 4 × 6
3.31	4 × 4 × 6	4 × 4 × 6

S4 Gas-phase torsion scan computational details

The torsion scan was completed in Gaussian16 (revision A.02)[2], and the ROHF DSD-PBEP86-D3BJ[3–6]/def2-SVP[7] level of theory. The geometry of the asymmetric unit of pTolOV at 0 GPa was converted to a z-matrix, and the torsion angle T1, T2 and T3 were interpolated from their values at 0 GPa to their values at 2.30 GPa in 15 even points. This enabled direct analysis of the energy penalty to rotation of the tolyl and phenyl groups in isolation from any other structural changes. To track changes following the phase transition, the three torsion angles were rotated from their values at 2.73 GPa to 3.31 GPa across 5 even points. This produced the potential energy curve shown in Figure S1. The geometry of the ambient (0 GPa) structure is displayed in Table S4, and the T1, T2 and T3 values are displayed in Table S3.

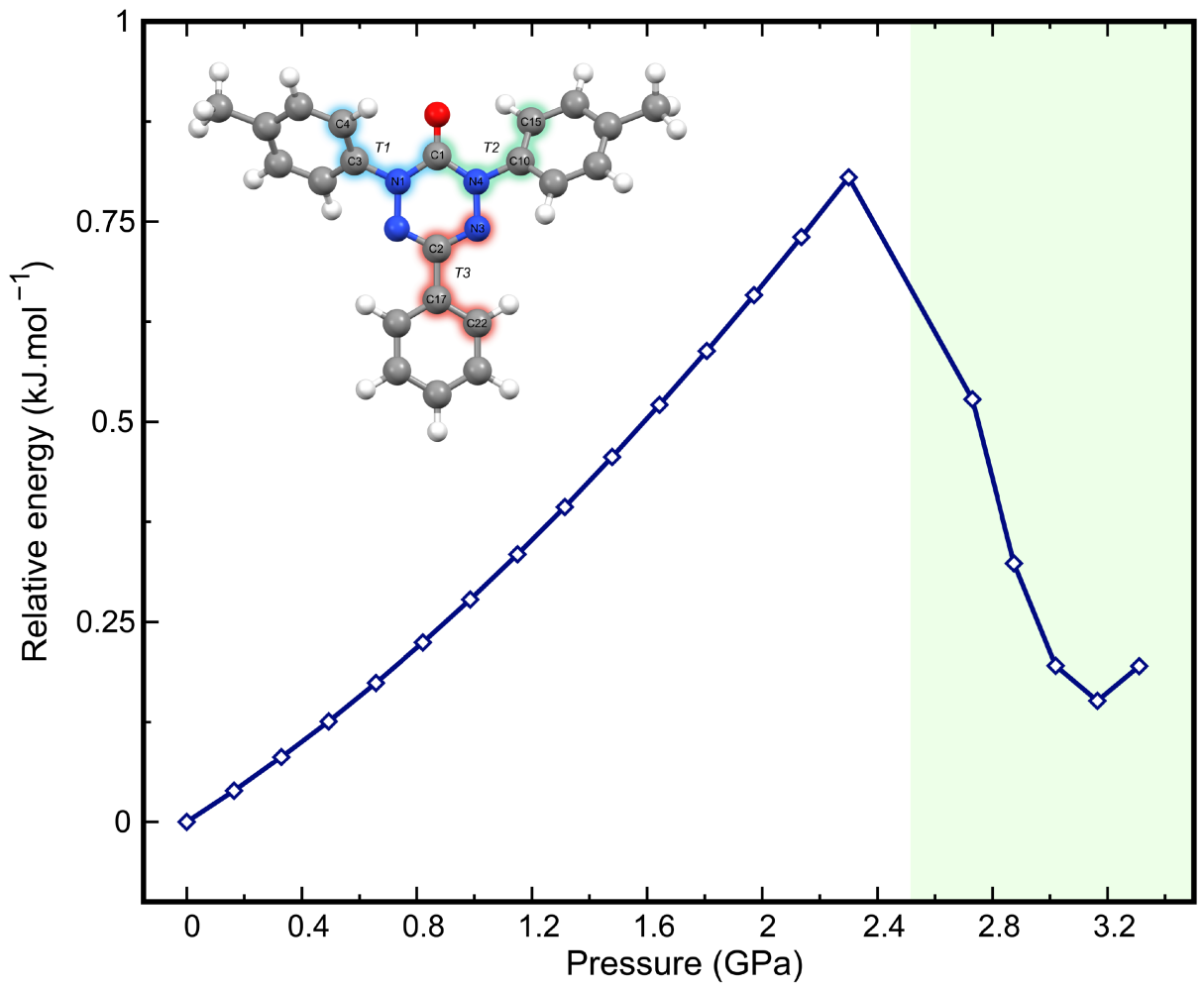

Figure S1: Potential energy curve of the torsion angle rotation of pTolOV, with the three rotated torsion angles in pTolOV inset. Energies are relative to 0 GPa.

Table S3: The values of torsion angles T1, T2 and T3 at each point of the potential energy curve.

Point	Pressure (GPa)	T1 (°)	T2 (°)	T3 (°)
0	0.00	−47.00	−41.15	−8.45
1	0.16	−47.33	−41.07	−8.28
2	0.33	−47.66	−40.99	−8.12
3	0.49	−47.99	−40.91	−7.95
4	0.66	−48.32	−40.84	−7.78
5	0.82	−48.65	−40.76	−7.62
6	0.99	−48.98	−40.68	−7.45
7	1.15	−49.32	−40.60	−7.29
8	1.31	−49.65	−40.52	−7.12
9	1.48	−49.98	−40.44	−6.95
10	1.64	−50.31	−40.36	−6.79
11	1.81	−50.64	−40.29	−6.62
12	1.97	−50.97	−40.21	−6.45
13	2.14	−51.30	−40.13	−6.29
14	2.30	−51.63	−40.05	−6.12
15	2.73	−46.38	−46.38	−16.87
16	2.88	−45.21	−45.21	−17.48
17	3.02	−44.04	−44.04	−18.08
18	3.17	−42.86	−42.86	−18.69
19	3.31	−41.69	−41.69	−19.29

Table S4: Cartesian coordinates of pTolOV at 0 GPa, used as the starting structure of the torsion angle scan. A z-matrix was used as the input into Gaussian 16, which was converted into these coordinates at the beginning of the calculation.

	x	y	z
O	0	0	0
C	0.00	0	1.209
N	1.16	0	1.973067
N	1.18	0.039393	3.340285
C	-0.01	0.037547	3.933908
N	-1.19	0.00747	3.342765
N	-1.16	0.002227	1.971591
C	-2.45	-0.09974	1.361518
C	-2.81	0.671513	0.261022
C	-4.10	0.61082	-0.24149
C	-5.04	-0.22113	0.356489
C	-4.69	-0.99238	1.456985
C	-3.39	-0.93169	1.9595
H	-2.07	1.323305	-0.20747
H	-4.38	1.215062	-1.10368
H	-5.42	-1.64417	1.925476
H	-3.11	-1.53593	2.821687
C	-6.46	-0.27573	-0.18093
H	-6.45	-0.60272	-1.21956
H	-6.91	0.714017	-0.11928
H	-7.05	-0.9776	0.411226
C	2.458179	0.066214	1.365413
C	2.777635	-0.64654	0.215617
C	4.068107	-0.59454	-0.29827
C	5.039122	0.170213	0.33764
C	4.719667	0.882967	1.487435
C	3.429195	0.830967	2.001322
H	2.01689	-1.24569	-0.28259
H	4.318385	-1.15295	-1.19908
H	5.480412	1.482115	1.98564
H	3.178916	1.389377	2.902133
C	6.455192	0.240576	-0.21579
H	6.43079	0.645313	-1.22649
H	6.887728	-0.75864	-0.2358
H	7.061703	0.885471	0.418388
C	-0.0054	0.063764	5.423677
C	1.187362	-0.08857	6.120962
C	1.190003	-0.04955	7.510412
C	-0.00012	0.141796	8.202576
C	-1.19289	0.294126	7.505291
C	-1.19553	0.25511	6.115841
H	2.119768	-0.23848	5.578684
H	2.124479	-0.1689	8.056702
H	0.001948	0.172363	9.291145
H	-2.12529	0.444038	8.04757
H	-2.13	0.374454	5.569551

S5 Equations-of-state fitting

The unit cell volume and unit cell parameters were fitted to a third-order Birch-Murnaghan equation of state,[8] shown in Equation 1:

$$P = \frac{3K_0}{2}\left[\left(\frac{V}{V_0}\right)^{7/3} - \left(\frac{V}{V_0}\right)^{5/3}\right] \tag{1}$$

where P = applied pressure, K_0 = isothermal bulk modulus, V = unit cell volume at applied pressure and V_0 = unit cell volume at ambient pressure. Applied pressure and unit cell volumes (V and V_0) were set to measured values.

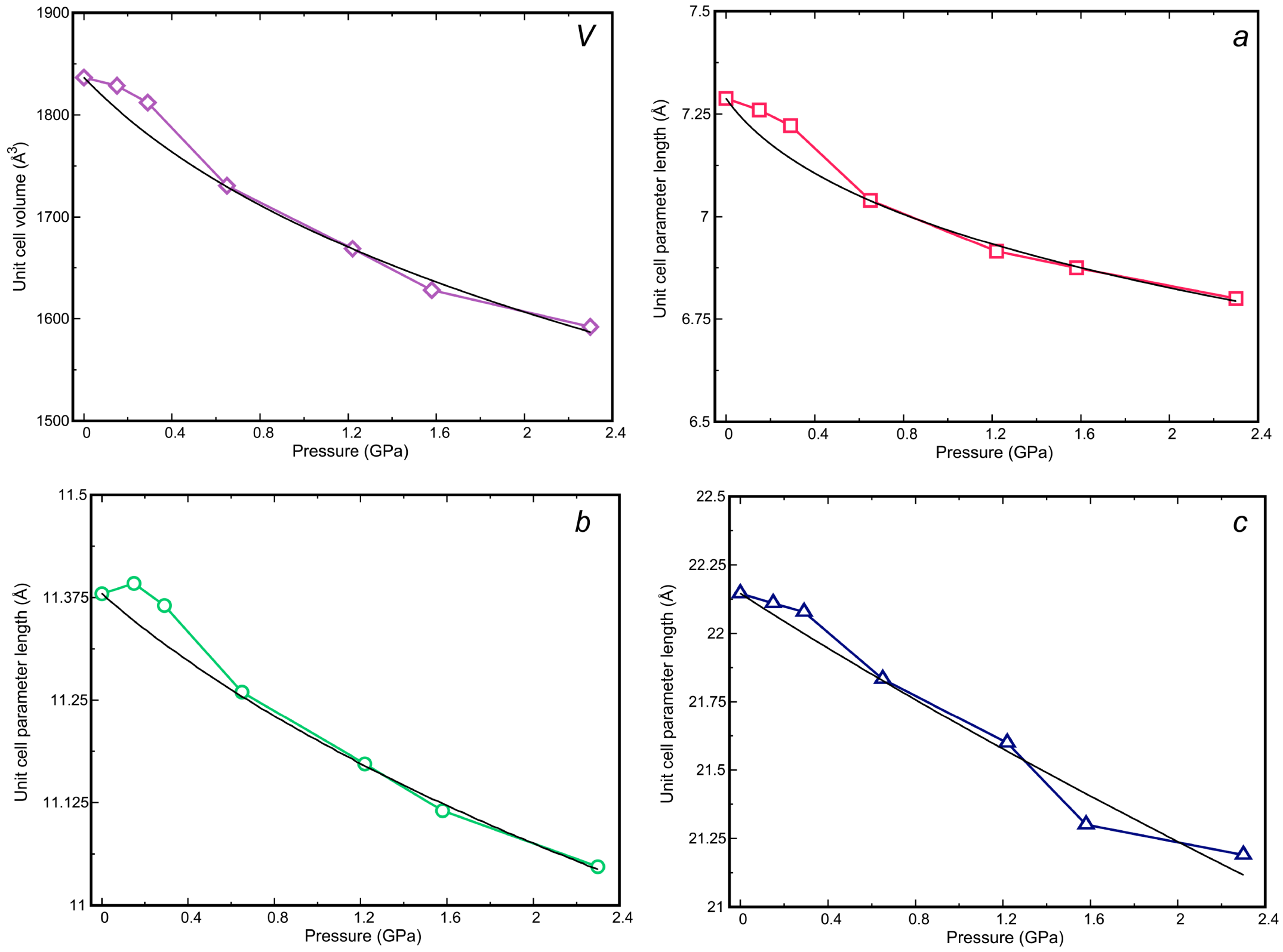

Figure S2: The volume, and unit cell parameters of pTolOV from $0 - 2.30$ GPa, with their fitted third-order Birch-Murnaghan curve shown in black.

The bulk modulus, K_0, of pTolOV between 0 and 2.30 GPa is 8.2(21) GPa, the linear moduli of the a-, b- and c-axes is 9.4(39) GPa, 49.9(130) GPa and 43.1(101) respectively.

S6 Transformation from $P2_1/n$ to $C2/c$

The transformation from the $P2_1/n$ unit cell (denoted as a, b, c) in the LP phase of pTolOV to the $C2/c$ unit cell (denoted as a', b', c') of the HP phase is defined as:

$$a' = c$$
$$b' = -b$$
$$c' = -a$$

Correspondingly, the transformation matrix used is:

$$\begin{pmatrix} a' \\ b' \\ c' \end{pmatrix} = \begin{pmatrix} 0 & 0 & 1 \\ 0 & 1 & 0 \\ -1 & 0 & 0 \end{pmatrix} \begin{pmatrix} a \\ b \\ c \end{pmatrix}$$

S7 Magnetic interaction dimer coordinates - CSD structures

Cartesian coordinates for the magnetic interaction calculations used as reference values are shown in the following tables. Crysttaolgraphic data for these structures was obtained from the CSD.[9] These include the interactions in the 1,5-dimethyl-3-(2-pyridyl)-6-oxo-verdazyl radical (CSD code: UBUBIC),[10] 3-(2 -imidazolyl)-1,5-dimethyl-6-oxo-verdazyl radical (CSD code: QIZBAD)[11] and the 1,5-dimethyl-3-(4′-carboxyphenyl)-6-oxo-verdazyl radical (CSD code: YEQPIW).[12]

In each compound, each dimer is extracted from a stacked trimer configuration and there is a common molecule between them, hence one dimer is made up of molecules 1 and 2, the other molecules 2 and 3.

Table S5: Cartesian coordinates of trimer configuration of the 1,5-dimethyl-3-(2-pyridyl)-6-oxo-verdazyl radical (CSD code: UBUBIC) of Hicks *et al.*,[10] in Å.

	Molecule 1				Molecule 2				Molecule 3		
	x	y	z		x	y	z		x	y	z
O	3.10422	4.843227	9.588591	O	6.09232	6.260652	0.970368	O	9.92422	4.843227	9.588591
N	2.950829	6.233142	7.797791	N	6.245711	4.870737	2.761168	N	9.770829	6.233142	7.797791
N	2.919287	6.442943	6.448884	N	6.277254	4.660936	4.110075	N	9.739287	6.442943	6.448884
N	2.887875	4.092379	6.144153	N	6.308666	7.0115	4.414806	N	9.707875	4.092379	6.144153
N	2.957768	3.950633	7.493271	N	6.238773	7.153246	3.065688	N	9.777768	3.950633	7.493271
N	2.761056	4.448666	3.479811	N	6.435484	6.655213	7.079149	N	9.581056	4.448666	3.479811
C	3.014891	5.000118	8.38487	C	6.181649	6.103761	2.17409	C	9.834891	5.000118	8.38487
C	2.887968	5.341638	5.716409	C	6.308573	5.76224	4.84255	C	9.707968	5.341638	5.716409
C	2.843902	5.548978	4.242801	C	6.352638	5.554901	6.316158	C	9.663902	5.548978	4.242801
C	2.868358	6.823703	3.706195	C	6.328182	4.280176	6.852765	C	9.688358	6.823703	3.706195
C	2.801578	6.962957	2.344089	C	6.394962	4.140922	8.21487	C	9.621578	6.962957	2.344089
C	2.725046	5.847018	1.546888	C	6.471495	5.256861	9.012072	C	9.545046	5.847018	1.546888
C	2.699683	4.628092	2.157195	C	6.496857	6.475787	8.401764	C	9.519683	4.628092	2.157195
C	2.957056	7.432047	8.62139	C	6.239484	3.671832	1.937569	C	9.777056	7.432047	8.62139
C	2.976934	2.581664	7.998412	C	6.219607	8.522215	2.560548	C	9.796934	2.581664	7.998412
H	2.939621	7.679828	4.355571	H	6.25692	3.42405	6.203389	H	9.759621	7.679828	4.355571
H	2.890588	7.859288	1.913283	H	6.305953	3.244591	8.645676	H	9.710588	7.859288	1.913283
H	2.645556	5.956084	0.499439	H	6.550984	5.147795	10.05952	H	9.465556	5.956084	0.499439
H	2.662203	3.741351	1.535273	H	6.534337	7.362527	9.023687	H	9.482203	3.741351	1.535273
H	2.36018	7.240854	9.442877	H	6.83636	3.863025	1.116082	H	9.18018	7.240854	9.442877
H	3.898653	7.69758	8.843128	H	5.297888	3.406299	1.715831	H	10.71865	7.69758	8.843128
H	2.556071	8.156217	8.125119	H	6.640469	2.947662	2.43384	H	9.376071	8.156217	8.125119
H	3.025539	2.68444	8.996233	H	6.171001	8.419439	1.562726	H	9.845539	2.68444	8.996233
H	2.239138	2.130329	7.528538	H	6.957402	8.97355	3.030421	H	9.059138	2.130329	7.528538
H	3.72634	2.168466	7.47152	H	5.4702	8.935413	3.08744	H	10.54634	2.168466	7.47152

Table S6: Cartesian coordinates of trimer configuration of the 3-(2′-imidazolyl)-1,5-dimethyl-6-oxo-verdazyl radical (CSD code: QIZBAD) of Norel *et al.*,[11] in Å.

	Molecule 1				Molecule 2				Molecule 3		
	x	y	z		x	y	z		x	y	z
O	2.947851	-1.62203	8.364811	O	6.268251	6.861125	10.25179	O	9.588651	-1.62203	8.364811
N	2.823668	1.919606	8.19149	N	6.144068	3.319494	10.42511	N	9.464468	1.919606	8.19149
N	2.824996	0.604592	7.830328	N	6.145396	4.634508	10.78627	N	9.465796	0.604592	7.830328
N	3.034846	-0.06182	10.0256	N	6.355246	5.300921	8.591002	N	9.675646	-0.06182	10.0256
N	3.021564	1.225949	10.46383	N	6.341964	4.013151	8.152768	N	9.662364	1.225949	10.46383
N	2.738666	3.939803	11.1748	N	6.059066	1.299297	7.4418	N	9.379466	3.939803	11.1748
H	2.55538	3.520675	11.86994	H	5.87578	1.718425	6.746656	H	9.19618	3.520675	11.86994
N	3.044143	4.573734	9.099236	N	6.364543	0.665366	9.517364	N	9.684943	4.573734	9.099236
C	2.93457	-0.43694	8.718712	C	6.25497	5.676041	9.897888	C	9.57537	-0.43694	8.718712
C	2.918632	2.132314	9.490743	C	6.239032	3.106786	9.125857	C	9.559432	2.132314	9.490743
C	2.90203	3.532201	9.91427	C	6.22243	1.706899	8.70233	C	9.54283	3.532201	9.91427
C	2.965117	5.668706	9.900308	C	6.285517	-0.42961	8.716292	C	9.605917	5.668706	9.900308
H	3.03551	6.547827	9.604304	H	6.35591	-1.30873	9.012296	H	9.67631	6.547827	9.604304
C	2.770542	5.301969	11.18113	C	6.090942	-0.06287	7.43547	C	9.411342	5.301969	11.18113
H	2.677571	5.861505	11.91835	H	5.997971	-0.62241	6.698253	H	9.318371	5.861505	11.91835
C	2.718744	0.347876	6.398712	C	6.039144	4.891224	12.21789	C	9.359544	0.347876	6.398712
H	2.609834	1.178798	5.931249	H	5.930234	4.060303	12.68535	H	9.250634	1.178798	5.931249
H	1.9597	-0.21585	6.230976	H	5.2801	5.454951	12.38562	H	8.6005	-0.21585	6.230976
H	3.516304	-0.09011	6.091352	H	6.836704	5.329213	12.52525	H	10.1571	-0.09011	6.091352
C	3.20485	-1.08345	11.05454	C	6.52525	6.322546	7.562063	C	9.84565	-1.08345	11.05454
H	4.051552	-1.52039	10.93539	H	7.371952	6.759487	7.681209	H	10.69235	-1.52039	10.93539
H	2.498269	-1.72995	10.98379	H	5.818669	6.969051	7.632806	H	9.139069	-1.72995	10.98379
H	3.176295	-0.6727	11.92207	H	6.496695	5.9118	6.694529	H	9.817095	-0.6727	11.92207

Table S7: Cartesian coordinates of trimer configuration of the 1,5-dimethyl-3-(4 -carboxyphenyl)-6-oxo-verdazyl radical (CSD code: YEQPIW) of Kumar *et al.*,[12] in Å.

	Molecule 1				Molecule 2				Molecule 3		
	x	y	z		x	y	z		x	y	z
O	4.80256	8.00348	6.250112	O	9.106291	1.07606	15.9441	O	11.74266	8.00348	6.250112
H	4.800771	8.290475	5.481971	H	9.108081	0.789065	16.71224	H	11.74087	8.290475	5.481971
O	5.22509	6.094116	5.230843	O	8.683762	2.985424	16.96337	O	12.16519	6.094116	5.230843
O	5.234726	2.908493	15.13324	O	8.674126	6.171047	7.060978	O	12.17483	2.908493	15.13324
N	5.110999	4.699462	13.75986	N	8.797853	4.380078	8.434356	N	12.0511	4.699462	13.75986
N	5.37526	2.600018	12.88208	N	8.533592	6.479522	9.312137	N	12.31536	2.600018	12.88208
N	5.356383	3.087186	11.60158	N	8.552469	5.992354	10.59263	N	12.29648	3.087186	11.60158
N	5.104016	5.265867	12.52087	N	8.804836	3.813673	9.673348	N	12.04412	5.265867	12.52087
C	5.064483	6.715926	6.236574	C	8.844369	2.363614	15.95764	C	12.00458	6.715926	6.236574
C	5.138495	6.140519	7.609175	C	8.770357	2.939021	14.58504	C	12.0786	6.140519	7.609175
C	5.343749	4.784459	7.762426	C	8.565103	4.295082	14.43179	C	12.28385	4.784459	7.762426
H	5.459937	4.245018	7.013371	H	8.448915	4.834522	15.18084	H	12.40004	4.245018	7.013371
C	5.375067	4.225783	9.025277	C	8.533785	4.853757	13.16894	C	12.31517	4.225783	9.025277
H	5.515683	3.311128	9.117383	H	8.393169	5.768412	13.07683	H	12.45578	3.311128	9.117383
C	5.202763	5.001782	10.15463	C	8.706089	4.077758	12.03958	C	12.14286	5.001782	10.15463
C	5.012189	6.361715	9.994054	C	8.896663	2.717825	12.20016	C	11.95229	6.361715	9.994054
H	4.906013	6.903287	10.742	H	9.002839	2.176253	11.45221	H	11.84611	6.903287	10.742
C	4.976276	6.92229	8.736752	C	8.932576	2.15725	13.45746	C	11.91638	6.92229	8.736752
H	4.842346	7.837769	8.645756	H	9.066506	1.241771	13.54846	H	11.78245	7.837769	8.645756
C	5.228991	4.410791	11.51658	C	8.679861	4.668749	10.67764	C	12.16909	4.410791	11.51658
C	5.240204	3.36439	13.99456	C	8.668647	5.71515	8.199652	C	12.1803	3.36439	13.99456
C	5.504187	1.153227	12.99804	C	8.404664	7.926313	9.196172	C	12.44429	1.153227	12.99804
H	4.662386	0.739572	12.79385	H	9.246466	8.339969	9.400359	H	11.60249	0.739572	12.79385
H	5.76446	0.926176	13.89358	H	8.144392	8.153364	8.300636	H	12.70456	0.926176	13.89358
H	6.171641	0.841079	12.38215	H	7.73721	8.238461	9.812062	H	13.11174	0.841079	12.38215
C	4.960378	5.628841	14.86679	C	8.948474	3.450699	7.327419	C	11.90048	5.628841	14.86679
H	5.79551	5.70947	15.33287	H	8.113342	3.37007	6.861341	H	12.73561	5.70947	15.33287
H	4.288329	5.301882	15.46937	H	9.620523	3.777658	6.724847	H	11.22843	5.301882	15.46937
H	4.696548	6.487934	14.53055	H	9.212304	2.591606	7.663662	H	11.63665	6.487934	14.53055

S8 Magnetic interactions dimer coordinates - pTolOV

S8.1 0 GPa

Table S8: Cartesian coordinates of J_1 in pTolOV at 0 GPa.

	Molecule 1				Molecule 2		
	x	y	z		x	y	z
O	5.33508	1.02223	10.63424	O	9.15491	10.35737	11.5112
N	5.60391	3.11619	9.7544	N	8.88608	8.26341	12.39104
N	5.21523	4.19395	12.22251	N	9.27476	7.18565	9.92293
N	5.1825	2.83773	12.01811	N	9.30749	8.54187	10.12733
N	5.61123	4.47673	9.89635	N	8.87876	6.90287	12.24908
C	5.41696	4.92293	11.13273	C	9.07304	6.45667	11.0127
C	5.43702	6.40159	11.31565	C	9.05297	4.97801	10.82978
C	5.82327	7.22559	10.26507	C	8.66672	4.15401	11.88036
H	6.12189	6.78977	9.3128	H	8.36811	4.58983	12.83264
C	5.82846	8.60571	10.42984	C	8.66153	2.77389	11.7156
H	6.13106	9.25122	9.60662	H	8.35894	2.12838	12.53881
C	5.44726	9.16206	11.64518	C	9.04274	2.21754	10.50026
H	5.45134	10.24337	11.77428	H	9.03866	1.13623	10.37115
C	5.06101	8.33817	12.69598	C	9.42898	3.04143	9.44946
H	4.76237	8.77396	13.64825	H	9.72762	2.60564	8.49718
C	5.05582	6.95794	12.53122	C	9.43418	4.42166	9.61422
H	4.75321	6.31245	13.35443	H	9.73679	5.06715	8.79101
C	5.75573	2.67341	8.39666	C	8.73426	8.70619	13.74877
C	6.47992	1.53488	8.06293	C	8.01008	9.84472	14.0825
H	6.91685	0.91817	8.84695	H	7.57315	10.46143	13.29849
C	6.64637	1.18337	6.72845	C	7.84363	10.19624	15.41699
H	7.21372	0.29137	6.46695	H	7.27627	11.08823	15.67848
C	6.08864	1.97049	5.72769	C	8.40136	9.40911	16.41774
C	5.36453	3.10902	6.06143	C	9.12546	8.27058	16.08401
H	4.92758	3.72567	5.27737	H	9.56241	7.65393	16.86807
C	5.19801	3.46054	7.39591	C	9.29199	7.91906	14.74952
H	4.63067	4.35256	7.65734	H	9.85932	7.02704	14.4881
C	5.37165	2.22039	10.78837	C	9.11834	9.15921	11.35706
C	4.64805	0.75891	15.63822	C	9.84195	10.62069	6.50721
C	3.98249	0.32341	14.49839	C	10.50751	11.05619	7.64704
H	3.31133	-0.53243	14.55325	H	11.17867	11.91203	7.59218
C	4.17345	0.98035	13.28837	C	10.31654	10.39925	8.85707
H	3.65197	0.63917	12.39531	H	10.83803	10.74043	9.75013
C	5.03005	2.07279	13.21839	C	9.45994	9.30681	8.92705
C	5.69569	2.50829	14.35821	C	8.79431	8.87131	7.78722
H	6.36676	3.36419	14.30327	H	8.12324	8.01542	7.84216
C	5.50472	1.85146	15.56824	C	8.98527	9.52814	6.57719
H	6.02622	2.19264	16.4613	H	8.46377	9.18696	5.68414
C	6.25982	1.59542	4.26278	C	8.23017	9.78418	17.88266
H	5.75421	2.33043	3.63825	H	8.73578	9.04917	18.50718
H	7.31994	1.57611	4.01448	H	7.17006	9.80349	18.13095
H	5.82803	0.61124	4.08707	H	8.66196	10.76836	18.05836
C	4.42651	0.05235	16.96894	C	10.06348	11.32725	5.1765
H	5.02869	0.5318	17.73929	H	9.4613	10.8478	4.40614
H	3.37381	0.11233	17.24124	H	11.11618	11.26727	4.90419
H	4.71894	-0.99271	16.87805	H	9.77105	12.37231	5.26738

Table S9: Cartesian coordinates of J_2 in pTolOV at 0 GPa.

	Molecule 1				Molecule 2		
	x	y	z		x	y	z
O	5.33508	1.02223	10.63424	O	5.33508	12.40183	10.63424
N	5.60391	3.11619	9.7544	N	5.60391	14.49579	9.7544
N	5.21523	4.19395	12.22251	N	5.21523	15.57355	12.22251
N	5.1825	2.83773	12.01811	N	5.1825	14.21733	12.01811
N	5.61123	4.47673	9.89635	N	5.61123	15.85634	9.89635
C	5.41696	4.92293	11.13273	C	5.41696	16.30253	11.13273
C	5.43702	6.40159	11.31565	C	5.43702	17.78119	11.31565
C	5.82327	7.22559	10.26507	C	5.82327	18.60519	10.26507
H	6.12189	6.78977	9.3128	H	6.12189	18.16937	9.3128
C	5.82846	8.60571	10.42984	C	5.82846	19.98531	10.42984
H	6.13106	9.25122	9.60662	H	6.13106	20.63082	9.60662
C	5.44726	9.16206	11.64518	C	5.44726	20.54166	11.64518
H	5.45134	10.24337	11.77428	H	5.45134	21.62297	11.77428
C	5.06101	8.33817	12.69598	C	5.06101	19.71777	12.69598
H	4.76237	8.77396	13.64825	H	4.76237	20.15356	13.64825
C	5.05582	6.95794	12.53122	C	5.05582	18.33754	12.53122
H	4.75321	6.31245	13.35443	H	4.75321	17.69205	13.35443
C	5.75573	2.67341	8.39666	C	5.75573	14.05301	8.39666
C	6.47992	1.53488	8.06293	C	6.47992	12.91448	8.06293
H	6.91685	0.91817	8.84695	H	6.91685	12.29777	8.84695
C	6.64637	1.18337	6.72845	C	6.64637	12.56297	6.72845
H	7.21372	0.29137	6.46695	H	7.21372	11.67097	6.46695
C	6.08864	1.97049	5.72769	C	6.08864	13.35009	5.72769
C	5.36453	3.10902	6.06143	C	5.36453	14.48862	6.06143
H	4.92758	3.72567	5.27737	H	4.92758	15.10527	5.27737
C	5.19801	3.46054	7.39591	C	5.19801	14.84014	7.39591
H	4.63067	4.35256	7.65734	H	4.63067	15.73216	7.65734
C	5.37165	2.22039	10.78837	C	5.37165	13.59999	10.78837
C	4.64805	0.75891	15.63822	C	4.64805	12.13851	15.63822
C	3.98249	0.32341	14.49839	C	3.98249	11.70301	14.49839
H	3.31133	-0.53243	14.55325	H	3.31133	10.84717	14.55325
C	4.17345	0.98035	13.28837	C	4.17345	12.35995	13.28837
H	3.65197	0.63917	12.39531	H	3.65197	12.01877	12.39531
C	5.03005	2.07279	13.21839	C	5.03005	13.45239	13.21839
C	5.69569	2.50829	14.35821	C	5.69569	13.88789	14.35821
H	6.36676	3.36419	14.30327	H	6.36676	14.74379	14.30327
C	5.50472	1.85146	15.56824	C	5.50472	13.23106	15.56824
H	6.02622	2.19264	16.4613	H	6.02622	13.57224	16.4613
C	6.25982	1.59542	4.26278	C	6.25982	12.97502	4.26278
H	5.75421	2.33043	3.63825	H	5.75421	13.71003	3.63825
H	7.31994	1.57611	4.01448	H	7.31994	12.95571	4.01448
H	5.82803	0.61124	4.08707	H	5.82803	11.99084	4.08707
C	4.42651	0.05235	16.96894	C	4.42651	11.43195	16.96894
H	5.02869	0.5318	17.73929	H	5.02869	11.9114	17.73929
H	3.37381	0.11233	17.24124	H	3.37381	11.49193	17.24124
H	4.71894	-0.99271	16.87805	H	4.71894	10.38689	16.87805

Table S10: Cartesian coordinates of J_3 in pTolOV at 0 GPa.

	Molecule 1				Molecule 2		
	x	y	z		x	y	z
O	5.33508	1.02223	10.63424	O	1.86701	10.35737	11.5112
N	5.60391	3.11619	9.7544	N	1.59818	8.26341	12.39104
N	5.21523	4.19395	12.22251	N	1.98686	7.18565	9.92293
N	5.1825	2.83773	12.01811	N	2.01959	8.54187	10.12733
N	5.61123	4.47673	9.89635	N	1.59086	6.90287	12.24908
C	5.41696	4.92293	11.13273	C	1.78513	6.45667	11.0127
C	5.43702	6.40159	11.31565	C	1.76507	4.97801	10.82978
C	5.82327	7.22559	10.26507	C	1.37883	4.15401	11.88036
H	6.12189	6.78977	9.3128	H	1.08021	4.58983	12.83264
C	5.82846	8.60571	10.42984	C	1.37363	2.77389	11.7156
H	6.13106	9.25122	9.60662	H	1.07104	2.12838	12.53881
C	5.44726	9.16206	11.64518	C	1.75483	2.21754	10.50026
H	5.45134	10.24337	11.77428	H	1.75076	1.13623	10.37115
C	5.06101	8.33817	12.69598	C	2.14108	3.04143	9.44946
H	4.76237	8.77396	13.64825	H	2.43972	2.60564	8.49718
C	5.05582	6.95794	12.53122	C	2.14628	4.42166	9.61422
H	4.75321	6.31245	13.35443	H	2.44889	5.06715	8.79101
C	5.75573	2.67341	8.39666	C	1.44636	8.70619	13.74877
C	6.47992	1.53488	8.06293	C	0.72218	9.84472	14.0825
H	6.91685	0.91817	8.84695	H	0.28525	10.46143	13.29849
C	6.64637	1.18337	6.72845	C	0.55573	10.19624	15.41699
H	7.21372	0.29137	6.46695	H	-0.01163	11.08823	15.67848
C	6.08864	1.97049	5.72769	C	1.11346	9.40911	16.41774
C	5.36453	3.10902	6.06143	C	1.83756	8.27058	16.08401
H	4.92758	3.72567	5.27737	H	2.27451	7.65393	16.86807
C	5.19801	3.46054	7.39591	C	2.00409	7.91906	14.74952
H	4.63067	4.35256	7.65734	H	2.57142	7.02704	14.4881
C	5.37165	2.22039	10.78837	C	1.83044	9.15921	11.35706
C	4.64805	0.75891	15.63822	C	2.55405	10.62069	6.50721
C	3.98249	0.32341	14.49839	C	3.21961	11.05619	7.64704
H	3.31133	-0.53243	14.55325	H	3.89077	11.91203	7.59218
C	4.17345	0.98035	13.28837	C	3.02864	10.39925	8.85707
H	3.65197	0.63917	12.39531	H	3.55013	10.74043	9.75013
C	5.03005	2.07279	13.21839	C	2.17204	9.30681	8.92705
C	5.69569	2.50829	14.35821	C	1.50641	8.87131	7.78722
H	6.36676	3.36419	14.30327	H	0.83534	8.01542	7.84216
C	5.50472	1.85146	15.56824	C	1.69738	9.52814	6.57719
H	6.02622	2.19264	16.4613	H	1.17587	9.18696	5.68414
C	6.25982	1.59542	4.26278	C	0.94227	9.78418	17.88266
H	5.75421	2.33043	3.63825	H	1.44788	9.04917	18.50718
H	7.31994	1.57611	4.01448	H	-0.11784	9.80349	18.13095
H	5.82803	0.61124	4.08707	H	1.37406	10.76836	18.05836
C	4.42651	0.05235	16.96894	C	2.77558	11.32725	5.1765
H	5.02869	0.5318	17.73929	H	2.1734	10.8478	4.40614
H	3.37381	0.11233	17.24124	H	3.82828	11.26727	4.90419
H	4.71894	-0.99271	16.87805	H	2.48315	12.37231	5.26738

Table S11: Cartesian coordinates of J_4 in pTolOV at 0 GPa.

	Molecule 1				Molecule 2		
	x	y	z		x	y	z
O	5.33508	1.02223	10.63424	O	1.86701	-1.02223	11.5112
N	5.60391	3.11619	9.7544	N	1.59818	-3.11619	12.39104
N	5.21523	4.19395	12.22251	N	1.98686	-4.19395	9.92293
N	5.1825	2.83773	12.01811	N	2.01959	-2.83773	10.12733
N	5.61123	4.47673	9.89635	N	1.59086	-4.47673	12.24908
C	5.41696	4.92293	11.13273	C	1.78513	-4.92293	11.0127
C	5.43702	6.40159	11.31565	C	1.76507	-6.40159	10.82978
C	5.82327	7.22559	10.26507	C	1.37883	-7.22559	11.88036
H	6.12189	6.78977	9.3128	H	1.08021	-6.78977	12.83264
C	5.82846	8.60571	10.42984	C	1.37363	-8.60571	11.7156
H	6.13106	9.25122	9.60662	H	1.07104	-9.25122	12.53881
C	5.44726	9.16206	11.64518	C	1.75483	-9.16206	10.50026
H	5.45134	10.24337	11.77428	H	1.75076	-10.2434	10.37115
C	5.06101	8.33817	12.69598	C	2.14108	-8.33817	9.44946
H	4.76237	8.77396	13.64825	H	2.43972	-8.77396	8.49718
C	5.05582	6.95794	12.53122	C	2.14628	-6.95794	9.61422
H	4.75321	6.31245	13.35443	H	2.44889	-6.31245	8.79101
C	5.75573	2.67341	8.39666	C	1.44636	-2.67341	13.74877
C	6.47992	1.53488	8.06293	C	0.72218	-1.53488	14.0825
H	6.91685	0.91817	8.84695	H	0.28525	-0.91817	13.29849
C	6.64637	1.18337	6.72845	C	0.55573	-1.18337	15.41699
H	7.21372	0.29137	6.46695	H	-0.01163	-0.29137	15.67848
C	6.08864	1.97049	5.72769	C	1.11346	-1.97049	16.41774
C	5.36453	3.10902	6.06143	C	1.83756	-3.10902	16.08401
H	4.92758	3.72567	5.27737	H	2.27451	-3.72567	16.86807
C	5.19801	3.46054	7.39591	C	2.00409	-3.46054	14.74952
H	4.63067	4.35256	7.65734	H	2.57142	-4.35256	14.4881
C	5.37165	2.22039	10.78837	C	1.83044	-2.22039	11.35706
C	4.64805	0.75891	15.63822	C	2.55405	-0.75891	6.50721
C	3.98249	0.32341	14.49839	C	3.21961	-0.32341	7.64704
H	3.31133	-0.53243	14.55325	H	3.89077	0.53243	7.59218
C	4.17345	0.98035	13.28837	C	3.02864	-0.98035	8.85707
H	3.65197	0.63917	12.39531	H	3.55013	-0.63917	9.75013
C	5.03005	2.07279	13.21839	C	2.17204	-2.07279	8.92705
C	5.69569	2.50829	14.35821	C	1.50641	-2.50829	7.78722
H	6.36676	3.36419	14.30327	H	0.83534	-3.36419	7.84216
C	5.50472	1.85146	15.56824	C	1.69738	-1.85146	6.57719
H	6.02622	2.19264	16.4613	H	1.17587	-2.19264	5.68414
C	6.25982	1.59542	4.26278	C	0.94227	-1.59542	17.88266
H	5.75421	2.33043	3.63825	H	1.44788	-2.33043	18.50718
H	7.31994	1.57611	4.01448	H	-0.11784	-1.57611	18.13095
H	5.82803	0.61124	4.08707	H	1.37406	-0.61124	18.05836
C	4.42651	0.05235	16.96894	C	2.77558	-0.05235	5.1765
H	5.02869	0.5318	17.73929	H	2.1734	-0.5318	4.40614
H	3.37381	0.11233	17.24124	H	3.82828	-0.11233	4.90419
H	4.71894	-0.99271	16.87805	H	2.48315	0.99271	5.26738

Table S12: Cartesian coordinates of J_5 in pTolOV at 0 GPa.

	Molecule 1				Molecule 2		
	x	y	z		x	y	z
O	5.33508	1.02223	10.63424	O	5.33508	-10.3574	10.63424
N	5.60391	3.11619	9.7544	N	5.60391	-8.26341	9.7544
N	5.21523	4.19395	12.22251	N	5.21523	-7.18565	12.22251
N	5.1825	2.83773	12.01811	N	5.1825	-8.54187	12.01811
N	5.61123	4.47673	9.89635	N	5.61123	-6.90287	9.89635
C	5.41696	4.92293	11.13273	C	5.41696	-6.45667	11.13273
C	5.43702	6.40159	11.31565	C	5.43702	-4.97801	11.31565
C	5.82327	7.22559	10.26507	C	5.82327	-4.15401	10.26507
H	6.12189	6.78977	9.3128	H	6.12189	-4.58983	9.3128
C	5.82846	8.60571	10.42984	C	5.82846	-2.77389	10.42984
H	6.13106	9.25122	9.60662	H	6.13106	-2.12838	9.60662
C	5.44726	9.16206	11.64518	C	5.44726	-2.21754	11.64518
H	5.45134	10.24337	11.77428	H	5.45134	-1.13623	11.77428
C	5.06101	8.33817	12.69598	C	5.06101	-3.04143	12.69598
H	4.76237	8.77396	13.64825	H	4.76237	-2.60564	13.64825
C	5.05582	6.95794	12.53122	C	5.05582	-4.42166	12.53122
H	4.75321	6.31245	13.35443	H	4.75321	-5.06715	13.35443
C	5.75573	2.67341	8.39666	C	5.75573	-8.70619	8.39666
C	6.47992	1.53488	8.06293	C	6.47992	-9.84472	8.06293
H	6.91685	0.91817	8.84695	H	6.91685	-10.4614	8.84695
C	6.64637	1.18337	6.72845	C	6.64637	-10.1962	6.72845
H	7.21372	0.29137	6.46695	H	7.21372	-11.0882	6.46695
C	6.08864	1.97049	5.72769	C	6.08864	-9.40911	5.72769
C	5.36453	3.10902	6.06143	C	5.36453	-8.27058	6.06143
H	4.92758	3.72567	5.27737	H	4.92758	-7.65393	5.27737
C	5.19801	3.46054	7.39591	C	5.19801	-7.91906	7.39591
H	4.63067	4.35256	7.65734	H	4.63067	-7.02704	7.65734
C	5.37165	2.22039	10.78837	C	5.37165	-9.15921	10.78837
C	4.64805	0.75891	15.63822	C	4.64805	-10.6207	15.63822
C	3.98249	0.32341	14.49839	C	3.98249	-11.0562	14.49839
H	3.31133	-0.53243	14.55325	H	3.31133	-11.912	14.55325
C	4.17345	0.98035	13.28837	C	4.17345	-10.3993	13.28837
H	3.65197	0.63917	12.39531	H	3.65197	-10.7404	12.39531
C	5.03005	2.07279	13.21839	C	5.03005	-9.30681	13.21839
C	5.69569	2.50829	14.35821	C	5.69569	-8.87131	14.35821
H	6.36676	3.36419	14.30327	H	6.36676	-8.01542	14.30327
C	5.50472	1.85146	15.56824	C	5.50472	-9.52814	15.56824
H	6.02622	2.19264	16.4613	H	6.02622	-9.18696	16.4613
C	6.25982	1.59542	4.26278	C	6.25982	-9.78418	4.26278
H	5.75421	2.33043	3.63825	H	5.75421	-9.04917	3.63825
H	7.31994	1.57611	4.01448	H	7.31994	-9.80349	4.01448
H	5.82803	0.61124	4.08707	H	5.82803	-10.7684	4.08707
C	4.42651	0.05235	16.96894	C	4.42651	-11.3273	16.96894
H	5.02869	0.5318	17.73929	H	5.02869	-10.8478	17.73929
H	3.37381	0.11233	17.24124	H	3.37381	-11.2673	17.24124
H	4.71894	-0.99271	16.87805	H	4.71894	-12.3723	16.87805

Table S13: Cartesian coordinates of J_6 in pTolOV at 0 GPa.

	Molecule 1				Molecule 2		
	x	y	z		x	y	z
O	5.33508	1.02223	10.63424	O	9.15491	-1.02223	11.5112
N	5.60391	3.11619	9.7544	N	8.88608	-3.11619	12.39104
N	5.21523	4.19395	12.22251	N	9.27476	-4.19395	9.92293
N	5.1825	2.83773	12.01811	N	9.30749	-2.83773	10.12733
N	5.61123	4.47673	9.89635	N	8.87876	-4.47673	12.24908
C	5.41696	4.92293	11.13273	C	9.07304	-4.92293	11.0127
C	5.43702	6.40159	11.31565	C	9.05297	-6.40159	10.82978
C	5.82327	7.22559	10.26507	C	8.66672	-7.22559	11.88036
H	6.12189	6.78977	9.3128	H	8.36811	-6.78977	12.83264
C	5.82846	8.60571	10.42984	C	8.66153	-8.60571	11.7156
H	6.13106	9.25122	9.60662	H	8.35894	-9.25122	12.53881
C	5.44726	9.16206	11.64518	C	9.04274	-9.16206	10.50026
H	5.45134	10.24337	11.77428	H	9.03866	-10.2434	10.37115
C	5.06101	8.33817	12.69598	C	9.42898	-8.33817	9.44946
H	4.76237	8.77396	13.64825	H	9.72762	-8.77396	8.49718
C	5.05582	6.95794	12.53122	C	9.43418	-6.95794	9.61422
H	4.75321	6.31245	13.35443	H	9.73679	-6.31245	8.79101
C	5.75573	2.67341	8.39666	C	8.73426	-2.67341	13.74877
C	6.47992	1.53488	8.06293	C	8.01008	-1.53488	14.0825
H	6.91685	0.91817	8.84695	H	7.57315	-0.91817	13.29849
C	6.64637	1.18337	6.72845	C	7.84363	-1.18337	15.41699
H	7.21372	0.29137	6.46695	H	7.27627	-0.29137	15.67848
C	6.08864	1.97049	5.72769	C	8.40136	-1.97049	16.41774
C	5.36453	3.10902	6.06143	C	9.12546	-3.10902	16.08401
H	4.92758	3.72567	5.27737	H	9.56241	-3.72567	16.86807
C	5.19801	3.46054	7.39591	C	9.29199	-3.46054	14.74952
H	4.63067	4.35256	7.65734	H	9.85932	-4.35256	14.4881
C	5.37165	2.22039	10.78837	C	9.11834	-2.22039	11.35706
C	4.64805	0.75891	15.63822	C	9.84195	-0.75891	6.50721
C	3.98249	0.32341	14.49839	C	10.50751	-0.32341	7.64704
H	3.31133	-0.53243	14.55325	H	11.17867	0.53243	7.59218
C	4.17345	0.98035	13.28837	C	10.31654	-0.98035	8.85707
H	3.65197	0.63917	12.39531	H	10.83803	-0.63917	9.75013
C	5.03005	2.07279	13.21839	C	9.45994	-2.07279	8.92705
C	5.69569	2.50829	14.35821	C	8.79431	-2.50829	7.78722
H	6.36676	3.36419	14.30327	H	8.12324	-3.36419	7.84216
C	5.50472	1.85146	15.56824	C	8.98527	-1.85146	6.57719
H	6.02622	2.19264	16.4613	H	8.46377	-2.19264	5.68414
C	6.25982	1.59542	4.26278	C	8.23017	-1.59542	17.88266
H	5.75421	2.33043	3.63825	H	8.73578	-2.33043	18.50718
H	7.31994	1.57611	4.01448	H	7.17006	-1.57611	18.13095
H	5.82803	0.61124	4.08707	H	8.66196	-0.61124	18.05836
C	4.42651	0.05235	16.96894	C	10.06348	-0.05235	5.1765
H	5.02869	0.5318	17.73929	H	9.4613	-0.5318	4.40614
H	3.37381	0.11233	17.24124	H	11.11618	-0.11233	4.90419
H	4.71894	-0.99271	16.87805	H	9.77105	0.99271	5.26738

Table S14: Cartesian coordinates of J_7 in pTolOV at 0 GPa.

	Molecule 1				Molecule 2		
	x	y	z		x	y	z
O	5.33508	1.02223	10.63424	O	9.02193	4.66757	-0.43848
N	5.60391	3.11619	9.7544	N	9.29077	2.57361	-1.31832
N	5.21523	4.19395	12.22251	N	8.90209	1.49585	1.14979
N	5.1825	2.83773	12.01811	N	8.86936	2.85207	0.94539
N	5.61123	4.47673	9.89635	N	9.29809	1.21307	-1.17637
C	5.41696	4.92293	11.13273	C	9.10381	0.76687	0.06001
C	5.43702	6.40159	11.31565	C	9.12387	-0.71179	0.24294
C	5.82327	7.22559	10.26507	C	9.51012	-1.53579	-0.80764
H	6.12189	6.78977	9.3128	H	9.80874	-1.09997	-1.75992
C	5.82846	8.60571	10.42984	C	9.51531	-2.91591	-0.64288
H	6.13106	9.25122	9.60662	H	9.81791	-3.56142	-1.46609
C	5.44726	9.16206	11.64518	C	9.13411	-3.47226	0.57246
H	5.45134	10.24337	11.77428	H	9.13819	-4.55357	0.70157
C	5.06101	8.33817	12.69598	C	8.74786	-2.64837	1.62326
H	4.76237	8.77396	13.64825	H	8.44923	-3.08416	2.57554
C	5.05582	6.95794	12.53122	C	8.74267	-1.26814	1.4585
H	4.75321	6.31245	13.35443	H	8.44006	-0.62265	2.28171
C	5.75573	2.67341	8.39666	C	9.44259	3.01639	-2.67605
C	6.47992	1.53488	8.06293	C	10.16677	4.15492	-3.00979
H	6.91685	0.91817	8.84695	H	10.6037	4.77163	-2.22577
C	6.64637	1.18337	6.72845	C	10.33322	4.50643	-4.34427
H	7.21372	0.29137	6.46695	H	10.90058	5.39843	-4.60576
C	6.08864	1.97049	5.72769	C	9.77549	3.71931	-5.34502
C	5.36453	3.10902	6.06143	C	9.05138	2.58078	-5.01129
H	4.92758	3.72567	5.27737	H	8.61444	1.96413	-5.79535
C	5.19801	3.46054	7.39591	C	8.88486	2.22926	-3.67681
H	4.63067	4.35256	7.65734	H	8.31753	1.33724	-3.41538
C	5.37165	2.22039	10.78837	C	9.0585	3.46941	-0.28435
C	4.64805	0.75891	15.63822	C	8.3349	4.93089	4.5655
C	3.98249	0.32341	14.49839	C	7.66934	5.36639	3.42568
H	3.31133	-0.53243	14.55325	H	6.99818	6.22223	3.48053
C	4.17345	0.98035	13.28837	C	7.86031	4.70945	2.21565
H	3.65197	0.63917	12.39531	H	7.33882	5.05063	1.32259
C	5.03005	2.07279	13.21839	C	8.71691	3.61701	2.14567
C	5.69569	2.50829	14.35821	C	9.38254	3.18151	3.2855
H	6.36676	3.36419	14.30327	H	10.05361	2.32562	3.23055
C	5.50472	1.85146	15.56824	C	9.19157	3.83834	4.49552
H	6.02622	2.19264	16.4613	H	9.71307	3.49716	5.38858
C	6.25982	1.59542	4.26278	C	9.94668	4.09438	-6.80994
H	5.75421	2.33043	3.63825	H	9.44106	3.35937	-7.43447
H	7.31994	1.57611	4.01448	H	11.00679	4.11369	-7.05824
H	5.82803	0.61124	4.08707	H	9.51488	5.07856	-6.98564
C	4.42651	0.05235	16.96894	C	8.11337	5.63745	5.89622
H	5.02869	0.5318	17.73929	H	8.71555	5.158	6.66657
H	3.37381	0.11233	17.24124	H	7.06067	5.57747	6.16852
H	4.71894	-0.99271	16.87805	H	8.40579	6.68251	5.80534

Table S15: Cartesian coordinates of J_8 in pTolOV at 0 GPa.

	Molecule 1				Molecule 2		
	x	y	z		x	y	z
O	5.33508	1.02223	10.63424	O	5.55387	-4.66757	0.43848
N	5.60391	3.11619	9.7544	N	5.28503	-2.57361	1.31832
N	5.21523	4.19395	12.22251	N	5.67371	-1.49585	-1.14979
N	5.1825	2.83773	12.01811	N	5.70645	-2.85207	-0.94539
N	5.61123	4.47673	9.89635	N	5.27771	-1.21307	1.17637
C	5.41696	4.92293	11.13273	C	5.47199	-0.76687	-0.06001
C	5.43702	6.40159	11.31565	C	5.45193	0.71179	-0.24294
C	5.82327	7.22559	10.26507	C	5.06568	1.53579	0.80764
H	6.12189	6.78977	9.3128	H	4.76706	1.09997	1.75992
C	5.82846	8.60571	10.42984	C	5.06049	2.91591	0.64288
H	6.13106	9.25122	9.60662	H	4.75789	3.56142	1.46609
C	5.44726	9.16206	11.64518	C	5.44169	3.47226	-0.57246
H	5.45134	10.24337	11.77428	H	5.43761	4.55357	-0.70157
C	5.06101	8.33817	12.69598	C	5.82794	2.64837	-1.62326
H	4.76237	8.77396	13.64825	H	6.12657	3.08416	-2.57554
C	5.05582	6.95794	12.53122	C	5.83313	1.26814	-1.4585
H	4.75321	6.31245	13.35443	H	6.13574	0.62265	-2.28171
C	5.75573	2.67341	8.39666	C	5.13321	-3.01639	2.67605
C	6.47992	1.53488	8.06293	C	4.40903	-4.15492	3.00979
H	6.91685	0.91817	8.84695	H	3.9721	-4.77163	2.22577
C	6.64637	1.18337	6.72845	C	4.24258	-4.50643	4.34427
H	7.21372	0.29137	6.46695	H	3.67523	-5.39843	4.60576
C	6.08864	1.97049	5.72769	C	4.80031	-3.71931	5.34502
C	5.36453	3.10902	6.06143	C	5.52442	-2.58078	5.01129
H	4.92758	3.72567	5.27737	H	5.96136	-1.96413	5.79535
C	5.19801	3.46054	7.39591	C	5.69094	-2.22926	3.67681
H	4.63067	4.35256	7.65734	H	6.25827	-1.33724	3.41538
C	5.37165	2.22039	10.78837	C	5.5173	-3.46941	0.28435
C	4.64805	0.75891	15.63822	C	6.2409	-4.93089	-4.5655
C	3.98249	0.32341	14.49839	C	6.90646	-5.36639	-3.42568
H	3.31133	-0.53243	14.55325	H	7.57762	-6.22223	-3.48053
C	4.17345	0.98035	13.28837	C	6.71549	-4.70945	-2.21565
H	3.65197	0.63917	12.39531	H	7.23698	-5.05063	-1.32259
C	5.03005	2.07279	13.21839	C	5.85889	-3.61701	-2.14567
C	5.69569	2.50829	14.35821	C	5.19326	-3.18151	-3.2855
H	6.36676	3.36419	14.30327	H	4.52219	-2.32562	-3.23055
C	5.50472	1.85146	15.56824	C	5.38423	-3.83834	-4.49552
H	6.02622	2.19264	16.4613	H	4.86273	-3.49716	-5.38858
C	6.25982	1.59542	4.26278	C	4.62912	-4.09438	6.80994
H	5.75421	2.33043	3.63825	H	5.13474	-3.35937	7.43447
H	7.31994	1.57611	4.01448	H	3.56901	-4.11369	7.05824
H	5.82803	0.61124	4.08707	H	5.06091	-5.07856	6.98564
C	4.42651	0.05235	16.96894	C	6.46243	-5.63745	-5.89622
H	5.02869	0.5318	17.73929	H	5.86025	-5.158	-6.66657
H	3.37381	0.11233	17.24124	H	7.51513	-5.57747	-6.16852
H	4.71894	-0.99271	16.87805	H	6.17	-6.68251	-5.80534

Table S16: Cartesian coordinates of J_9 in pTolOV at 0 GPa.

	Molecule 1				Molecule 2		
	x	y	z		x	y	z
O	5.33508	1.022229	10.63424	O	8.936127	4.667571	21.70695
N	5.603914	3.11619	9.754399	N	9.204961	2.57361	20.82712
N	5.215235	4.193952	12.22251	N	8.816282	1.495848	23.29523
N	5.182502	2.837731	12.01811	N	8.783549	2.852069	23.09082
N	5.611235	4.476735	9.896351	N	9.212282	1.213065	20.96907
C	5.416959	4.922929	11.13273	C	9.018006	0.766871	22.20545
C	5.437021	6.401594	11.31565	C	9.038068	-0.71179	22.38837
C	5.823269	7.225591	10.26507	C	9.424316	-1.53579	21.33779
H	6.121886	6.789775	9.312797	H	9.722933	-1.09998	20.38551
C	5.828461	8.605709	10.42984	C	9.429508	-2.91591	21.50255
H	6.131059	9.251217	9.606623	H	9.732107	-3.56142	20.67934
C	5.447259	9.162057	11.64518	C	9.048306	-3.47226	22.71789
H	5.451336	10.24337	11.77428	H	9.052383	-4.55357	22.847
C	5.06101	8.338174	12.69598	C	8.662057	-2.64837	23.76869
H	4.762372	8.773956	13.64825	H	8.363419	-3.08416	24.72097
C	5.055818	6.957943	12.53122	C	8.656865	-1.26814	23.60393
H	4.753205	6.312446	13.35443	H	8.354252	-0.62265	24.42714
C	5.755735	2.673409	8.396663	C	9.356782	3.016391	19.46938
C	6.479915	1.53488	8.062931	C	10.08096	4.15492	19.13565
H	6.916847	0.918169	8.846946	H	10.51789	4.771631	19.91966
C	6.646366	1.183365	6.728447	C	10.24741	4.506435	17.80116
H	7.213722	0.291372	6.466954	H	10.81477	5.398428	17.53967
C	6.088638	1.970492	5.727695	C	9.689686	3.719308	16.80041
C	5.364531	3.109021	6.061427	C	8.965579	2.580779	17.13414
H	4.927584	3.72567	5.277368	H	8.528631	1.96413	16.35008
C	5.198007	3.460536	7.395911	C	8.799054	2.229264	18.46863
H	4.630673	4.35256	7.657337	H	8.23172	1.33724	18.73005
C	5.371651	2.220388	10.78837	C	8.972698	3.469412	21.86109
C	4.648047	0.758906	15.63822	C	8.249094	4.930894	26.71094
C	3.982486	0.323408	14.49839	C	7.583534	5.366392	25.57111
H	3.311328	-0.53243	14.55325	H	6.912375	6.222233	25.62597
C	4.173454	0.980353	13.28837	C	7.774501	4.709447	24.36108
H	3.651966	0.639173	12.39531	H	7.253014	5.050627	23.46803
C	5.030053	2.072794	13.21839	C	8.6311	3.617006	24.29111
C	5.695686	2.508291	14.35821	C	9.296733	3.181509	25.43093
H	6.366757	3.364185	14.30327	H	9.967805	2.325615	25.37599
C	5.504719	1.851461	15.56824	C	9.105766	3.838339	26.64096
H	6.026221	2.192644	16.4613	H	9.627268	3.497156	27.53402
C	6.259823	1.59542	4.262775	C	9.86087	4.09438	15.33549
H	5.75421	2.330428	3.638251	H	9.355257	3.359372	14.71097
H	7.319935	1.576109	4.01448	H	10.92098	4.113691	15.0872
H	5.828032	0.61124	4.087073	H	9.429079	5.07856	15.15979
C	4.426513	0.052346	16.96894	C	8.02756	5.637454	28.04166
H	5.028693	0.531796	17.73929	H	8.62974	5.158004	28.81201
H	3.373814	0.112328	17.24124	H	6.974861	5.577472	28.31396
H	4.718942	-0.99271	16.87805	H	8.319989	6.682513	27.95077

Table S17: Cartesian coordinates of J_{10} in pTolOV at 0 GPa.

	Molecule 1				Molecule 2		
	x	y	z		x	y	z
O	5.33508	1.02223	10.63424	O	8.93613	4.66757	21.70695
N	5.60391	3.11619	9.7544	N	9.20496	2.57361	20.82712
N	5.21523	4.19395	12.22251	N	8.81628	1.49585	23.29522
N	5.1825	2.83773	12.01811	N	8.78355	2.85207	23.09082
N	5.61123	4.47673	9.89635	N	9.21228	1.21307	20.96907
C	5.41696	4.92293	11.13273	C	9.01801	0.76687	22.20545
C	5.43702	6.40159	11.31565	C	9.03807	-0.71179	22.38837
C	5.82327	7.22559	10.26507	C	9.42432	-1.53579	21.33779
H	6.12189	6.78977	9.3128	H	9.72293	-1.09997	20.38551
C	5.82846	8.60571	10.42984	C	9.42951	-2.91591	21.50255
H	6.13106	9.25122	9.60662	H	9.73211	-3.56142	20.67934
C	5.44726	9.16206	11.64518	C	9.04831	-3.47226	22.71789
H	5.45134	10.24337	11.77428	H	9.05238	-4.55357	22.847
C	5.06101	8.33817	12.69598	C	8.66206	-2.64837	23.76869
H	4.76237	8.77396	13.64825	H	8.36342	-3.08416	24.72097
C	5.05582	6.95794	12.53122	C	8.65686	-1.26814	23.60393
H	4.75321	6.31245	13.35443	H	8.35425	-0.62265	24.42714
C	5.75573	2.67341	8.39666	C	9.35678	3.01639	19.46938
C	6.47992	1.53488	8.06293	C	10.08096	4.15492	19.13565
H	6.91685	0.91817	8.84695	H	10.51789	4.77163	19.91966
C	6.64637	1.18337	6.72845	C	10.24741	4.50643	17.80116
H	7.21372	0.29137	6.46695	H	10.81477	5.39843	17.53967
C	6.08864	1.97049	5.72769	C	9.68969	3.71931	16.80041
C	5.36453	3.10902	6.06143	C	8.96558	2.58078	17.13414
H	4.92758	3.72567	5.27737	H	8.52863	1.96413	16.35008
C	5.19801	3.46054	7.39591	C	8.79905	2.22926	18.46863
H	4.63067	4.35256	7.65734	H	8.23172	1.33724	18.73005
C	5.37165	2.22039	10.78837	C	8.9727	3.46941	21.86109
C	4.64805	0.75891	15.63822	C	8.24909	4.93089	26.71094
C	3.98249	0.32341	14.49839	C	7.58353	5.36639	25.57111
H	3.31133	-0.53243	14.55325	H	6.91237	6.22223	25.62597
C	4.17345	0.98035	13.28837	C	7.7745	4.70945	24.36108
H	3.65197	0.63917	12.39531	H	7.25301	5.05063	23.46803
C	5.03005	2.07279	13.21839	C	8.6311	3.61701	24.29111
C	5.69569	2.50829	14.35821	C	9.29673	3.18151	25.43093
H	6.36676	3.36419	14.30327	H	9.96781	2.32562	25.37599
C	5.50472	1.85146	15.56824	C	9.10577	3.83834	26.64096
H	6.02622	2.19264	16.4613	H	9.62727	3.49716	27.53402
C	6.25982	1.59542	4.26278	C	9.86087	4.09438	15.33549
H	5.75421	2.33043	3.63825	H	9.35526	3.35937	14.71097
H	7.31994	1.57611	4.01448	H	10.92098	4.11369	15.0872
H	5.82803	0.61124	4.08707	H	9.42908	5.07856	15.15979
C	4.42651	0.05235	16.96894	C	8.02756	5.63745	28.04166
H	5.02869	0.5318	17.73929	H	8.62974	5.158	28.81201
H	3.37381	0.11233	17.24124	H	6.97486	5.57747	28.31396
H	4.71894	-0.99271	16.87805	H	8.31999	6.68251	27.95077

Table S18: Cartesian coordinates of J_{11} in pTolOV at 0 GPa.

Molecule 1				Molecule 2			
	x	y	z		x	y	z
O	5.33508	1.02223	10.63424	O	5.46806	-4.66757	22.58391
N	5.60391	3.11619	9.7544	N	5.19923	-2.57361	23.46375
N	5.21523	4.19395	12.22251	N	5.58791	-1.49585	20.99564
N	5.1825	2.83773	12.01811	N	5.62064	-2.85207	21.20004
N	5.61123	4.47673	9.89635	N	5.19191	-1.21307	23.3218
C	5.41696	4.92293	11.13273	C	5.38618	-0.76687	22.08542
C	5.43702	6.40159	11.31565	C	5.36612	0.71179	21.9025
C	5.82327	7.22559	10.26507	C	4.97987	1.53579	22.95308
H	6.12189	6.78977	9.3128	H	4.68126	1.09997	23.90535
C	5.82846	8.60571	10.42984	C	4.97468	2.91591	22.78832
H	6.13106	9.25122	9.60662	H	4.67208	3.56142	23.61153
C	5.44726	9.16206	11.64518	C	5.35588	3.47226	21.57297
H	5.45134	10.24337	11.77428	H	5.35181	4.55357	21.44387
C	5.06101	8.33817	12.69598	C	5.74213	2.64837	20.52217
H	4.76237	8.77396	13.64825	H	6.04077	3.08416	19.5699
C	5.05582	6.95794	12.53122	C	5.74732	1.26814	20.68693
H	4.75321	6.31245	13.35443	H	6.04994	0.62265	19.86372
C	5.75573	2.67341	8.39666	C	5.04741	-3.01639	24.82149
C	6.47992	1.53488	8.06293	C	4.32323	-4.15492	25.15522
H	6.91685	0.91817	8.84695	H	3.88629	-4.77163	24.37121
C	6.64637	1.18337	6.72845	C	4.15677	-4.50643	26.4897
H	7.21372	0.29137	6.46695	H	3.58942	-5.39843	26.7512
C	6.08864	1.97049	5.72769	C	4.7145	-3.71931	27.49046
C	5.36453	3.10902	6.06143	C	5.43861	-2.58078	27.15672
H	4.92758	3.72567	5.27737	H	5.87556	-1.96413	27.94078
C	5.19801	3.46054	7.39591	C	5.60513	-2.22926	25.82224
H	4.63067	4.35256	7.65734	H	6.17247	-1.33724	25.56081
C	5.37165	2.22039	10.78837	C	5.43149	-3.46941	22.42978
C	4.64805	0.75891	15.63822	C	6.1551	-4.93089	17.57993
C	3.98249	0.32341	14.49839	C	6.82066	-5.36639	18.71976
H	3.31133	-0.53243	14.55325	H	7.49181	-6.22223	18.6649
C	4.17345	0.98035	13.28837	C	6.62969	-4.70945	19.92978
H	3.65197	0.63917	12.39531	H	7.15118	-5.05063	20.82284
C	5.03005	2.07279	13.21839	C	5.77309	-3.61701	19.99976
C	5.69569	2.50829	14.35821	C	5.10745	-3.18151	18.85994
H	6.36676	3.36419	14.30327	H	4.43638	-2.32562	18.91488
C	5.50472	1.85146	15.56824	C	5.29842	-3.83834	17.64991
H	6.02622	2.19264	16.4613	H	4.77692	-3.49716	16.75685
C	6.25982	1.59542	4.26278	C	4.54332	-4.09438	28.95538
H	5.75421	2.33043	3.63825	H	5.04893	-3.35937	29.5799
H	7.31994	1.57611	4.01448	H	3.48321	-4.11369	29.20367
H	5.82803	0.61124	4.08707	H	4.97511	-5.07856	29.13108
C	4.42651	0.05235	16.96894	C	6.37663	-5.63745	16.24921
H	5.02869	0.5318	17.73929	H	5.77445	-5.158	15.47886
H	3.37381	0.11233	17.24124	H	7.42933	-5.57747	15.97691
H	4.71894	-0.99271	16.87805	H	6.0842	-6.68251	16.3401

Table S19: Cartesian coordinates of J_{12} in pTolOV at 0 GPa.

	Molecule 1				Molecule 2		
	x	y	z		x	y	z
O	5.33508	1.02223	10.63424	O	1.64823	4.66757	21.70695
N	5.60391	3.11619	9.7544	N	1.91706	2.57361	20.82712
N	5.21523	4.19395	12.22251	N	1.52838	1.49585	23.29522
N	5.1825	2.83773	12.01811	N	1.49565	2.85207	23.09082
N	5.61123	4.47673	9.89635	N	1.92438	1.21307	20.96907
C	5.41696	4.92293	11.13273	C	1.73011	0.76687	22.20545
C	5.43702	6.40159	11.31565	C	1.75017	-0.71179	22.38837
C	5.82327	7.22559	10.26507	C	2.13642	-1.53579	21.33779
H	6.12189	6.78977	9.3128	H	2.43503	-1.09997	20.38551
C	5.82846	8.60571	10.42984	C	2.14161	-2.91591	21.50255
H	6.13106	9.25122	9.60662	H	2.44421	-3.56142	20.67934
C	5.44726	9.16206	11.64518	C	1.76041	-3.47226	22.71789
H	5.45134	10.24337	11.77428	H	1.76448	-4.55357	22.847
C	5.06101	8.33817	12.69598	C	1.37416	-2.64837	23.76869
H	4.76237	8.77396	13.64825	H	1.07552	-3.08416	24.72097
C	5.05582	6.95794	12.53122	C	1.36897	-1.26814	23.60393
H	4.75321	6.31245	13.35443	H	1.06635	-0.62265	24.42714
C	5.75573	2.67341	8.39666	C	2.06888	3.01639	19.46938
C	6.47992	1.53488	8.06293	C	2.79306	4.15492	19.13565
H	6.91685	0.91817	8.84695	H	3.22999	4.77163	19.91966
C	6.64637	1.18337	6.72845	C	2.95951	4.50643	17.80116
H	7.21372	0.29137	6.46695	H	3.52687	5.39843	17.53967
C	6.08864	1.97049	5.72769	C	2.40179	3.71931	16.80041
C	5.36453	3.10902	6.06143	C	1.67768	2.58078	17.13414
H	4.92758	3.72567	5.27737	H	1.24073	1.96413	16.35008
C	5.19801	3.46054	7.39591	C	1.51115	2.22926	18.46863
H	4.63067	4.35256	7.65734	H	0.94382	1.33724	18.73005
C	5.37165	2.22039	10.78837	C	1.6848	3.46941	21.86109
C	4.64805	0.75891	15.63822	C	0.96119	4.93089	26.71094
C	3.98249	0.32341	14.49839	C	0.29563	5.36639	25.57111
H	3.31133	-0.53243	14.55325	H	-0.37553	6.22223	25.62597
C	4.17345	0.98035	13.28837	C	0.4866	4.70945	24.36108
H	3.65197	0.63917	12.39531	H	-0.03489	5.05063	23.46803
C	5.03005	2.07279	13.21839	C	1.3432	3.61701	24.29111
C	5.69569	2.50829	14.35821	C	2.00883	3.18151	25.43093
H	6.36676	3.36419	14.30327	H	2.67991	2.32562	25.37599
C	5.50472	1.85146	15.56824	C	1.81787	3.83834	26.64096
H	6.02622	2.19264	16.4613	H	2.33937	3.49716	27.53402
C	6.25982	1.59542	4.26278	C	2.57297	4.09438	15.33549
H	5.75421	2.33043	3.63825	H	2.06736	3.35937	14.71097
H	7.31994	1.57611	4.01448	H	3.63308	4.11369	15.0872
H	5.82803	0.61124	4.08707	H	2.14118	5.07856	15.15979
C	4.42651	0.05235	16.96894	C	0.73966	5.63745	28.04166
H	5.02869	0.5318	17.73929	H	1.34184	5.158	28.81201
H	3.37381	0.11233	17.24124	H	-0.31304	5.57747	28.31396
H	4.71894	-0.99271	16.87805	H	1.03209	6.68251	27.95077

Table S20: Cartesian coordinates of J_{13} in pTolOV at 0 GPa.

	Molecule 1				Molecule 2		
	x	y	z		x	y	z
O	5.33508	1.02223	10.63424	O	5.46806	6.71203	22.58391
N	5.60391	3.11619	9.7544	N	5.19923	8.80599	23.46375
N	5.21523	4.19395	12.22251	N	5.58791	9.88375	20.99564
N	5.1825	2.83773	12.01811	N	5.62064	8.52753	21.20004
N	5.61123	4.47673	9.89635	N	5.19191	10.16654	23.3218
C	5.41696	4.92293	11.13273	C	5.38618	10.61273	22.08542
C	5.43702	6.40159	11.31565	C	5.36612	12.09139	21.9025
C	5.82327	7.22559	10.26507	C	4.97987	12.91539	22.95308
H	6.12189	6.78977	9.3128	H	4.68126	12.47958	23.90535
C	5.82846	8.60571	10.42984	C	4.97468	14.29551	22.78832
H	6.13106	9.25122	9.60662	H	4.67208	14.94102	23.61153
C	5.44726	9.16206	11.64518	C	5.35588	14.85186	21.57297
H	5.45134	10.24337	11.77428	H	5.35181	15.93317	21.44387
C	5.06101	8.33817	12.69598	C	5.74213	14.02797	20.52217
H	4.76237	8.77396	13.64825	H	6.04077	14.46376	19.5699
C	5.05582	6.95794	12.53122	C	5.74732	12.64774	20.68693
H	4.75321	6.31245	13.35443	H	6.04994	12.00225	19.86372
C	5.75573	2.67341	8.39666	C	5.04741	8.36321	24.82149
C	6.47992	1.53488	8.06293	C	4.32323	7.22468	25.15522
H	6.91685	0.91817	8.84695	H	3.88629	6.60797	24.37121
C	6.64637	1.18337	6.72845	C	4.15677	6.87317	26.4897
H	7.21372	0.29137	6.46695	H	3.58942	5.98117	26.7512
C	6.08864	1.97049	5.72769	C	4.7145	7.66029	27.49046
C	5.36453	3.10902	6.06143	C	5.43861	8.79882	27.15672
H	4.92758	3.72567	5.27737	H	5.87556	9.41547	27.94078
C	5.19801	3.46054	7.39591	C	5.60513	9.15034	25.82224
H	4.63067	4.35256	7.65734	H	6.17247	10.04236	25.56081
C	5.37165	2.22039	10.78837	C	5.43149	7.91019	22.42978
C	4.64805	0.75891	15.63822	C	6.1551	6.44871	17.57993
C	3.98249	0.32341	14.49839	C	6.82066	6.01321	18.71976
H	3.31133	-0.53243	14.55325	H	7.49181	5.15737	18.6649
C	4.17345	0.98035	13.28837	C	6.62969	6.67015	19.92978
H	3.65197	0.63917	12.39531	H	7.15118	6.32897	20.82284
C	5.03005	2.07279	13.21839	C	5.77309	7.76259	19.99976
C	5.69569	2.50829	14.35821	C	5.10745	8.19809	18.85994
H	6.36676	3.36419	14.30327	H	4.43638	9.05399	18.91488
C	5.50472	1.85146	15.56824	C	5.29842	7.54126	17.64991
H	6.02622	2.19264	16.4613	H	4.77692	7.88244	16.75685
C	6.25982	1.59542	4.26278	C	4.54332	7.28522	28.95538
H	5.75421	2.33043	3.63825	H	5.04893	8.02023	29.5799
H	7.31994	1.57611	4.01448	H	3.48321	7.26591	29.20367
H	5.82803	0.61124	4.08707	H	4.97511	6.30104	29.13108
C	4.42651	0.05235	16.96894	C	6.37663	5.74215	16.24921
H	5.02869	0.5318	17.73929	H	5.77445	6.2216	15.47886
H	3.37381	0.11233	17.24124	H	7.42933	5.80213	15.97691
H	4.71894	-0.99271	16.87805	H	6.0842	4.69709	16.3401

Table S21: Cartesian coordinates of J_{14} in pTolOV at 0 GPa.

	Molecule 1				Molecule 2		
	x	y	z		x	y	z
O	5.33508	1.02223	10.63424	O	5.55387	6.71203	0.43848
N	5.60391	3.11619	9.7544	N	5.28503	8.80599	1.31832
N	5.21523	4.19395	12.22251	N	5.67371	9.88375	-1.14979
N	5.1825	2.83773	12.01811	N	5.70645	8.52753	-0.94539
N	5.61123	4.47673	9.89635	N	5.27771	10.16654	1.17637
C	5.41696	4.92293	11.13273	C	5.47199	10.61273	-0.06001
C	5.43702	6.40159	11.31565	C	5.45193	12.09139	-0.24294
C	5.82327	7.22559	10.26507	C	5.06568	12.91539	0.80764
H	6.12189	6.78977	9.3128	H	4.76706	12.47958	1.75992
C	5.82846	8.60571	10.42984	C	5.06049	14.29551	0.64288
H	6.13106	9.25122	9.60662	H	4.75789	14.94102	1.46609
C	5.44726	9.16206	11.64518	C	5.44169	14.85186	-0.57246
H	5.45134	10.24337	11.77428	H	5.43761	15.93317	-0.70157
C	5.06101	8.33817	12.69598	C	5.82794	14.02797	-1.62326
H	4.76237	8.77396	13.64825	H	6.12657	14.46376	-2.57554
C	5.05582	6.95794	12.53122	C	5.83313	12.64774	-1.4585
H	4.75321	6.31245	13.35443	H	6.13574	12.00225	-2.28171
C	5.75573	2.67341	8.39666	C	5.13321	8.36321	2.67605
C	6.47992	1.53488	8.06293	C	4.40903	7.22468	3.00979
H	6.91685	0.91817	8.84695	H	3.9721	6.60797	2.22577
C	6.64637	1.18337	6.72845	C	4.24258	6.87317	4.34427
H	7.21372	0.29137	6.46695	H	3.67523	5.98117	4.60576
C	6.08864	1.97049	5.72769	C	4.80031	7.66029	5.34502
C	5.36453	3.10902	6.06143	C	5.52442	8.79882	5.01129
H	4.92758	3.72567	5.27737	H	5.96136	9.41547	5.79535
C	5.19801	3.46054	7.39591	C	5.69094	9.15034	3.67681
H	4.63067	4.35256	7.65734	H	6.25827	10.04236	3.41538
C	5.37165	2.22039	10.78837	C	5.5173	7.91019	0.28435
C	4.64805	0.75891	15.63822	C	6.2409	6.44871	-4.5655
C	3.98249	0.32341	14.49839	C	6.90646	6.01321	-3.42568
H	3.31133	-0.53243	14.55325	H	7.57762	5.15737	-3.48053
C	4.17345	0.98035	13.28837	C	6.71549	6.67015	-2.21565
H	3.65197	0.63917	12.39531	H	7.23698	6.32897	-1.32259
C	5.03005	2.07279	13.21839	C	5.85889	7.76259	-2.14567
C	5.69569	2.50829	14.35821	C	5.19326	8.19809	-3.2855
H	6.36676	3.36419	14.30327	H	4.52219	9.05399	-3.23055
C	5.50472	1.85146	15.56824	C	5.38423	7.54126	-4.49552
H	6.02622	2.19264	16.4613	H	4.86273	7.88244	-5.38858
C	6.25982	1.59542	4.26278	C	4.62912	7.28522	6.80994
H	5.75421	2.33043	3.63825	H	5.13474	8.02023	7.43447
H	7.31994	1.57611	4.01448	H	3.56901	7.26591	7.05824
H	5.82803	0.61124	4.08707	H	5.06091	6.30104	6.98564
C	4.42651	0.05235	16.96894	C	6.46243	5.74215	-5.89622
H	5.02869	0.5318	17.73929	H	5.86025	6.2216	-6.66657
H	3.37381	0.11233	17.24124	H	7.51513	5.80213	-6.16852
H	4.71894	-0.99271	16.87805	H	6.17	4.69709	-5.80534

S8.2 2.73 GPa

Table S22: Cartesian coordinates of J_1 in pTolOV at 2.73 GPa.

	Molecule 1				Molecule 2		
	x	y	z		x	y	z
N	-2.03860	-2.86280	8.94330	C	-1.60360	-1.29520	5.75320
N	-2.06590	-1.47040	8.90220	H	-2.42800	-0.74980	6.19600
C	-3.25220	-3.54770	9.12990	C	-1.64360	-2.69440	5.73930
C	-4.38370	-2.96230	8.57550	H	-2.50310	-3.24150	6.10650
H	-4.28780	-2.11290	7.91050	N	-1.67990	2.86280	5.46070
C	-1.96230	1.29520	9.23580	N	-1.70720	1.47040	5.41960
C	-2.00220	2.69440	9.22190	C	-6.79290	5.10170	6.57300
H	-2.86180	3.24150	9.58910	H	-7.09560	5.80810	5.80990
H	2.93500	-5.98280	6.47290	H	-7.50410	4.28580	6.61130
O	-0.89670	-4.84400	8.70640	C	-2.89360	3.54770	5.64740
N	0.24530	-2.86280	8.46960	C	-4.02500	2.96230	5.09290
N	0.27260	-1.47040	8.51070	H	-3.92910	2.11290	4.42790
C	-0.89670	-0.89960	8.70640	C	-5.27020	3.46970	5.39450
C	-0.89670	-3.61550	8.70640	H	-6.14800	3.00800	4.95940
C	1.45890	-3.54770	8.28300	C	-5.42680	4.56040	6.24500
C	2.84400	-5.13450	7.14000	C	-4.27870	5.13450	6.79030
C	1.59310	-4.63380	7.43950	C	-3.02780	4.63380	6.49080
H	0.71210	-5.09520	7.01090	H	-2.14670	5.09520	6.91940
C	-0.89670	0.59450	8.70640	H	-6.76350	5.59880	7.53480
C	0.16900	1.29520	8.17710	H	-4.36960	5.98280	7.45740
H	0.99330	0.74980	7.73430	C	-0.53800	-0.59450	5.22390
C	0.20890	2.69440	8.19100	C	0.52760	-1.29520	4.69450
H	1.06850	3.24150	7.82380	H	1.35200	-0.74980	4.25170
C	-0.89670	3.35100	8.70640	C	0.56760	-2.69440	4.70840
H	-0.89670	4.43400	8.70640	H	1.42710	-3.24150	4.34120
C	-3.38640	-4.63380	9.97340	C	-0.53800	-3.35100	5.22390
C	-5.62890	-3.46970	8.87710	H	-0.53800	-4.43400	5.22390
H	-2.78660	0.74980	9.67860	O	-0.53800	4.84400	5.22390
C	2.59040	-2.96230	8.83740	N	0.60400	2.86280	4.98710
C	3.99210	-4.56040	7.68540	N	0.63130	1.47040	5.02810
C	-4.63730	-5.13450	10.27290	C	-0.53800	0.89960	5.22390
H	-2.50540	-5.09520	10.40200	C	-0.53800	3.61550	5.22390
H	-6.50670	-3.00800	8.44200	C	5.71700	5.10170	3.87470
C	-5.78540	-4.56040	9.72750	H	6.01960	5.80810	4.63780
H	2.49450	-2.11290	9.50240	H	5.68750	5.59880	2.91300
C	3.83560	-3.46970	8.53580	H	6.42810	4.28580	3.83640
C	5.35830	-5.10170	7.35730	C	1.81760	3.54770	4.80040
H	-4.72830	-5.98280	10.94000	C	2.94900	2.96230	5.35480
C	-7.15160	-5.10170	10.05560	H	2.85310	2.11290	6.01980
H	4.71340	-3.00800	8.97090	C	4.19420	3.46970	5.05320
H	5.66090	-5.80810	8.12040	H	5.07200	3.00800	5.48830
H	5.32880	-5.59880	6.39550	C	4.35080	4.56040	4.20280
H	6.06940	-4.28580	7.31900	C	3.20270	5.13450	3.65740
H	-7.45420	-5.80810	9.29250	H	3.29360	5.98280	2.99040
H	-7.12210	-5.59880	11.01740	C	1.95180	4.63380	3.95690
H	-7.86280	-4.28580	10.09390	H	1.07070	5.09520	3.52830

Table S23: Cartesian coordinates of J_2 in pTolOV at 2.73 GPa.

	Molecule 1				Molecule 2		
	x	y	z		x	y	z
C	-1.60360	-1.29520	5.75320	C	-4.27870	-6.16750	6.79030
H	-2.42800	-0.74980	6.19600	C	-3.02780	-6.66820	6.49080
C	-1.64360	-2.69440	5.73930	H	-2.14670	-6.20680	6.91940
H	-2.50310	-3.24150	6.10650	H	-4.36960	-5.31920	7.45740
N	-1.67990	2.86280	5.46070	O	-0.53800	-6.45800	5.22390
N	-1.70720	1.47040	5.41960	C	-0.53800	-7.68650	5.22390
C	-6.79290	5.10170	6.57300	C	3.20270	-6.16750	3.65740
H	-7.09560	5.80810	5.80990	H	3.29360	-5.31920	2.99040
H	-7.50410	4.28580	6.61130	C	1.95180	-6.66820	3.95690
C	-2.89360	3.54770	5.64740	H	1.07070	-6.20680	3.52830
C	-4.02500	2.96230	5.09290	C	-5.42680	-6.74160	6.24500
H	-3.92910	2.11290	4.42790	C	-2.89360	-7.75430	5.64740
C	-5.27020	3.46970	5.39450	N	0.60400	-8.43920	4.98710
H	-6.14800	3.00800	4.95940	N	-1.67990	-8.43920	5.46070
C	-5.42680	4.56040	6.24500	C	4.35080	-6.74160	4.20280
C	-4.27870	5.13450	6.79030	C	1.81760	-7.75430	4.80040
C	-3.02780	4.63380	6.49080	C	-6.79290	-6.20030	6.57300
H	-2.14670	5.09520	6.91940	C	-5.27020	-7.83230	5.39450
H	-6.76350	5.59880	7.53480	C	-4.02500	-8.33970	5.09290
H	-4.36960	5.98280	7.45740	N	0.63130	-9.83160	5.02810
C	-0.53800	-0.59450	5.22390	N	-1.70720	-9.83160	5.41960
C	0.52760	-1.29520	4.69450	C	5.71700	-6.20030	3.87470
H	1.35200	-0.74980	4.25170	C	4.19420	-7.83230	5.05320
C	0.56760	-2.69440	4.70840	C	2.94900	-8.33970	5.35480
H	1.42710	-3.24150	4.34120	H	-7.09560	-5.49390	5.80990
C	-0.53800	-3.35100	5.22390	H	-6.76350	-5.70320	7.53480
H	-0.53800	-4.43400	5.22390	H	-7.50410	-7.01620	6.61130
O	-0.53800	4.84400	5.22390	H	-6.14800	-8.29400	4.95940
N	0.60400	2.86280	4.98710	H	-3.92910	-9.18910	4.42790
N	0.63130	1.47040	5.02810	C	-0.53800	-10.40240	5.22390
C	-0.53800	0.89960	5.22390	H	6.01960	-5.49390	4.63780
C	-0.53800	3.61550	5.22390	H	5.68750	-5.70320	2.91300
C	5.71700	5.10170	3.87470	H	6.42810	-7.01620	3.83640
H	6.01960	5.80810	4.63780	H	5.07200	-8.29400	5.48830
H	5.68750	5.59880	2.91300	H	2.85310	-9.18910	6.01980
H	6.42810	4.28580	3.83640	C	-0.53800	-11.89650	5.22390
C	1.81760	3.54770	4.80040	C	-1.60360	-12.59720	5.75320
C	2.94900	2.96230	5.35480	C	0.52760	-12.59720	4.69450
H	2.85310	2.11290	6.01980	H	-2.42800	-12.05180	6.19600
C	4.19420	3.46970	5.05320	C	-1.64360	-13.99640	5.73930
H	5.07200	3.00800	5.48830	H	1.35200	-12.05180	4.25170
C	4.35080	4.56040	4.20280	C	0.56760	-13.99640	4.70840
C	3.20270	5.13450	3.65740	H	-2.50310	-14.54350	6.10650
H	3.29360	5.98280	2.99040	C	-0.53800	-14.65300	5.22390
C	1.95180	4.63380	3.95690	H	1.42710	-14.54350	4.34120
H	1.07070	5.09520	3.52830	H	-0.53800	-15.73600	5.22390

Table S24: Cartesian coordinates of J_3 in pTolOV at 2.73 GPa.

	Molecule 1				Molecule 2		
	x	y	z		x	y	z
C	-1.60360	-1.29520	5.75320	N	-1.32130	-2.86280	1.97810
H	-2.42800	-0.74980	6.19600	N	-1.34860	-1.47040	1.93700
C	-1.64360	-2.69440	5.73930	C	-2.53490	-3.54770	2.16480
H	-2.50310	-3.24150	6.10650	C	-3.92000	-5.13450	3.30780
N	-1.67990	2.86280	5.46070	H	-4.01100	-5.98280	3.97480
N	-1.70720	1.47040	5.41960	C	-2.66910	-4.63380	3.00830
C	-6.79290	5.10170	6.57300	H	-1.78800	-5.09520	3.43680
H	-7.09560	5.80810	5.80990	C	-1.24500	1.29520	2.27060
H	-7.50410	4.28580	6.61130	H	-2.06930	0.74980	2.71340
C	-2.89360	3.54770	5.64740	C	-1.28490	2.69440	2.25670
C	-4.02500	2.96230	5.09290	H	-2.14450	3.24150	2.62390
H	-3.92910	2.11290	4.42790	O	-0.17930	-4.84400	1.74130
C	-5.27020	3.46970	5.39450	N	0.96260	-2.86280	1.50450
H	-6.14800	3.00800	4.95940	N	0.98990	-1.47040	1.54560
C	-5.42680	4.56040	6.24500	C	-0.17930	-0.89960	1.74130
C	-4.27870	5.13450	6.79030	C	-0.17930	-3.61550	1.74130
C	-3.02780	4.63380	6.49080	C	2.17630	-3.54770	1.31780
H	-2.14670	5.09520	6.91940	C	3.30770	-2.96230	1.87220
H	-6.76350	5.59880	7.53480	H	3.21180	-2.11290	2.53730
H	-4.36960	5.98280	7.45740	C	-0.17930	0.59450	1.74130
C	-0.53800	-0.59450	5.22390	C	0.88630	1.29520	1.21190
C	0.52760	-1.29520	4.69450	C	0.92630	2.69440	1.22590
H	1.35200	-0.74980	4.25170	H	1.78580	3.24150	0.85870
C	0.56760	-2.69440	4.70840	C	-0.17930	3.35100	1.74130
H	1.42710	-3.24150	4.34120	H	-0.17930	4.43400	1.74130
C	-0.53800	-3.35100	5.22390	C	-3.66630	-2.96230	1.61030
H	-0.53800	-4.43400	5.22390	C	-5.06810	-4.56040	2.76240
O	-0.53800	4.84400	5.22390	C	2.31040	-4.63380	0.47430
N	0.60400	2.86280	4.98710	C	4.55290	-3.46970	1.57060
N	0.63130	1.47040	5.02810	H	1.71070	0.74980	0.76920
C	-0.53800	0.89960	5.22390	H	-3.57050	-2.11290	0.94530
C	-0.53800	3.61550	5.22390	C	-4.91150	-3.46970	1.91190
C	5.71700	5.10170	3.87470	C	-6.43430	-5.10170	3.09040
H	6.01960	5.80810	4.63780	C	3.56130	-5.13450	0.17480
H	5.68750	5.59880	2.91300	H	1.42940	-5.09520	0.04580
H	6.42810	4.28580	3.83640	H	5.43070	-3.00800	2.00580
C	1.81760	3.54770	4.80040	C	4.70940	-4.56040	0.72020
C	2.94900	2.96230	5.35480	H	-5.78940	-3.00800	1.47680
H	2.85310	2.11290	6.01980	H	-6.73690	-5.80810	2.32730
C	4.19420	3.46970	5.05320	H	-6.40480	-5.59880	4.05220
H	5.07200	3.00800	5.48830	H	-7.14540	-4.28580	3.12870
C	4.35080	4.56040	4.20280	H	3.65230	-5.98280	-0.49220
C	3.20270	5.13450	3.65740	C	6.07560	-5.10170	0.39210
H	3.29360	5.98280	2.99040	H	6.37820	-5.80810	1.15520
C	1.95180	4.63380	3.95690	H	6.04620	-5.59880	-0.56960
H	1.07070	5.09520	3.52830	H	6.78680	-4.28580	0.35380

Table S25: Cartesian coordinates of J_4 in pTolOV at 2.73 GPa.

	Molecule 1				Molecule 2		
	x	y	z		x	y	z
C	-6.43430	6.20030	3.09040	C	-1.60360	-1.29520	5.75320
H	-6.73690	5.49390	2.32730	H	-2.42800	-0.74980	6.19600
H	-6.40480	5.70320	4.05220	C	-1.64360	-2.69440	5.73930
H	-7.14540	7.01620	3.12870	H	-2.50310	-3.24150	6.10650
C	-5.06810	6.74160	2.76240	N	-1.67990	2.86280	5.46070
C	-3.92000	6.16750	3.30780	N	-1.70720	1.47040	5.41960
H	-4.01100	5.31920	3.97480	C	-6.79290	5.10170	6.57300
C	-2.66910	6.66820	3.00830	H	-7.09560	5.80810	5.80990
H	-1.78800	6.20680	3.43680	H	-7.50410	4.28580	6.61130
H	6.04620	5.70320	-0.56960	C	-2.89360	3.54770	5.64740
H	3.65230	5.31920	-0.49220	C	-4.02500	2.96230	5.09290
O	-0.17930	6.45800	1.74130	H	-3.92910	2.11290	4.42790
N	0.96260	8.43920	1.50450	C	-5.27020	3.46970	5.39450
C	-0.17930	7.68650	1.74130	H	-6.14800	3.00800	4.95940
C	6.07560	6.20030	0.39210	C	-5.42680	4.56040	6.24500
H	6.37820	5.49390	1.15520	C	-4.27870	5.13450	6.79030
H	6.78680	7.01620	0.35380	C	-3.02780	4.63380	6.49080
C	2.17630	7.75430	1.31780	H	-2.14670	5.09520	6.91940
C	3.30770	8.33970	1.87220	H	-6.76350	5.59880	7.53480
H	3.21180	9.18910	2.53730	H	-4.36960	5.98280	7.45740
C	4.55290	7.83230	1.57060	C	-0.53800	-0.59450	5.22390
H	5.43070	8.29400	2.00580	C	0.52760	-1.29520	4.69450
C	4.70940	6.74160	0.72020	H	1.35200	-0.74980	4.25170
C	3.56130	6.16750	0.17480	C	0.56760	-2.69440	4.70840
C	2.31040	6.66820	0.47430	H	1.42710	-3.24150	4.34120
H	1.42940	6.20680	0.04580	C	-0.53800	-3.35100	5.22390
C	-4.91150	7.83230	1.91190	H	-0.53800	-4.43400	5.22390
C	-2.53490	7.75430	2.16480	O	-0.53800	4.84400	5.22390
N	0.98990	9.83160	1.54560	N	0.60400	2.86280	4.98710
N	-1.32130	8.43920	1.97810	N	0.63130	1.47040	5.02810
C	-3.66630	8.33970	1.61030	C	-0.53800	0.89960	5.22390
H	-5.78940	8.29400	1.47680	C	-0.53800	3.61550	5.22390
C	-0.17930	10.40240	1.74130	C	5.71700	5.10170	3.87470
N	-1.34860	9.83160	1.93700	H	6.01960	5.80810	4.63780
H	-3.57050	9.18910	0.94530	H	5.68750	5.59880	2.91300
C	-0.17930	11.89650	1.74130	H	6.42810	4.28580	3.83640
C	0.88630	12.59720	1.21190	C	1.81760	3.54770	4.80040
C	-1.24500	12.59720	2.27060	C	2.94900	2.96230	5.35480
H	1.71070	12.05180	0.76920	H	2.85310	2.11290	6.01980
C	0.92630	13.99640	1.22590	C	4.19420	3.46970	5.05320
H	-2.06930	12.05180	2.71340	H	5.07200	3.00800	5.48830
C	-1.28490	13.99640	2.25670	C	4.35080	4.56040	4.20280
H	1.78580	14.54350	0.85870	C	3.20270	5.13450	3.65740
C	-0.17930	14.65300	1.74130	H	3.29360	5.98280	2.99040
H	-2.14450	14.54350	2.62390	C	1.95180	4.63380	3.95690
H	-0.17930	15.73600	1.74130	H	1.07070	5.09520	3.52830

Table S26: Cartesian coordinates of J_5 in pTolOV at 2.73 GPa.

	Molecule 1				Molecule 2		
	x	y	z		x	y	z
C	-1.60360	-1.29520	5.75320	C	-1.64360	8.60760	5.73930
H	-2.42800	-0.74980	6.19600	H	-2.50310	8.06050	6.10650
C	-1.64360	-2.69440	5.73930	C	0.56760	8.60760	4.70840
H	-2.50310	-3.24150	6.10650	H	1.42710	8.06050	4.34120
N	-1.67990	2.86280	5.46070	C	-0.53800	7.95100	5.22390
N	-1.70720	1.47040	5.41960	H	-0.53800	6.86800	5.22390
C	-6.79290	5.10170	6.57300	C	-1.60360	10.00680	5.75320
H	-7.09560	5.80810	5.80990	C	0.52760	10.00680	4.69450
H	-7.50410	4.28580	6.61130	C	-0.53800	10.70750	5.22390
C	-2.89360	3.54770	5.64740	H	-2.42800	10.55220	6.19600
C	-4.02500	2.96230	5.09290	H	1.35200	10.55220	4.25170
H	-3.92910	2.11290	4.42790	C	-0.53800	12.20160	5.22390
C	-5.27020	3.46970	5.39450	N	0.63130	12.77240	5.02810
H	-6.14800	3.00800	4.95940	N	-1.70720	12.77240	5.41960
C	-5.42680	4.56040	6.24500	N	0.60400	14.16480	4.98710
C	-4.27870	5.13450	6.79030	N	-1.67990	14.16480	5.46070
C	-3.02780	4.63380	6.49080	C	-0.53800	14.91750	5.22390
H	-2.14670	5.09520	6.91940	C	1.81760	14.84970	4.80040
H	-6.76350	5.59880	7.53480	C	-2.89360	14.84970	5.64740
H	-4.36960	5.98280	7.45740	O	-0.53800	16.14600	5.22390
C	-0.53800	-0.59450	5.22390	C	2.94900	14.26430	5.35480
C	0.52760	-1.29520	4.69450	C	1.95180	15.93580	3.95690
H	1.35200	-0.74980	4.25170	C	-4.02500	14.26430	5.09290
C	0.56760	-2.69440	4.70840	C	-3.02780	15.93580	6.49080
H	1.42710	-3.24150	4.34120	H	2.85310	13.41490	6.01980
C	-0.53800	-3.35100	5.22390	C	4.19420	14.77170	5.05320
H	-0.53800	-4.43400	5.22390	C	3.20270	16.43650	3.65740
O	-0.53800	4.84400	5.22390	H	1.07070	16.39720	3.52830
N	0.60400	2.86280	4.98710	H	-3.92910	13.41490	4.42790
N	0.63130	1.47040	5.02810	C	-5.27020	14.77170	5.39450
C	-0.53800	0.89960	5.22390	C	-4.27870	16.43650	6.79030
C	-0.53800	3.61550	5.22390	H	-2.14670	16.39720	6.91940
C	5.71700	5.10170	3.87470	H	5.07200	14.31000	5.48830
H	6.01960	5.80810	4.63780	C	4.35080	15.86240	4.20280
H	5.68750	5.59880	2.91300	H	3.29360	17.28480	2.99040
H	6.42810	4.28580	3.83640	H	-6.14800	14.31000	4.95940
C	1.81760	3.54770	4.80040	C	-5.42680	15.86240	6.24500
C	2.94900	2.96230	5.35480	H	-4.36960	17.28480	7.45740
H	2.85310	2.11290	6.01980	C	5.71700	16.40370	3.87470
C	4.19420	3.46970	5.05320	C	-6.79290	16.40370	6.57300
H	5.07200	3.00800	5.48830	H	6.01960	17.11010	4.63780
C	4.35080	4.56040	4.20280	H	5.68750	16.90080	2.91300
C	3.20270	5.13450	3.65740	H	6.42810	15.58780	3.83640
H	3.29360	5.98280	2.99040	H	-7.09560	17.11010	5.80990
C	1.95180	4.63380	3.95690	H	-6.76350	16.90080	7.53480
H	1.07070	5.09520	3.52830	H	-7.50410	15.58780	6.61130

Table S27: Cartesian coordinates of J_6 in pTolOV at 2.73 GPa.

	Molecule 1				Molecule 2		
	x	y	z		x	y	z
N	-2.03860	8.43920	8.94330	C	-1.60360	-1.29520	5.75320
C	-7.15160	6.20030	10.05560	H	-2.42800	-0.74980	6.19600
H	-7.45420	5.49390	9.29250	C	-1.64360	-2.69440	5.73930
H	-7.12210	5.70320	11.01740	H	-2.50310	-3.24150	6.10650
H	-7.86280	7.01620	10.09390	N	-1.67990	2.86280	5.46070
C	-3.25220	7.75430	9.12990	N	-1.70720	1.47040	5.41960
C	-4.38370	8.33970	8.57550	C	-6.79290	5.10170	6.57300
H	-4.28780	9.18910	7.91050	H	-7.09560	5.80810	5.80990
C	-5.62890	7.83230	8.87710	H	-7.50410	4.28580	6.61130
H	-6.50670	8.29400	8.44200	C	-2.89360	3.54770	5.64740
C	-5.78540	6.74160	9.72750	C	-4.02500	2.96230	5.09290
C	-4.63730	6.16750	10.27290	H	-3.92910	2.11290	4.42790
H	-4.72830	5.31920	10.94000	C	-5.27020	3.46970	5.39450
C	-3.38640	6.66820	9.97340	H	-6.14800	3.00800	4.95940
H	-2.50540	6.20680	10.40200	C	-5.42680	4.56040	6.24500
H	5.32880	5.70320	6.39550	C	-4.27870	5.13450	6.79030
H	2.93500	5.31920	6.47290	C	-3.02780	4.63380	6.49080
O	-0.89670	6.45800	8.70640	H	-2.14670	5.09520	6.91940
C	-0.89670	7.68650	8.70640	H	-6.76350	5.59880	7.53480
C	5.35830	6.20030	7.35730	H	-4.36960	5.98280	7.45740
H	5.66090	5.49390	8.12040	C	-0.53800	-0.59450	5.22390
H	6.06940	7.01620	7.31900	C	0.52760	-1.29520	4.69450
C	3.99210	6.74160	7.68540	H	1.35200	-0.74980	4.25170
C	2.84400	6.16750	7.14000	C	0.56760	-2.69440	4.70840
C	1.59310	6.66820	7.43950	H	1.42710	-3.24150	4.34120
H	0.71210	6.20680	7.01090	C	-0.53800	-3.35100	5.22390
N	-2.06590	9.83160	8.90220	H	-0.53800	-4.43400	5.22390
N	0.24530	8.43920	8.46960	O	-0.53800	4.84400	5.22390
C	3.83560	7.83230	8.53580	N	0.60400	2.86280	4.98710
C	1.45890	7.75430	8.28300	N	0.63130	1.47040	5.02810
C	-0.89670	10.40240	8.70640	C	-0.53800	0.89960	5.22390
N	0.27260	9.83160	8.51070	C	-0.53800	3.61550	5.22390
C	2.59040	8.33970	8.83740	C	5.71700	5.10170	3.87470
H	4.71340	8.29400	8.97090	H	6.01960	5.80810	4.63780
C	-0.89670	11.89650	8.70640	H	5.68750	5.59880	2.91300
H	2.49450	9.18910	9.50240	H	6.42810	4.28580	3.83640
C	0.16900	12.59720	8.17710	C	1.81760	3.54770	4.80040
C	-1.96230	12.59720	9.23580	C	2.94900	2.96230	5.35480
H	0.99330	12.05180	7.73430	H	2.85310	2.11290	6.01980
C	0.20890	13.99640	8.19100	C	4.19420	3.46970	5.05320
H	-2.78660	12.05180	9.67860	H	5.07200	3.00800	5.48830
C	-2.00220	13.99640	9.22190	C	4.35080	4.56040	4.20280
H	1.06850	14.54350	7.82380	C	3.20270	5.13450	3.65740
C	-0.89670	14.65300	8.70640	H	3.29360	5.98280	2.99040
H	-2.86180	14.54350	9.58910	C	1.95180	4.63380	3.95690
H	-0.89670	15.73600	8.70640	H	1.07070	5.09520	3.52830

Table S28: Cartesian coordinates of J_7 in pTolOV at 2.73 GPa.

	Molecule 1				Molecule 2		
	x	y	z		x	y	z
H	-6.92000	-0.33180	6.47290	C	-1.60360	-1.29520	5.75320
H	-4.19410	-0.15710	8.12040	H	-2.42800	-0.74980	6.19600
H	-4.52620	0.05220	6.39550	C	-1.64360	-2.69440	5.73930
N	-9.60970	2.78820	8.46960	H	-2.50310	-3.24150	6.10650
N	-9.58240	4.18060	8.51070	N	-1.67990	2.86280	5.46070
C	-9.68600	6.94620	8.17710	N	-1.70720	1.47040	5.41960
H	-8.86170	6.40080	7.73430	C	-6.79290	5.10170	6.57300
C	-4.49670	0.54930	7.35730	H	-7.09560	5.80810	5.80990
H	-3.78560	1.36520	7.31900	H	-7.50410	4.28580	6.61130
C	-8.39610	2.10330	8.28300	C	-2.89360	3.54770	5.64740
C	-7.26460	2.68870	8.83740	C	-4.02500	2.96230	5.09290
H	-7.36050	3.53810	9.50240	H	-3.92910	2.11290	4.42790
C	-6.01940	2.18130	8.53580	C	-5.27020	3.46970	5.39450
H	-5.14160	2.64300	8.97090	H	-6.14800	3.00800	4.95940
C	-5.86290	1.09060	7.68540	C	-5.42680	4.56040	6.24500
C	-7.01100	0.51650	7.14000	C	-4.27870	5.13450	6.79030
C	-8.26190	1.01720	7.43950	C	-3.02780	4.63380	6.49080
C	-10.75170	2.03550	8.70640	H	-2.14670	5.09520	6.91940
C	-10.75170	4.75140	8.70640	H	-6.76350	5.59880	7.53480
C	-10.75170	6.24550	8.70640	H	-4.36960	5.98280	7.45740
C	-9.64610	8.34540	8.19100	C	-0.53800	-0.59450	5.22390
H	-9.14290	0.55580	7.01090	C	0.52760	-1.29520	4.69450
O	-10.75170	0.80700	8.70640	H	1.35200	-0.74980	4.25170
N	-11.89360	2.78820	8.94330	C	0.56760	-2.69440	4.70840
N	-11.92090	4.18060	8.90220	H	1.42710	-3.24150	4.34120
C	-11.81730	6.94620	9.23580	C	-0.53800	-3.35100	5.22390
H	-8.78650	8.89250	7.82380	H	-0.53800	-4.43400	5.22390
C	-10.75170	9.00200	8.70640	O	-0.53800	4.84400	5.22390
C	-13.10720	2.10330	9.12990	N	0.60400	2.86280	4.98710
H	-12.64160	6.40080	9.67860	N	0.63130	1.47040	5.02810
C	-11.85720	8.34540	9.22190	C	-0.53800	0.89960	5.22390
H	-10.75170	10.08500	8.70640	C	-0.53800	3.61550	5.22390
C	-14.23870	2.68870	8.57550	C	5.71700	5.10170	3.87470
C	-13.24140	1.01720	9.97340	H	6.01960	5.80810	4.63780
H	-12.71680	8.89250	9.58910	H	5.68750	5.59880	2.91300
H	-14.14280	3.53810	7.91050	H	6.42810	4.28580	3.83640
C	-15.48390	2.18130	8.87710	C	1.81760	3.54770	4.80040
C	-14.49230	0.51650	10.27290	C	2.94900	2.96230	5.35480
H	-12.36040	0.55580	10.40200	H	2.85310	2.11290	6.01980
H	-16.36170	2.64300	8.44200	C	4.19420	3.46970	5.05320
C	-15.64040	1.09060	9.72750	H	5.07200	3.00800	5.48830
H	-14.58330	-0.33180	10.94000	C	4.35080	4.56040	4.20280
C	-17.00660	0.54930	10.05560	C	3.20270	5.13450	3.65740
H	-17.30920	-0.15710	9.29250	H	3.29360	5.98280	2.99040
H	-16.97710	0.05220	11.01740	C	1.95180	4.63380	3.95690
H	-17.71780	1.36520	10.09390	H	1.07070	5.09520	3.52830

Table S29: Cartesian coordinates of J_8 in pTolOV at 2.73 GPa.

	Molecule 1				Molecule 2		
	x	y	z		x	y	z
N	-9.25100	8.51380	4.98710	C	-1.60360	-1.29520	5.75320
N	-9.22370	7.12140	5.02810	H	-2.42800	-0.74980	6.19600
C	-10.39300	6.55060	5.22390	C	-1.64360	-2.69440	5.73930
C	-10.39300	5.05650	5.22390	H	-2.50310	-3.24150	6.10650
C	-9.32740	4.35580	4.69450	N	-1.67990	2.86280	5.46070
H	-8.50300	4.90120	4.25170	N	-1.70720	1.47040	5.41960
C	-9.28740	2.95660	4.70840	C	-6.79290	5.10170	6.57300
H	-8.42790	2.40950	4.34120	H	-7.09560	5.80810	5.80990
C	-10.39300	2.30000	5.22390	H	-7.50410	4.28580	6.61130
C	-8.03740	9.19870	4.80040	C	-2.89360	3.54770	5.64740
C	-6.90600	8.61330	5.35480	C	-4.02500	2.96230	5.09290
H	-7.00190	7.76390	6.01980	H	-3.92910	2.11290	4.42790
C	-5.66080	9.12070	5.05320	C	-5.27020	3.46970	5.39450
H	-4.78300	8.65900	5.48830	H	-6.14800	3.00800	4.95940
C	-10.39300	9.26650	5.22390	C	-5.42680	4.56040	6.24500
N	-11.56220	7.12140	5.41960	C	-4.27870	5.13450	6.79030
C	-11.45860	4.35580	5.75320	C	-3.02780	4.63380	6.49080
C	-11.49860	2.95660	5.73930	H	-2.14670	5.09520	6.91940
H	-10.39300	1.21700	5.22390	H	-6.76350	5.59880	7.53480
C	-7.90320	10.28480	3.95690	H	-4.36960	5.98280	7.45740
C	-5.50420	10.21140	4.20280	C	-0.53800	-0.59450	5.22390
O	-10.39300	10.49500	5.22390	C	0.52760	-1.29520	4.69450
N	-11.53490	8.51380	5.46070	H	1.35200	-0.74980	4.25170
H	-12.28300	4.90120	6.19600	C	0.56760	-2.69440	4.70840
H	-12.35810	2.40950	6.10650	H	1.42710	-3.24150	4.34120
C	-6.65230	10.78550	3.65740	C	-0.53800	-3.35100	5.22390
H	-8.78430	10.74620	3.52830	H	-0.53800	-4.43400	5.22390
C	-4.13800	10.75270	3.87470	O	-0.53800	4.84400	5.22390
C	-12.74860	9.19870	5.64740	N	0.60400	2.86280	4.98710
H	-6.56140	11.63380	2.99040	N	0.63130	1.47040	5.02810
H	-3.83540	11.45910	4.63780	C	-0.53800	0.89960	5.22390
H	-4.16750	11.24980	2.91300	C	-0.53800	3.61550	5.22390
H	-3.42690	9.93680	3.83640	C	5.71700	5.10170	3.87470
C	-13.88000	8.61330	5.09290	H	6.01960	5.80810	4.63780
C	-12.88280	10.28480	6.49080	H	5.68750	5.59880	2.91300
H	-13.78410	7.76390	4.42790	H	6.42810	4.28580	3.83640
C	-15.12520	9.12070	5.39450	C	1.81760	3.54770	4.80040
C	-14.13370	10.78550	6.79030	C	2.94900	2.96230	5.35480
H	-12.00170	10.74620	6.91940	H	2.85310	2.11290	6.01980
H	-16.00300	8.65900	4.95940	C	4.19420	3.46970	5.05320
C	-15.28180	10.21140	6.24500	H	5.07200	3.00800	5.48830
H	-14.22460	11.63380	7.45740	C	4.35080	4.56040	4.20280
C	-16.64790	10.75270	6.57300	C	3.20270	5.13450	3.65740
H	-16.95060	11.45910	5.80990	H	3.29360	5.98280	2.99040
H	-16.61850	11.24980	7.53480	C	1.95180	4.63380	3.95690
H	-17.35910	9.93680	6.61130	H	1.07070	5.09520	3.52830

Table S30: Cartesian coordinates of J_9 in pTolOV at 2.73 GPa.

	Molecule 1				Molecule 2		
	x	y	z		x	y	z
C	-1.60360	-1.29520	5.75320	C	-6.54730	2.68870	1.87220
H	-2.42800	-0.74980	6.19600	H	-6.64320	3.53810	2.53730
C	-1.64360	-2.69440	5.73930	C	-5.30210	2.18130	1.57060
H	-2.50310	-3.24150	6.10650	H	-4.42430	2.64300	2.00580
N	-1.67990	2.86280	5.46070	C	-7.67870	2.10330	1.31780
N	-1.70720	1.47040	5.41960	C	-5.14560	1.09060	0.72020
C	-6.79290	5.10170	6.57300	N	-8.89240	2.78820	1.50450
H	-7.09560	5.80810	5.80990	C	-7.54460	1.01720	0.47430
H	-7.50410	4.28580	6.61130	C	-3.77940	0.54930	0.39210
C	-2.89360	3.54770	5.64740	C	-6.29370	0.51650	0.17480
C	-4.02500	2.96230	5.09290	N	-8.86510	4.18060	1.54560
H	-3.92910	2.11290	4.42790	C	-10.03430	2.03550	1.74130
C	-5.27020	3.46970	5.39450	H	-8.42560	0.55580	0.04580
H	-6.14800	3.00800	4.95940	H	-3.47680	-0.15710	1.15520
C	-5.42680	4.56040	6.24500	H	-3.80880	0.05220	-0.56960
C	-4.27870	5.13450	6.79030	H	-3.06820	1.36520	0.35380
C	-3.02780	4.63380	6.49080	H	-6.20270	-0.33180	-0.49220
H	-2.14670	5.09520	6.91940	C	-10.03430	4.75140	1.74130
H	-6.76350	5.59880	7.53480	O	-10.03430	0.80700	1.74130
H	-4.36960	5.98280	7.45740	N	-11.17630	2.78820	1.97810
C	-0.53800	-0.59450	5.22390	N	-11.20360	4.18060	1.93700
C	0.52760	-1.29520	4.69450	C	-10.03430	6.24550	1.74130
H	1.35200	-0.74980	4.25170	C	-12.38990	2.10330	2.16480
C	0.56760	-2.69440	4.70840	C	-8.96870	6.94620	1.21190
H	1.42710	-3.24150	4.34120	C	-11.10000	6.94620	2.27060
C	-0.53800	-3.35100	5.22390	C	-13.52130	2.68870	1.61030
H	-0.53800	-4.43400	5.22390	C	-12.52410	1.01720	3.00830
O	-0.53800	4.84400	5.22390	H	-8.14430	6.40080	0.76920
N	0.60400	2.86280	4.98710	C	-8.92870	8.34540	1.22590
N	0.63130	1.47040	5.02810	H	-11.92430	6.40080	2.71340
C	-0.53800	0.89960	5.22390	C	-11.13990	8.34540	2.25670
C	-0.53800	3.61550	5.22390	H	-13.42550	3.53810	0.94530
C	5.71700	5.10170	3.87470	C	-14.76650	2.18130	1.91190
H	6.01960	5.80810	4.63780	C	-13.77500	0.51650	3.30780
H	5.68750	5.59880	2.91300	H	-11.64300	0.55580	3.43680
H	6.42810	4.28580	3.83640	H	-8.06920	8.89250	0.85870
C	1.81760	3.54770	4.80040	C	-10.03430	9.00200	1.74130
C	2.94900	2.96230	5.35480	H	-11.99950	8.89250	2.62390
H	2.85310	2.11290	6.01980	H	-15.64440	2.64300	1.47680
C	4.19420	3.46970	5.05320	C	-14.92310	1.09060	2.76240
H	5.07200	3.00800	5.48830	H	-13.86600	-0.33180	3.97480
C	4.35080	4.56040	4.20280	H	-10.03430	10.08500	1.74130
C	3.20270	5.13450	3.65740	C	-16.28930	0.54930	3.09040
H	3.29360	5.98280	2.99040	H	-16.59190	-0.15710	2.32730
C	1.95180	4.63380	3.95690	H	-16.25980	0.05220	4.05220
H	1.07070	5.09520	3.52830	H	-17.00040	1.36520	3.12870

Table S31: Cartesian coordinates of J_{10} in pTolOV at 2.73 GPa.

	Molecule 1				Molecule 2		
	x	y	z		x	y	z
C	5.47130	2.68870	8.57550	C	-1.60360	-1.29520	5.75320
H	5.56720	3.53810	7.91050	H	-2.42800	-0.74980	6.19600
C	4.22610	2.18130	8.87710	C	-1.64360	-2.69440	5.73930
H	3.34830	2.64300	8.44200	H	-2.50310	-3.24150	6.10650
C	6.60280	2.10330	9.12990	N	-1.67990	2.86280	5.46070
C	4.06960	1.09060	9.72750	N	-1.70720	1.47040	5.41960
N	7.81640	2.78820	8.94330	C	-6.79290	5.10170	6.57300
C	6.46860	1.01720	9.97340	H	-7.09560	5.80810	5.80990
C	2.70340	0.54930	10.05560	H	-7.50410	4.28580	6.61130
C	5.21770	0.51650	10.27290	C	-2.89360	3.54770	5.64740
N	7.78910	4.18060	8.90220	C	-4.02500	2.96230	5.09290
C	8.95830	2.03550	8.70640	H	-3.92910	2.11290	4.42790
H	7.34960	0.55580	10.40200	C	-5.27020	3.46970	5.39450
H	2.40080	-0.15710	9.29250	H	-6.14800	3.00800	4.95940
H	2.73290	0.05220	11.01740	C	-5.42680	4.56040	6.24500
H	1.99220	1.36520	10.09390	C	-4.27870	5.13450	6.79030
H	5.12670	-0.33180	10.94000	C	-3.02780	4.63380	6.49080
C	8.95830	4.75140	8.70640	H	-2.14670	5.09520	6.91940
O	8.95830	0.80700	8.70640	H	-6.76350	5.59880	7.53480
N	10.10030	2.78820	8.46960	H	-4.36960	5.98280	7.45740
N	10.12760	4.18060	8.51070	C	-0.53800	-0.59450	5.22390
C	8.95830	6.24550	8.70640	C	0.52760	-1.29520	4.69450
C	11.31390	2.10330	8.28300	H	1.35200	-0.74980	4.25170
C	10.02400	6.94620	8.17710	C	0.56760	-2.69440	4.70840
C	7.89270	6.94620	9.23580	H	1.42710	-3.24150	4.34120
C	12.44540	2.68870	8.83740	C	-0.53800	-3.35100	5.22390
C	11.44810	1.01720	7.43950	H	-0.53800	-4.43400	5.22390
H	10.84830	6.40080	7.73430	O	-0.53800	4.84400	5.22390
C	10.06390	8.34540	8.19100	N	0.60400	2.86280	4.98710
H	7.06840	6.40080	9.67860	N	0.63130	1.47040	5.02810
C	7.85280	8.34540	9.22190	C	-0.53800	0.89960	5.22390
H	12.34950	3.53810	9.50240	C	-0.53800	3.61550	5.22390
C	13.69060	2.18130	8.53580	C	5.71700	5.10170	3.87470
C	12.69900	0.51650	7.14000	H	6.01960	5.80810	4.63780
H	10.56710	0.55580	7.01090	H	5.68750	5.59880	2.91300
H	10.92350	8.89250	7.82380	H	6.42810	4.28580	3.83640
C	8.95830	9.00200	8.70640	C	1.81760	3.54770	4.80040
H	6.99320	8.89250	9.58910	C	2.94900	2.96230	5.35480
H	14.56840	2.64300	8.97090	H	2.85310	2.11290	6.01980
C	13.84710	1.09060	7.68540	C	4.19420	3.46970	5.05320
H	12.79000	-0.33180	6.47290	H	5.07200	3.00800	5.48830
H	8.95830	10.08500	8.70640	C	4.35080	4.56040	4.20280
C	15.21330	0.54930	7.35730	C	3.20270	5.13450	3.65740
H	15.51590	-0.15710	8.12040	H	3.29360	5.98280	2.99040
H	15.18380	0.05220	6.39550	C	1.95180	4.63380	3.95690
H	15.92440	1.36520	7.31900	H	1.07070	5.09520	3.52830

Table S32: Cartesian coordinates of J_{11} in pTolOV at 2.73 GPa.

	Molecule 1				Molecule 2		
	x	y	z		x	y	z
N	8.17510	8.51380	5.46070	C	-1.60360	-1.29520	5.75320
N	8.14780	7.12140	5.41960	H	-2.42800	-0.74980	6.19600
C	9.31700	6.55060	5.22390	C	-1.64360	-2.69440	5.73930
C	9.31700	5.05650	5.22390	H	-2.50310	-3.24150	6.10650
C	8.25140	4.35580	5.75320	N	-1.67990	2.86280	5.46070
H	7.42700	4.90120	6.19600	N	-1.70720	1.47040	5.41960
C	8.21140	2.95660	5.73930	C	-6.79290	5.10170	6.57300
H	7.35190	2.40950	6.10650	H	-7.09560	5.80810	5.80990
C	9.31700	2.30000	5.22390	H	-7.50410	4.28580	6.61130
C	6.96140	9.19870	5.64740	C	-2.89360	3.54770	5.64740
C	5.83000	8.61330	5.09290	C	-4.02500	2.96230	5.09290
H	5.92590	7.76390	4.42790	H	-3.92910	2.11290	4.42790
C	4.58480	9.12070	5.39450	C	-5.27020	3.46970	5.39450
H	3.70700	8.65900	4.95940	H	-6.14800	3.00800	4.95940
C	9.31700	9.26650	5.22390	C	-5.42680	4.56040	6.24500
N	10.48630	7.12140	5.02810	C	-4.27870	5.13450	6.79030
C	10.38260	4.35580	4.69450	C	-3.02780	4.63380	6.49080
C	10.42260	2.95660	4.70840	H	-2.14670	5.09520	6.91940
H	9.31700	1.21700	5.22390	H	-6.76350	5.59880	7.53480
C	6.82720	10.28480	6.49080	H	-4.36960	5.98280	7.45740
C	4.42820	10.21140	6.24500	C	-0.53800	-0.59450	5.22390
O	9.31700	10.49500	5.22390	C	0.52760	-1.29520	4.69450
N	10.45900	8.51380	4.98710	H	1.35200	-0.74980	4.25170
H	11.20700	4.90120	4.25170	C	0.56760	-2.69440	4.70840
H	11.28210	2.40950	4.34120	H	1.42710	-3.24150	4.34120
C	5.57630	10.78550	6.79030	C	-0.53800	-3.35100	5.22390
H	7.70830	10.74620	6.91940	H	-0.53800	-4.43400	5.22390
C	3.06210	10.75270	6.57300	O	-0.53800	4.84400	5.22390
C	11.67260	9.19870	4.80040	N	0.60400	2.86280	4.98710
H	5.48540	11.63380	7.45740	N	0.63130	1.47040	5.02810
H	2.75940	11.45910	5.80990	C	-0.53800	0.89960	5.22390
H	3.09150	11.24980	7.53480	C	-0.53800	3.61550	5.22390
H	2.35090	9.93680	6.61130	C	5.71700	5.10170	3.87470
C	12.80400	8.61330	5.35480	H	6.01960	5.80810	4.63780
C	11.80680	10.28480	3.95690	H	5.68750	5.59880	2.91300
H	12.70810	7.76390	6.01980	H	6.42810	4.28580	3.83640
C	14.04920	9.12070	5.05320	C	1.81760	3.54770	4.80040
C	13.05770	10.78550	3.65740	C	2.94900	2.96230	5.35480
H	10.92570	10.74620	3.52830	H	2.85310	2.11290	6.01980
H	14.92700	8.65900	5.48830	C	4.19420	3.46970	5.05320
C	14.20580	10.21140	4.20280	H	5.07200	3.00800	5.48830
H	13.14860	11.63380	2.99040	C	4.35080	4.56040	4.20280
C	15.57200	10.75270	3.87470	C	3.20270	5.13450	3.65740
H	15.87460	11.45910	4.63780	H	3.29360	5.98280	2.99040
H	15.54250	11.24980	2.91300	C	1.95180	4.63380	3.95690
H	16.28310	9.93680	3.83640	H	1.07070	5.09520	3.52830

Table S33: Cartesian coordinates of J_{12} in pTolOV at 2.73 GPa.

	Molecule 1				Molecule 2		
	x	y	z		x	y	z
H	3.11810	-0.15710	2.32730	C	-1.60360	-1.29520	5.75320
H	5.84400	-0.33180	3.97480	H	-2.42800	-0.74980	6.19600
N	8.53370	2.78820	1.97810	C	-1.64360	-2.69440	5.73930
N	8.50640	4.18060	1.93700	H	-2.50310	-3.24150	6.10650
C	8.61000	6.94620	2.27060	N	-1.67990	2.86280	5.46070
H	7.78570	6.40080	2.71340	N	-1.70720	1.47040	5.41960
C	3.42070	0.54930	3.09040	C	-6.79290	5.10170	6.57300
H	3.45020	0.05220	4.05220	H	-7.09560	5.80810	5.80990
H	2.70960	1.36520	3.12870	H	-7.50410	4.28580	6.61130
C	7.32010	2.10330	2.16480	C	-2.89360	3.54770	5.64740
C	6.18870	2.68870	1.61030	C	-4.02500	2.96230	5.09290
H	6.28450	3.53810	0.94530	H	-3.92910	2.11290	4.42790
C	4.94350	2.18130	1.91190	C	-5.27020	3.46970	5.39450
H	4.06560	2.64300	1.47680	H	-6.14800	3.00800	4.95940
C	4.78690	1.09060	2.76240	C	-5.42680	4.56040	6.24500
C	5.93500	0.51650	3.30780	C	-4.27870	5.13450	6.79030
C	7.18590	1.01720	3.00830	C	-3.02780	4.63380	6.49080
C	9.67570	2.03550	1.74130	H	-2.14670	5.09520	6.91940
C	9.67570	4.75140	1.74130	H	-6.76350	5.59880	7.53480
C	9.67570	6.24550	1.74130	H	-4.36960	5.98280	7.45740
C	8.57010	8.34540	2.25670	C	-0.53800	-0.59450	5.22390
H	8.06700	0.55580	3.43680	C	0.52760	-1.29520	4.69450
O	9.67570	0.80700	1.74130	H	1.35200	-0.74980	4.25170
N	10.81760	2.78820	1.50450	C	0.56760	-2.69440	4.70840
N	10.84490	4.18060	1.54560	H	1.42710	-3.24150	4.34120
C	10.74130	6.94620	1.21190	C	-0.53800	-3.35100	5.22390
H	7.71050	8.89250	2.62390	H	-0.53800	-4.43400	5.22390
C	9.67570	9.00200	1.74130	O	-0.53800	4.84400	5.22390
C	12.03130	2.10330	1.31780	N	0.60400	2.86280	4.98710
H	11.56570	6.40080	0.76920	N	0.63130	1.47040	5.02810
C	10.78130	8.34540	1.22590	C	-0.53800	0.89960	5.22390
H	9.67570	10.08500	1.74130	C	-0.53800	3.61550	5.22390
C	13.16270	2.68870	1.87220	C	5.71700	5.10170	3.87470
C	12.16540	1.01720	0.47430	H	6.01960	5.80810	4.63780
H	11.64080	8.89250	0.85870	H	5.68750	5.59880	2.91300
H	13.06680	3.53810	2.53730	H	6.42810	4.28580	3.83640
C	14.40790	2.18130	1.57060	C	1.81760	3.54770	4.80040
C	13.41630	0.51650	0.17480	C	2.94900	2.96230	5.35480
H	11.28440	0.55580	0.04580	H	2.85310	2.11290	6.01980
H	15.28570	2.64300	2.00580	C	4.19420	3.46970	5.05320
C	14.56440	1.09060	0.72020	H	5.07200	3.00800	5.48830
H	13.50730	-0.33180	-0.49220	C	4.35080	4.56040	4.20280
C	15.93060	0.54930	0.39210	C	3.20270	5.13450	3.65740
H	16.23320	-0.15710	1.15520	H	3.29360	5.98280	2.99040
H	15.90120	0.05220	-0.56960	C	1.95180	4.63380	3.95690
H	16.64180	1.36520	0.35380	H	1.07070	5.09520	3.52830

Table S34: Cartesian coordinates of J_{13} in pTolOV at 2.73 GPa.

	Molecule 1				Molecule 2		
	x	y	z		x	y	z
C	-1.60360	-1.29520	5.75320	C	3.06210	-0.54930	6.57300
H	-2.42800	-0.74980	6.19600	H	2.35090	-1.36520	6.61130
C	-1.64360	-2.69440	5.73930	C	4.58480	-2.18130	5.39450
H	-2.50310	-3.24150	6.10650	H	3.70700	-2.64300	4.95940
N	-1.67990	2.86280	5.46070	C	4.42820	-1.09060	6.24500
N	-1.70720	1.47040	5.41960	C	5.57630	-0.51650	6.79030
C	-6.79290	5.10170	6.57300	H	3.09150	-0.05220	7.53480
H	-7.09560	5.80810	5.80990	H	2.75940	0.15710	5.80990
H	-7.50410	4.28580	6.61130	H	5.48540	0.33180	7.45740
C	-2.89360	3.54770	5.64740	C	5.83000	-2.68870	5.09290
C	-4.02500	2.96230	5.09290	C	6.82720	-1.01720	6.49080
H	-3.92910	2.11290	4.42790	C	6.96140	-2.10330	5.64740
C	-5.27020	3.46970	5.39450	H	5.92590	-3.53810	4.42790
H	-6.14800	3.00800	4.95940	H	7.70830	-0.55580	6.91940
C	-5.42680	4.56040	6.24500	N	8.17510	-2.78820	5.46070
C	-4.27870	5.13450	6.79030	N	8.14780	-4.18060	5.41960
C	-3.02780	4.63380	6.49080	C	9.31700	-2.03550	5.22390
H	-2.14670	5.09520	6.91940	C	9.31700	-4.75140	5.22390
H	-6.76350	5.59880	7.53480	O	9.31700	-0.80700	5.22390
H	-4.36960	5.98280	7.45740	N	10.45900	-2.78820	4.98710
C	-0.53800	-0.59450	5.22390	N	10.48630	-4.18060	5.02810
C	0.52760	-1.29520	4.69450	C	9.31700	-6.24550	5.22390
H	1.35200	-0.74980	4.25170	C	11.67260	-2.10330	4.80040
C	0.56760	-2.69440	4.70840	C	8.25140	-6.94620	5.75320
H	1.42710	-3.24150	4.34120	C	10.38260	-6.94620	4.69450
C	-0.53800	-3.35100	5.22390	C	12.80400	-2.68870	5.35480
H	-0.53800	-4.43400	5.22390	C	11.80680	-1.01720	3.95690
O	-0.53800	4.84400	5.22390	H	7.42700	-6.40080	6.19600
N	0.60400	2.86280	4.98710	C	8.21140	-8.34540	5.73930
N	0.63130	1.47040	5.02810	H	11.20700	-6.40080	4.25170
C	-0.53800	0.89960	5.22390	C	10.42260	-8.34540	4.70840
C	-0.53800	3.61550	5.22390	H	12.70810	-3.53810	6.01980
C	5.71700	5.10170	3.87470	C	14.04920	-2.18130	5.05320
H	6.01960	5.80810	4.63780	C	13.05770	-0.51650	3.65740
H	5.68750	5.59880	2.91300	H	10.92570	-0.55580	3.52830
H	6.42810	4.28580	3.83640	H	7.35190	-8.89250	6.10650
C	1.81760	3.54770	4.80040	C	9.31700	-9.00200	5.22390
C	2.94900	2.96230	5.35480	H	11.28210	-8.89250	4.34120
H	2.85310	2.11290	6.01980	H	14.92700	-2.64300	5.48830
C	4.19420	3.46970	5.05320	C	14.20580	-1.09060	4.20280
H	5.07200	3.00800	5.48830	H	13.14860	0.33180	2.99040
C	4.35080	4.56040	4.20280	H	9.31700	-10.08500	5.22390
C	3.20270	5.13450	3.65740	C	15.57200	-0.54930	3.87470
H	3.29360	5.98280	2.99040	H	15.87460	0.15710	4.63780
C	1.95180	4.63380	3.95690	H	15.54250	-0.05220	2.91300
H	1.07070	5.09520	3.52830	H	16.28310	-1.36520	3.83640

Table S35: Cartesian coordinates of J_{14} in pTolOV at 2.73 GPa.

	Molecule 1				Molecule 2		
	x	y	z		x	y	z
C	-1.60360	-1.29520	5.75320	C	-4.13800	-0.54930	3.87470
H	-2.42800	-0.74980	6.19600	H	-4.16750	-0.05220	2.91300
C	-1.64360	-2.69440	5.73930	H	-3.42690	-1.36520	3.83640
H	-2.50310	-3.24150	6.10650	C	-5.66080	-2.18130	5.05320
N	-1.67990	2.86280	5.46070	H	-4.78300	-2.64300	5.48830
N	-1.70720	1.47040	5.41960	C	-5.50420	-1.09060	4.20280
C	-6.79290	5.10170	6.57300	C	-6.65230	-0.51650	3.65740
H	-7.09560	5.80810	5.80990	H	-3.83540	0.15710	4.63780
H	-7.50410	4.28580	6.61130	H	-6.56140	0.33180	2.99040
C	-2.89360	3.54770	5.64740	C	-6.90600	-2.68870	5.35480
C	-4.02500	2.96230	5.09290	C	-7.90320	-1.01720	3.95690
H	-3.92910	2.11290	4.42790	C	-8.03740	-2.10330	4.80040
C	-5.27020	3.46970	5.39450	H	-7.00190	-3.53810	6.01980
H	-6.14800	3.00800	4.95940	H	-8.78430	-0.55580	3.52830
C	-5.42680	4.56040	6.24500	N	-9.25100	-2.78820	4.98710
C	-4.27870	5.13450	6.79030	N	-9.22370	-4.18060	5.02810
C	-3.02780	4.63380	6.49080	C	-10.39300	-2.03550	5.22390
H	-2.14670	5.09520	6.91940	C	-10.39300	-4.75140	5.22390
H	-6.76350	5.59880	7.53480	O	-10.39300	-0.80700	5.22390
H	-4.36960	5.98280	7.45740	N	-11.53490	-2.78820	5.46070
C	-0.53800	-0.59450	5.22390	N	-11.56220	-4.18060	5.41960
C	0.52760	-1.29520	4.69450	C	-10.39300	-6.24550	5.22390
H	1.35200	-0.74980	4.25170	C	-12.74860	-2.10330	5.64740
C	0.56760	-2.69440	4.70840	C	-11.45860	-6.94620	5.75320
H	1.42710	-3.24150	4.34120	C	-9.32740	-6.94620	4.69450
C	-0.53800	-3.35100	5.22390	C	-13.88000	-2.68870	5.09290
H	-0.53800	-4.43400	5.22390	C	-12.88280	-1.01720	6.49080
O	-0.53800	4.84400	5.22390	H	-12.28300	-6.40080	6.19600
N	0.60400	2.86280	4.98710	C	-11.49860	-8.34540	5.73930
N	0.63130	1.47040	5.02810	H	-8.50300	-6.40080	4.25170
C	-0.53800	0.89960	5.22390	C	-9.28740	-8.34540	4.70840
C	-0.53800	3.61550	5.22390	H	-13.78410	-3.53810	4.42790
C	5.71700	5.10170	3.87470	C	-15.12520	-2.18130	5.39450
H	6.01960	5.80810	4.63780	C	-14.13370	-0.51650	6.79030
H	5.68750	5.59880	2.91300	H	-12.00170	-0.55580	6.91940
H	6.42810	4.28580	3.83640	H	-12.35810	-8.89250	6.10650
C	1.81760	3.54770	4.80040	C	-10.39300	-9.00200	5.22390
C	2.94900	2.96230	5.35480	H	-8.42790	-8.89250	4.34120
H	2.85310	2.11290	6.01980	H	-16.00300	-2.64300	4.95940
C	4.19420	3.46970	5.05320	C	-15.28180	-1.09060	6.24500
H	5.07200	3.00800	5.48830	H	-14.22460	0.33180	7.45740
C	4.35080	4.56040	4.20280	H	-10.39300	-10.08500	5.22390
C	3.20270	5.13450	3.65740	C	-16.64790	-0.54930	6.57300
H	3.29360	5.98280	2.99040	H	-16.95060	0.15710	5.80990
C	1.95180	4.63380	3.95690	H	-16.61850	-0.05220	7.53480
H	1.07070	5.09520	3.52830	H	-17.35910	-1.36520	6.61130

References

[1] G. Sheldrick, *University of Göttingen*, 1996.

[2] M. J. Frisch, G. W. Trucks, H. B. Schlegel, G. E. Scuseria, M. A. Robb, J. R. Cheeseman, G. Scalmani, V. Barone, G. A. Petersson, H. Nakatsuji, X. Li, M. Caricato, A. V. Marenich, J. Bloino, B. G. Janesko, R. Gomperts, B. Mennucci, H. P. Hratchian, J. V. Ortiz, A. F. Izmaylov, J. L. Sonnenberg, D. Williams-Young, F. Ding, F. Lipparini, F. Egidi, J. Goings, B. Peng, A. Petrone, T. Henderson, D. Ranasinghe, V. G. Zakrzewski, J. Gao, N. Rega, G. Zheng, W. Liang, M. Hada, M. Ehara, K. Toyota, R. Fukuda, J. Hasegawa, M. Ishida, T. Nakajima, Y. Honda, O. Kitao, H. Nakai, T. Vreven, K. Throssell, J. A. Montgomery Jr., J. E. Peralta, F. Ogliaro, M. J. Bearpark, J. J. Heyd, E. N. Brothers, K. N. Kudin, V. N. Staroverov, T. A. Keith, R. Kobayashi, J. Normand, K. Raghavachari, A. P. Rendell, J. C. Burant, S. S. Iyengar, J. Tomasi, M. Cossi, J. M. Millam, M. Klene, C. Adamo, R. Cammi, J. W. Ochterski, R. L. Martin, K. Morokuma, O. Farkas, J. B. Foresman and D. J. Fox, *Gaussian 16 Revision A.03*, 2016.

[3] S. Kozuch and J. M. L. Martin, *Phys. Chem. Chem. Phys.*, 2011, **13**, 20104.

[4] S. Kozuch and J. M. L. Martin, *J. Comput. Chem.*, 2013, **34**, 2327–2344.

[5] S. Grimme, J. Antony, S. Ehrlich and H. Krieg, *J. Chem. Phys.*, 2010, **132**, year.

[6] S. Grimme, S. Ehrlich and L. Goerigk, *J. Comput. Chem.*, 2011, **32**, 1456–1465.

[7] F. Weigend and R. Ahlrichs, *Phys. Chem. Chem. Phys.*, 2005, **7**, 3297.

[8] F. Birch, *Phys. Rev.*, 1947, **71**, 809–824.

[9] C. R. Groom, I. J. Bruno, M. P. Lightfoot and S. C. Ward, *Acta Crystallogr. Sect. B*, 2016, **72**, 171–179.

[10] R. G. Hicks, *Aust. J. Chem.*, 2001, **54**, 597.

[11] L. Norel, F. Pointillart, C. Train, L. M. Chamoreau, K. Boubekeur, Y. Journaux, A. Brieger and D. J. Brook, *Inorg. Chem.*, 2008, **47**, 2396–2403.

[12] V. Kumar, S. Shova, V. Maurel, G. Novitchi and C. Train, *Eur. J. Inorg. Chem.*, 2018, **2018**, 517–524.
